# Decoding drought tolerance from a genomic approach in *Castanea sativa* Mill

**DOI:** 10.1002/tpg2.70116

**Published:** 2025-11-09

**Authors:** A. Perez‐Rial, P. Castro, A. Solla, M. A. Martín, J. V. Die

**Affiliations:** ^1^ Department of Genetics‐ETSIAM Universidad de Córdoba Córdoba Spain; ^2^ Faculty of Forestry, Institute for Dehesa Research (INDEHESA) Universidad de Extremadura Plasencia Spain

## Abstract

Climate change, marked by prolonged periods of extreme summer drought coupled with heat, presents a significant challenge for chestnut forests. Genomic insight into drought tolerance in *Castanea sativa* is essential for enhancing the adaptation of this species to climate change. However, progress in this area has been hindered by the lack of a genome reference. To address this limitation, we developed a comprehensive drought tolerance gene atlas by leveraging publicly available databases and the high homology between the *Quercus* and *Castanea* genera. Candidate genes were identified through a mapping approach using short‐read sequence databases and validated via Sanger sequencing. Our method enabled the successful reconstruction of coding sequences and the identification of genetic variability in *C. sativa*. Two genes encoding an oleosin‐like protein and a two‐component response regulator exhibited significant sequence differences, suggesting their involvement in adaptive stress responses. These genes emerge as promising targets for future research and potential genetic markers for drought tolerance. The resulting gene atlas provides valuable insights into drought tolerance and supports the development of molecular markers for more targeted and effective conservation and breeding strategies.

## Core Ideas

- A gene atlas for drought tolerance in *Castanea sativa* was developed using genomic and comparative tools.
- We reconstructed a number of coding sequences and identified genetic tags for gene variability.
- Candidate genes *CG8* and *CG16* show significant variability, potentially linked to adaptive stress responses.
- Our approach provides a framework for studying genetic diversity in species lacking reference genomes.

## Plain Language Summary

Chestnut trees, essential for Mediterranean ecosystems, face challenges from increasing droughts and heatwaves. This study identifies key genes that may help chestnut trees adapt to these stresses. By comparing chestnut DNA sequences with related oak species, we found genes involved in stress responses. These genes could guide future breeding efforts to develop drought‐tolerant chestnut varieties, ensuring their survival and ecological contributions in a changing climate.

**Abbreviations**
CDS: coding sequence
EST‐SSRs: expressed sequence tag‐derived simple sequence repeats
GO: gene ontology
NCBI: national center for biotechnology information
PCR: polymerase chain reaction

## 1 INTRODUCTION

Climate change is driving significant shifts in the structure and functioning of forests worldwide (Heilmayr et al., 2023). Current climate scenarios consist of an increased frequency, duration, and intensity of drought episodes, largely driven by rising temperatures and altered precipitation patterns (Cook et al., 2020; Kovats et al., 2014; Trenberth, 2011). These scenarios and their interactions with forest pests and diseases are contributing to widespread decline of trees in many forest ecosystems (DeSoto et al., 2020; Kramer et al., 2010; McDowell et al., 2020).

Genetic adaptation is one of the mechanisms that can contribute to tree populations' ability to persist under changing climatic conditions, alongside phenotypic plasticity, migration to suitable environments, or combinations of these responses (Alberto et al., 2013; Fady et al., 2016). However, the emergence of unprecedented combinations of precipitation and temperature, often exceeding the historical ranges of many species, poses one of the most critical challenges (Burke et al., 2018; Williams & Jackson, 2007). Such excesses could increase species vulnerability as trees face extreme conditions exceeding the limits of their genetic tolerance (Harris et al., 2018; Pacifici et al., 2015). In this context, rapid warming may surpass the adaptive capacity of numerous tree species (Heilmayr et al., 2023).

The European chestnut (*Castanea sativa* Mill., 2*x *= 2*n *= 24) is the only chestnut species native to the Mediterranean basin. This region, which includes southern Europe, North Africa, and parts of the Near East, is recognized as one of the world's major biodiversity hotspots and is considered a priority for conservation (Medail & Quezel, 1999; Myers et al., 2000). In this context, chestnuts provide significant ecological and economic value, thriving in natural, semi‐natural, and managed ecosystems. As a member of the Fagaceae family, which also includes *Quercus* (oaks) and *Fagus* (beeches) genera, *Castanea sativa* contributes to the biodiversity of Mediterranean forests, with its populations exhibiting high genetic diversity (Mattioni et al., 2017; Pereira‐Lorenzo et al., 2019).

The Mediterranean region is particularly vulnerable to climate change, with ongoing environmental shifts imposing strong selective pressure on its forest, including on its populations of European chestnut (Freitas et al., 2021; Pérez‐Girón et al., 2020). While *C. sativa* is adapted to a wide range of climates and local environmental conditions, particularly in terms of precipitation and temperature, it remains highly susceptible to severe drought (Alcaide et al., 2019; Castellana et al., 2021; Dorado et al., 2022; Martin et al., 2010; Míguez‐Soto & Fernández‐López, 2015; Míguez‐Soto et al., 2019). This vulnerability is evidenced by reductions in growth rate, leaf conductance, photosynthetic activity, and fine root vitality under drought conditions, underscoring its sensitivity to climate stress (Camisón et al., 2019, 2021; Ciordia et al., 2012; Fernandes et al., 2022). Consequently, identifying candidate genes associated with drought tolerance in *C. sativa* would be of high interest.

**Core Ideas**

- A gene atlas for drought tolerance in *Castanea sativa* was developed using genomic and comparative tools.
- We reconstructed a number of coding sequences and identified genetic tags for gene variability.
- Candidate genes *CG8* and *CG16* show significant variability, potentially linked to adaptive stress responses.
- Our approach provides a framework for studying genetic diversity in species lacking reference genomes.

Research into the genetic basis of drought tolerance in *C. sativa* is an important avenue to investigate its ability to adapt in the face of changing climate conditions. Historically, the lack of genomic sequences has hindered the development of specific genetic markers and limited the analysis of candidate genes related to stress response. Most available expressed sequence tag‐derived simple sequence repeats (EST‐SSRs) markers are derived from *Quercus* species, which have been more extensively studied and exhibit high genetic synteny with *Castanea* (Alcaide et al., 2019; Castellana et al., 2021; Dorado et al., 2022; Martín et al., 2017; Staton et al., 2020). Consequently, much of the molecular research on chestnut adaptation has relied on *Quercus*‐based resources rather than species‐specific genomic tools, highlighting the need for more targeted genomic resources to advance our understanding of chestnut adaptation.

Recently, significant advances have been made with the release of genome sequences of Asian chestnut species, such as *Castanea mollissima* Blume and *Castanea crenata* Sieb. et Zucc., which offer invaluable resources for comparative genomics. Several *C. mollissima* varieties have been sequenced, with genome sizes ranging from 671.99 to 785.5 Mb (Hu et al., 2022; Qiao et al., 2024; Staton et al., 2020; Sun et al., 2020; Wang et al., 2020; Xing et al., 2019). Similarly, two pseudo‐chromosome‐level assemblies from recent studies reported genome sizes of 683.8 and 718.3 Mb for *C. crenata* (Shirasawa et al., 2021; Wang et al., 2022). This variation in genome size is consistent with intraspecific differences among chestnut cultivars and may reflect underlying structural variation, highlighting the need for comprehensive genomic resources across diverse genotypes (Hu et al., 2022). In addition, significant progress has been made in the publication and annotation of the American chestnut (*Castanea dentata* (Marshall) Borkh.) genome (*C. dentata* v1.1; https://phytozome‐next.jgi.doe.gov/info/Cdentata_v1).

At the onset of this study, no genome assemblies for *C. sativa* were published. In the nucleotide database of GenBank at NCBI (National Center for Biotechnology Information), only ∼950 sequences were available, with about 65% being mRNA fragments from expressed sequence tag libraries. To address this gap, we developed a method leveraging publicly available databases to create a comprehensive atlas of candidate genes for drought tolerance based on the high homology between the *Quercus* and *Castanea* genera. Candidate gene sequences were identified using a mapping approach based on short‐read sequence databases of *C. sativa* and validated through Sanger sequencing of genotypes from the Iberian Peninsula. The recent release of the first chromosome‐level genome assembly of *C. sativa* (Bianco et al., 2024) provided an excellent opportunity to validate our approach.

By integrating our data with the recently published European chestnut genome data, we performed a comparative analysis of coding regions, assessed genetic variability, and identified potential genetic markers associated with drought tolerance. This atlas will pave the way for the detailed characterization of candidate genes and provide a valuable knowledge base that could be integrated into breeding programs aiming to search drought‐tolerant chestnuts.

## 2 MATERIALS AND METHODS

### 2.1 Plant material

The two genotypes used in this study (Sur and Torito) were selected within the chestnut breeding program conducted by Universidad de Extremadura (Faculty of Forestry in Plasencia, Spain) because of their high tolerance to drought and heat stress, and resistance to *Phytophthora cinnamomi* (Solla et al., 2024). Sur originated from Paterna del Río (tree 18, code p11), Almería, southern Spain (37°01′54.0″ N, 2°57′15.5″ W), and Torito originated from Valle de Matamoros (tree 1, code s/id), Badajoz, western Spain (38°22′28.5″ N, 6°48′00.1″ W) (Solla et al., 2024). Their assignment as “pure” *C. sativa* and the absence of exotic *C. crenata* and *C. mollissima* germplasm were verified with 13 simple sequence repeat markers, following Alcaide et al. (2022).

### 2.2 Candidate genes selection and annotation

Comprehensive identification of drought tolerance candidate genes was achieved using selected genes from an RNA‐Seq collection, which represents the most complete *Quercus* spp. transcriptional profile under drought stress to date (Madritsch et al., 2019). *Quercus* genes were selected based on the following criteria: 80 genes with the highest expression levels in *Q. robur* (a drought‐susceptible species) and 80 genes with the highest expression levels in *Q. ilex* (a highly drought‐tolerant species). Additionally, 167 genes exhibiting expression patterns that correspond with the drought tolerance gradient observed across the *Quercus* species were included. After removing duplicates and keeping only one isoform per gene, a final set of 223 unique genes was obtained (Table S1). Protein sequences were retrieved from NCBI using the refseqR package (v.1.1.4) (Die, 2024) in R (v.4.2.0) (R Core Team, 2022). Functional annotation was performed using the gene ontology (GO) tool Blast2GO (Conesa & Götz, 2008), implemented in the OmicsBox platform (v.3.0.27), to assign GO terms to each gene. The following settings were applied: BLASTp against the NCBI RefSeq protein database, *E*‐value filter ≤ 10^−6^, HSP (highest scoring pair) length cutoff of 33, maximum of 10 BLAST hits per sequence, and an annotation cutoff of 50. To further enhance annotation, InterProScan results were merged with GO terms, and GOSlim plant‐specific terms were assigned. Finally, genes with GO terms “response to stimuli,” “response to stress,” “signal transduction,” and “transcription factor” were selected as candidates for identifying homologous genes in the *Castanea* genus.

### 2.3 Identification of homologous genes in the *Castanea* genus

Homologs were first identified in the closely related Asian species *C. mollissima* and *C. crenata* using the NCBI's BLASTp tool (Altschul et al., 1990). Protein sequences of the candidate genes were used as queries against the protein databases of *C. mollissima* cultivar N11‐1 (Wang et al., 2020) and *C. crenata* (Shirasawa et al., 2021), available in the Castanea Genome Database (http://castaneadb.net/). To ensure accurate identification, a reciprocal BLASTp search between the two *Castanea* species was performed. Full‐length nucleotide sequences of the homologous genes were aligned using Geneious Prime (v.2024.0) with default parameters to generate consensus sequences for each gene. Sequence homology was assessed based on identity (i.e., the percentage of pairwise residues that are identical in the alignment) and coverage (i.e., the proportion of the largest sequence covered by the smallest one) across the annotated coding sequence (CDS).

### 2.4 Construction of *C. sativa* in silico sequences through short‐read mapping

The consensus sequences obtained from the Asian species were used to retrieve short reads from *C. sativa* from the NCBI sequence read archive (SRA database) SRX14087670 (Bioproject Accession PRJNA804196; Sandercock et al., 2022). This database was generated from DNA extracted from the leaves of an individual tree in Portugal. These reads were mapped to the query sequences using Geneious Prime (v.2024.0) with default parameters, low sensitivity, and no maximum read limit (Perez‐Rial et al., 2024). The reliability of the mapping process was assessed based on the annotated CDSs of *C. mollissima* and *C. crenata*, by using (1) mapping identity (i.e., percentage of columns in the alignment where all sequences are identical), (2) mapping coverage (i.e., proportion of the query sequence covered by one or more reads), and (3) average number of reads mapped to each site. To confidently call a base in the *C. sativa* in silico sequence, a threshold of 65% mapping identity was applied, and a minimum of two reads per site was required. Positions with fewer than two reads were designated as “N.” For this study, sequences with mapping identity and/or coverage values below 85% were considered to exhibit substantial variability based on observed results.

### 2.5 Primer design for Sanger sequencing

To validate the in silico CDS, Sanger sequencing was performed on half of the candidate genes. This subset was selected to confirm the accuracy of the in silico predictions, as our primary goal was to obtain coding gene sequences. Primers were designed by using Geneious Prime (v.2024.0), based on a consensus sequence derived from aligning sequences of *C. mollissima*‐N11, *C. crenata*, and the inferred sequence of *C. sativa*. Primers were targeted to the most conserved regions flanking each CDS. When discrepancies between the annotated CDS of *C. mollissima* and *C. crenata* were observed, primers were designed based on the regions with longer annotated CDS (Figure S1). The primers were designed with the following criteria: polymerase chain reaction (PCR) amplicon lengths of 300–850 bp (preferably around 750 bp), Tm of 60 ± 3°C, primer lengths of 19–23 nucleotides, and guanine–cytosine content of 40%–80%. If a single primer pair could not cover the entire CDS, additional primer pairs were designed with approximately 100 bp overlaps between amplicons. A total of 51 primer pairs were designed, ensuring comprehensive coverage of the 45 putative CDS regions of the selected genes (Table S2).

### 2.6 DNA extraction and PCR amplification

Genomic DNA was extracted from 18 to 20 mg of lyophilized leaves of Sur and Torito genotypes according to the DNeasy Plant Mini Kit protocol (Qiagen). PCR amplifications were performed for each genotype using the GoTaq DNA Polymerase kit (Promega). PCR reactions were performed in a total volume of 20 µL, containing 20 ng of genomic DNA, 0.65 µM of each primer, 0.2 mM of dNTPs, 2.5 mM of MgCl2, 1X Colorless GoTaq Flexi Buffer, and 0.04 U/µL of GoTaq DNA Polymerase. The amplification conditions were an initial heat activation at 95°C for 15 min, followed by 28 cycles consisting of 45 s of denaturation at 95°C, 75 s of annealing at 60°C, and 45 s of extension at 72°C.

### 2.7 PCR product purification, Sanger sequencing, and CDS reconstruction

**FIGURE 1:**
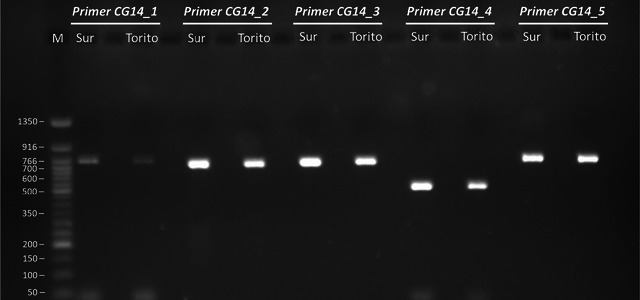
Amplification of the five primer pairs designed for Sanger sequencing of the “heat shock protein 83” locus (*CG14*) in two different genotypes of *Castanea sativa* (Sur and Torito). The band size of the molecular weight marker (M) is expressed in bp.

To confirm successful amplification, PCR products were verified by agarose gel electrophoresis (Figure 1). Products showing a single clear band were cleaned with Exo‐CIP Rapid PCR Cleanup (New England Biolabs) according to the manufacturer's instructions. If multiple bands were present, the target band was excised and purified using the Zymoclean Gel DNA Recovery Kit (Zymo Research). Sanger sequencing was performed by STABVIDA.

The sequencing results were analyzed using Geneious Prime (v.2024.0) software. The resulting reads were aligned to the in silico sequence of *C. sativa* to reconstruct the CDS and generate a reference sequence for the two chestnut genotypes. Regions with a base‐calling error probability greater than 2% were trimmed to ensure accuracy and reliability.

### 2.8 Identification of the candidate genes in the new *C. sativa* reference genome

During the reconstruction of the gene sequences, the first *C. sativa* genome from the Italian cultivar ‘Marrone di Chiusa Pesio’ was published (Bianco et al., 2024). The assembly was performed in two haplotypes, each corresponding to one of the phased chromosome sets of the same diploid individual, providing insight into allelic variation within its genome. Taking advantage of this significant achievement, the CDS obtained for each candidate gene was used as a query in BLASTn against the CDS of haplotypes 1 and 2 to identify the homologous genes. The available CDS sequences obtained through different methodologies, including in silico reconstruction (short‐read mapping), Sanger sequencing (when available), and the *C. sativa* reference genome, were aligned to assess pairwise identity and detect variable regions. A 90% pairwise identity threshold was applied to consider sequences as highly conserved. Since these CDSs may serve as genetic markers for drought tolerance in *C. sativa*, they were screened to identify potential microsatellite markers using the default parameters of the Microsatellite Repeats Finder program (http://insilico.ehu.es/mini_tools/microsatellites/). Finally, the identified microsatellite regions were examined for variability within the CDS sequences obtained in our study.

A concise summary of the key methodological steps involved in candidate gene identification, sequence reconstruction, and validation is provided in Table 1.

**TABLE 1:** Summary of the methodological pipeline used for candidate gene identification and validation in *Castanea sativa*.

Step (section)	Description
**1 (2.2)**	Selection of drought‐responsive genes from *Quercus* RNA‐Seq data (Madritsch et al., 2019).
**2 (2.2)**	Filtering of genes based on GO terms related to stress, signaling, and transcription regulation.
**3 (2.3)**	Identification of homologs in *C. mollissima* and *C. crenata* via reciprocal BLASTp and generation of consensus sequence.
**4 (2.4)**	In silico CDS reconstruction using *C. sativa* short‐read data (SRA: SRX14087670).
**5 (2.5)**	Primer design based on aligned *Castanea* sequences for Sanger sequencing.
**6 (2.6 and 2.7)**	PCR amplification and Sanger sequencing in two *C. sativa* genotypes.
**7 (2.7)**	Validation of the in silico CDS reconstruction approach.
**8 (2.8)**	Comparison with the *C. sativa* reference genome and detection of sequence variation and microsatellites.

Abbreviations: CDS, coding sequence; GO, gene ontology; PCR, polymerase chain reaction.

## 3 RESULTS

### 3.1 Candidate genes selection and identification in Asian *Castanea*

**FIGURE 2:**
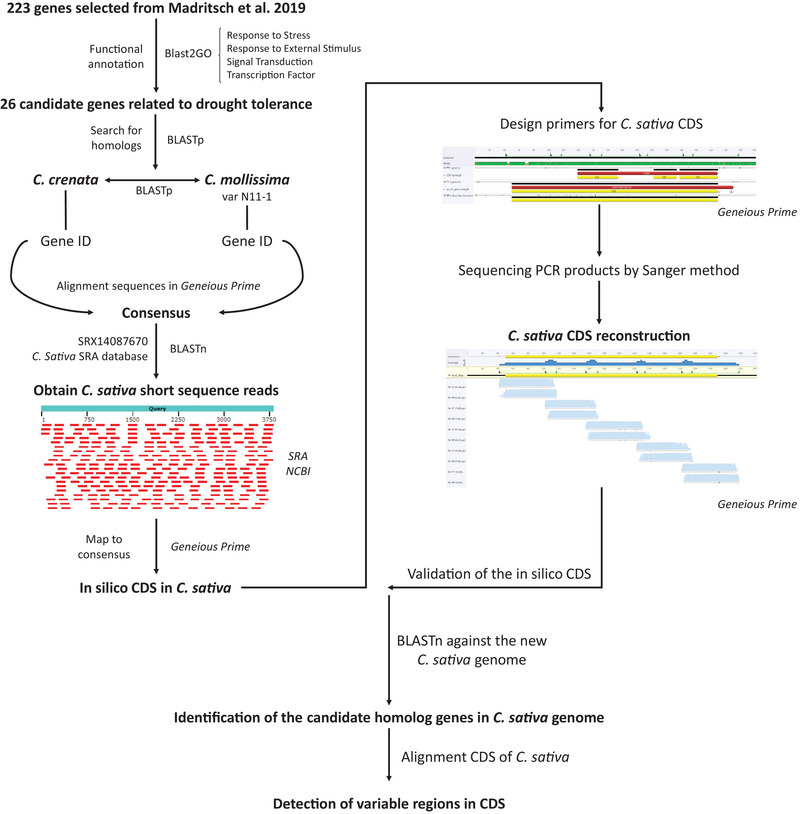
Workflow for obtaining the coding sequence (CDS) of candidate genes related to drought tolerance in *Castanea sativa*. The process began with the selection of 26 candidate genes (Madritsch et al., 2019) which were subjected to BLASTp searches to identify homologs in the *Castanea mollissima* and *Castanea crenata* genomes. The results were validated through reciprocal BLASTp. The consensus sequence, obtained by aligning sequences from both *Castanea* species, was then used to query the NCBI sequence read archive (SRA) database (SRX14087670) to obtain short‐reads from *C. sativa*. The reads were mapped to generate the putative coding sequences. Primers were designed based on a new consensus sequence derived from aligning the sequences of *C. mollissima*‐N11, *C. crenata*, and the inferred *C. sativa* sequences, flanking the annotated CDS regions. Polymerase chain reaction (PCR) products were sequenced and aligned to validate the putative CDS. Finally, the CDS sequences were used in BLASTn searches to identify homologous genes in the *C. sativa* genome assembly and detect variable regions, facilitating the discovery of potential genetic markers.

A total of 26 candidate genes were selected based on their annotation by GO terms. Three genes were identified as “transcription factors,” three were classified as “signal transduction” genes, and the remaining 20 were annotated with terms related to “response to stimulus,” “response to stress” or both (Table 2). The process used to obtain the CDS of these candidate genes, potentially associated with drought tolerance in *C. sativa*, and to identify genetic variations is summarized in Figure 2.

**TABLE 2:** Description of the selected 26 candidate genes with their gene ontology (GO) annotation.

ID Candidate gene	ID [*Quercus suber*]	Description	GO terms ID	Response to stress	Response to stimulus	Signal transduction	Transcription factor
***CG1***	POE45809.1	Probable disease resistance protein At1g58602	P:GO:0006950; P:GO:0009605; P:GO:0009607; F:GO:0000166	x	x		
***CG2***	POE54072.1	Cytochrome P450 CYP82D47‐like	P:GO:0006950; P:GO:0009605; P:GO:0009607; F:GO:0003824; F:GO:0005488	x	x		
***CG3***	POE84274.1	Uncharacterized protein LOC115982017	P:GO:0007165; F:GO:0005515			x	
***CG4***	POE91933.1	Heat shock cognate protein 80‐like	P:GO:0006950; P:GO:0009628; P:GO:0009987; F:GO:0000166; F:GO:0005515; F:GO:0016787; C:GO:0005829; C:GO:0005886	x	x		
***CG5***	POE93777.1	Wall‐associated receptor kinase 2‐like	P:GO:0007165; P:GO:0036211; F:GO:0000166; F:GO:0016301; C:GO:0005886			x	
***CG6***	POF19195.1	Putative peroxidase 48	P:GO:0006950; F:GO:0003824; F:GO:0005488	x			
***CG7***	XP_021828467.1	Putative inactive disease susceptibility protein LOV1	P:GO:0006950; P:GO:0009605; P:GO:0009607; F:GO:0000166; F:GO:0005515	x	x		
***CG8***	XP_023765886.1	Oleosin 5‐like	P:GO:0000003; P:GO:0006950; P:GO:0009628; P:GO:0009791; P:GO:0009987; C:GO:0005622; C:GO:0016020	x	x		
***CG9***	XP_023874372.1	Alpha‐dioxygenase 1‐like	P:GO:0006629; P:GO:0006950; P:GO:0009058; P:GO:0009987; P:GO:0042221; F:GO:0003824; F:GO:0005488	x	x		
***CG10***	XP_023875502.1	Protein DETOXIFICATION 16‐like isoform X1	P:GO:0006810; P:GO:0009987; P:GO:0042221; F:GO:0005215; C:GO:0016020		x		
***CG11***	XP_023879721.1	Heat shock cognate protein 80‐like	P:GO:0006950; P:GO:0009628; P:GO:0009987; F:GO:0000166; F:GO:0005515; F:GO:0016787; C:GO:0005829; C:GO:0005886	x	x		
***CG12***	XP_023881819.1	ATP‐dependent DNA helicase 2 subunit KU70	P:GO:0006259; P:GO:0006950; P:GO:0009628; P:GO:0016043; F:GO:0000166; F:GO:0003677; F:GO:0016787; C:GO:0005634; C:GO:0016020	x	x		
***CG13***	XP_023888322.1	WRKY transcription factor WRKY24‐like	F:GO:0003677; F:GO:0003700; C:GO:0005634				x
***CG14***	XP_023890186.1	Heat shock protein 83	P:GO:0006950; P:GO:0009628; P:GO:0009987; F:GO:0000166; F:GO:0005515; F:GO:0016787; C:GO:0005829; C:GO:0005886	x	x		
***CG15***	XP_023891506.1	Probable disease resistance RPP8‐like protein 2 isoform X3	P:GO:0006950; P:GO:0009605; P:GO:0009607; F:GO:0000166	x	x		
***CG16***	XP_023903646.1	Two‐component response regulator ARR2‐like isoform X1	F:GO:0003677; F:GO:0003700; C:GO:0005634; C:GO:0016020				x
***CG17***	XP_023908969.1	Transcription factor TCP9	F:GO:0003677; F:GO:0003700; C:GO:0005634; C:GO:0016020				x
***CG18***	XP_023909438.1	Probable glutathione S‐transferase	P:GO:0008152; P:GO:0009987; P:GO:0042221; F:GO:0016740; C:GO:0005737		x		
***CG19***	XP_023911981.1	Putative disease resistance RPP13‐like protein 3	P:GO:0006950; P:GO:0009605; P:GO:0009607; F:GO:0000166	x	x		
***CG20***	XP_023915745.1	Serine/threonine‐protein kinase SRK2A‐like isoform X2	P:GO:0007165; P:GO:0036211; F:GO:0000166; F:GO:0016301; C:GO:0005737			x	
***CG21***	XP_023917730.1	UV‐stimulated scaffold protein A homolog	P:GO:0006259; P:GO:0006950; P:GO:0009416; F:GO:0005515; C:GO:0005622	x	x		
***CG22***	XP_023919090.1	UV‐stimulated scaffold protein A homolog	P:GO:0006259; P:GO:0006950; P:GO:0009416; F:GO:0005515; C:GO:0005622	x	x		
***CG23***	XP_023919293.1	MLO‐like protein 6	P:GO:0006950; P:GO:0009607; F:GO:0005515; C:GO:0016020	x	x		
***CG24***	XP_023924075.1	Protein argonaute 5‐like	P:GO:0006139; P:GO:0006950; P:GO:0009605; P:GO:0009607; F:GO:0003723; F:GO:0004518; F:GO:0005515; C:GO:0005737	x	x		
***CG25***	XP_023929726.1	Putative disease resistance protein RGA3	P:GO:0006950; P:GO:0009605; P:GO:0009607; F:GO:0000166	x	x		
***CG26***	XP_023929862.1	Probable disease resistance RPP8‐like protein 2	P:GO:0006950; P:GO:0009605; P:GO:0009607; F:GO:0005515	x	x		

*Note*: An “x” in a given cell under a specific column indicates that the gene's functional annotation is associated with that category.

The 26 candidate genes were subjected to BLASTp searches on NCBI to identify homologs in the genomes of *C. mollissima* and *C. crenata*. The results were validated through reciprocal BLASTp analysis (Table 3). Homologous genes for *CG5*, *CG9*, and *CG15* were identified in only one of the Asian *Castanea* species. For the remaining 23 genes, alignments yielded consensus sequences with an average identity of 98.8% (ranging from 96.5% to 99.95%) and a coverage of 89.6% (ranging from 49% to 100%). Notably, *CG12* corresponds to three consecutive genes in *C. crenata* (*Ccr1.0Dg1284.1*, *Ccr1.0Dg1285.1*, *Ccr1.0Dg1286.1*) and matches a single gene in *C. mollissima* (*GWHTANWH024811*). Overall, the results indicate a high level of conservation of CDS genes between the two Asian *Castanea* species.

**TABLE 3:** Identified genes in *Castanea crenata* and *Castanea mollissima*, results of coding sequence (CDS) alignments and results of mapping with short‐read sequences (from SRX14087670 *Castanea sativa* database).

ID	ID (*C. crenata*)	ID (*C. mollissima*)	Alignment *Castanea* CDS		Short‐read sequences mapped to *Castanea* consensus		
			Id. (%)	Cov. (%)	Id. (%)	Cov. (%)	Number of short‐reads (mean ± SD)
***CG1***	*Ccr1.0Ag1556.1*	*GWHTANWH005259*	99.8	49	99.4	100	9.3 ± 4.7
***CG2***	*Ccr1.0Lg4137.1*	*GWHTANWH033450*	96.5	99	97.7	100	14.1 ± 11.0
***CG3***	*Ccr1.0Cg2558.1*	*GWHTANWH017466*	99.5	100	98.6	99	10.3 ± 3.4
***CG4***	*Ccr1.0Eg4723.1*	*GWHTANWH008338*	99.95	100	99.4	100	10.0 ± 4.4
***CG5*** ^a^, ^b^	–	*GWHTANWH012906*	–	–	99.2	75	8.7 ± 2.9
***CG6***	*Ccr1.0Ag1260.1*	*GWHTANWH005352*	99.3	100	94.8	100	24.3 ± 10.1
***CG7***	*Ccr00g1756.1*	*GWHTANWH006379*	99.2	100	96.0	99	11.9 ± 5.6
***CG8*** ^b^	*Ccr1.0Dg4334.1*	*GWHTANWH026118*	98.6	100	79.4	81	19.2 ± 15.0
***CG9*** ^a^	*Ccr1.0Ag7092.1*	–	–	–	98.0	100	17.9 ± 8.5
***CG10***	*Ccr1.0Hg4955.1*	*GWHTANWH024021*	98.4	86	97.5	97	26.5 ± 15.2
***CG11***	*Ccr1.0Fg0329.1*	*GWHTANWH021658*	99.4	100	99.2	100	8.4 ± 4.5
***CG12***	c	*GWHTANWH024811*	97.4	72	98.1	100	30 ± 33.0
***CG13***	*Ccr1.0Hg3066.1*	*GWHTANWH022813*	99.0	91	99.0	100	10.6 ± 4.3
***CG14***	*Ccr1.0Jg3185.1*	*GWHTANWH011982*	99.9	96	99.3	100	10.1 ± 4.3
***CG15*** ^a^	–	*GWHTANWH006369*	–	–	98.0	93	9.0 ± 4.2
***CG16*** ^b^	*Ccr1.0Eg0631.1*	*GWHTANWH006516*	97.8	83	99.1	83	29.1 ± 18.8
***CG17***	*Ccr1.0Fg5431.1*	*GWHTANWH019191*	98.5	100	98.9	99	8.0 ± 3.5
***CG18***	*Ccr1.0Bg2126.1*	*GWHTANWH027503*	99.7	99	96.9	100	15.6 ± 6.0
***CG19***	*Ccr1.0Ag1554.1*	*GWHTANWH005261*	99.7	87	98.9	100	7.5 ± 3.7
***CG20***	*Ccr1.0Dg1757.1*	*GWHTANWH024996*	97.6	93	99.5	97	7.7 ± 5.7
***CG21***	*Ccr1.0Gg1654.1*	*GWHTANWH010553*	99.1	99	98.0	100	17.2 ± 6.7
***CG22***	*Ccr1.0Bg4790.1*	*GWHTANWH028767*	98.6	99	98.0	100	17.4 ± 6.8
***CG23***	*Ccr1.0Ig0407.1*	*GWHTANWH029277*	97.1	94	98.4	100	12.2 ± 6.1
***CG24***	*Ccr1.0Hg4160.1*	*GWHTANWH023384*	99.4	79	97.6	100	10.2 ± 4.4
***CG25***	*Ccr1.0Eg6269.1*	*GWHTANWH009140*	99.1	80	93.8	91	13.5 ± 6.3
***CG26***	*Ccr00g2515.1*	*GWHTANWH006355*	99.3	55	98.1	100	11.5 ± 5.5

Abbreviations: Id., Identity; Cov., coverage.

^a^ Genes found in one Asian chestnut species only.

^b^ Genes with mapping identity and/or coverage <85%.

^c^ Three consecutive genes are identified as homologous in *C. crenata*: *Ccr1.0Dg1284.1*, *Ccr1.0Dg1285.1*, and *Ccr1.0Dg1286.1*.

### 3.2 Short‐read mapping and Sanger sequencing in *C. sativa*

The reads from *C. sativa* for each gene were mapped to the query sequences used to derive the in silico putative CDS of the candidate genes (Data S1), with an average mapping identity of 97% (ranging from 79.4% to 99.5%) and an average mapping coverage of 97% (ranging from 74.5% to 100%) relative to the annotated CDS. On average, 14.2 ± 7.9 short‐read sequences were mapped per coverage position (Table 3). Only three genes (*CG5*, *CG8*, and *CG16*) showed less than 85% mapping coverage for the CDS. While *CG8* and *CG16* showed high identity and coverage across Asian chestnut species, *CG5* was found exclusively in *C. mollissima*.

**FIGURE 3:**
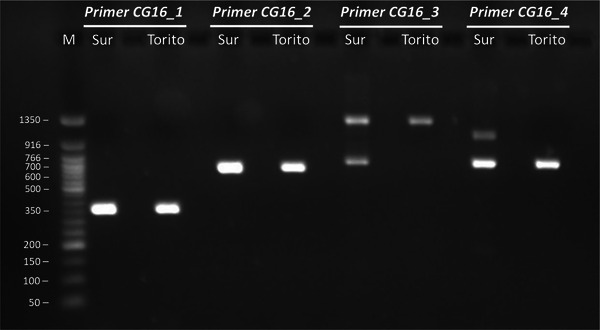
Amplification of the four primer pairs designed for Sanger sequencing of the “two‐component response regulator ARR2‐like” locus (*CG16*) in two different genotypes of *Castanea sativa* (Sur and Torito). The band size of the molecular weight marker (M) is expressed in bp. There are differences in the band size depending on the genotype for products CG16_3 and CG16_4.

Half of the 26 candidate genes were sequenced by using the Sanger method to validate the in silico predictions. Band sizes were consistent with theoretical predictions in both *C. sativa* genotypes, with notable exceptions for *CG16* and *CG8*, which showed substantial deviations (Table S3). Specifically, *CG16* band sizes were inconsistent between genotypes (Figure 3). The theoretical band sizes were 706 bp for the CG16_3 primer product (CG16_3_700) and 728 bp for the CG16_4 primer product (CG16_4_720), consistent with predictions for Asian chestnut species. However, additional higher bands were observed in Sur, that is, approximately 1300 bp for the CG16_3 primer (CG16_3_1300) and 1050 bp for the CG16_4 primer (CG16_4_1050). In contrast, the Torito genotype showed only one band for each primer, CG16_3_1300 and CG16_4_720. For *CG8*, the PCR product sizes were shorter than expected for both primer pairs used, that is, 360 and 500 bp, compared to the expected sizes of 638 and 798 bp for CG8_1 and CG8_2, respectively. The results of short‐read mapping revealed coverage values below 85% for both *CG16* and *CG8* (83% and 81%, respectively). Together, the observed deviations in PCR product sizes and reduced coverage suggest significant variability in these two genes within *C. sativa*. The remaining 11 Sanger‐sequenced genes were successfully reconstructed (Supporting information S2). The difference between expected (in silico) and observed (sequenced) PCR product sizes averaged 46 ± 18 bp, likely attributable to minor technical sequencing inaccuracies at the PCR product ends. The high identity (average 98.9%) and coverage (average 98.3%) observed between the sequenced CDS and those obtained from short‐read mapping validate the in silico approach, demonstrating the effectiveness of using short reads for reconstructing CDSs (Table S4).

### 3.3 Candidate genes identification in the *C. sativa* reference genome and CDS variation analysis

The obtained CDSs were used as queries in BLASTn searches to identify homologous genes in the two available haplotype databases for *C. sativa* Marrone di Chiusa Pesio. The genes identified for each *C. sativa* haplotype are listed in Table 4. The in silico putative CDS derived from short‐read mapping and the available Sanger‐sequenced CDS, where applicable, were aligned with the annotated CDS of homologous genes from each haplotype to assess pairwise identity (Table 4). For most genes, pairwise identity exceeded 90%, except for *CG10* (81.2%) and *CG25* (62.6%). *CG10*, a “protein detoxification 16‐like” homolog, exhibited notable differences in exon annotation. In *C. crenata* and *C. mollissima*‐N11, a single accession ID appeared to correspond to two consecutive genes in the *C. sativa* haplotypes, which showed differences in exon arrangement. Specifically, haplotype 1 included 6 + 9 exons, while haplotype 2 had 8 + 6. For *CG25*, no annotated gene was found in haplotype 2, and the region only moderately aligned with *CG25* from haplotype 1 and the CDS obtained through mapping in our study. Additional minor discrepancies in exon structural annotation were also observed for *CG6*, *CG15*, and *CG19* between the two *C. sativa* haplotypes. For example, *CG15* was annotated with three exons in haplotype 1 and as two separate genes (2 + 1 exons) in haplotype 2, while still maintaining over 90% identity.

**TABLE 4:** Homologous candidate genes in the recent *Castanea sativa* genomes (haplotype 1 and haplotype 2). The identity reflects the annotated coding sequence (CDS)’s conservation by aligning the available CDS sequences.

Candidate gene		Haplotype 1		Haplotype 2		
	ID	ID (Chromosome: position)	Number of exon//CDS length (bp)	ID (Chromosome: position)	Number of exon//CDS length (bp)	Pairwise identity (%)
Sanger—sequenced genes	***CG1***	*CSH1‐1g01369* (Chr1: 15,140,226–15,143,018)	1//2793	*CSH2‐1g01373* (Chr1: 15,119,306–15,122,098)	1//2793	98.8
	***CG2***	*CSH1‐12g57748* (Chr12: 47,269,210–47,271,600)	2//1569	*CSH2‐12g58251* (Chr12: 47,241,233–47,243,623)	2//1569	98.8
	***CG4***	*CSH1‐5g25874* (Chr5: 63,079,355–63,082,359)	3//2124	*CSH2‐5g25953* (Chr5: 60,999,498–61,002,535)	3//2124	99.9
	***CG6***	*CSH1‐1g01227* (Chr1: 13,450,314–13,447,853)	5//1086	*CSH2‐1g01217* (Chr1: 13,431,942–13,429,481)	4//1149	95.6
	***CG8***	*CSH1‐4g20534* (Chr4: 44,130,329–44,129,737)	3//420	*CSH2‐4g20663* (Chr4: 44,596,266–44,595,702)	2//477	^a^
	***CG11***	*CSH1‐6g27580* (Chr6: 2,524,536–2,527,560)	3//2118	*CSH2‐6g27692* (Chr6: 2,522,320–2,525,346)	3//2118	99.6
	***CG13***	*CSH1‐8g39109* (Chr8: 31,517,648–31,514,575)	5//1806	*CSH2‐8g39319* (Chr8: 31,465,007–31,461,934)	5//1806	99.7
	***CG14***	*CSH1‐10g47573* (Chr10: 29,133,821–29,130,805)	4//2118	*CSH2‐10g47831* (Chr10: 29,114,331–29,111,307)	4//2118	99.9
	***CG16***	*CSH1‐5g21915* (Chr5: 10,200,654–10,205,612)	5//1491	Region not present	–	^a^
	***CG17***	*CSH1‐6g32035* (Chr6: 54,756,437–54,755,370)	1//1068	*CSH2‐6g32171* (Chr6: 54,726,860–54,725,793)	1//1068	97.9
	***CG18***	*CSH1‐2g09662* (Chr2: 23,291,148–23,289,649)	2//672	*CSH2‐2g09736* (Chr2: 23,255,225–23,253,726)	2//672	99.8
	***CG20***	*CSH1‐4g18507* (Chr4: 18,085,656–18,080,212)	9//1071	*CSH2‐4g18593* (Chr4: 18,585,041–18,579,554)	9//1071	96.4
	***CG21***	*CSH1‐7g34131* (Chr7: 18,498,020–18,495,644)	2//1983	*CSH2‐7g34233* (Chr7: 17,905,811–17,903,435)	2//1983	99.0
Not Sanger—sequenced genes	***CG3***	*CSH1‐3g14646* (Chr3: 28,904,075–28,911,978)	6//1941	*CSH2‐3g14708* (Chr3: 28,866,360–28,874,280)	6//1935	99.1
	***CG5***	*CSH1‐10g45707* (Chr10: 8,483,673–8,479,812)	3//1743	*CSH2‐10g45935* (Chr10: 8,475,807–8,471,944)	3//1743	98.5
	***CG7***	*CSH1‐5g21540* (Chr5: 4,830,043–4,833,747)	1//3075	*CSH2‐5g21588* (Chr5: 3,909,161–3,905,457)	1//3075	98.8
	***CG9***	*CSH1‐1g05829* (Chr1: 69,068,940–69,074,774)	10//1917	*CSH2‐1g05896* (Chr1: 68,964,896–68,970,779)	10 // 1917	99.0
	***CG10***	*CSH1‐8g40987* (Chr8: 53,968,987–53,975,108); *CSH1‐8g40988* (Chr8: 53,978,948–53,986,632)	6//1137; 9//1671	*CSH2‐8g41169* (Chr8: 53,692,202–53,698,382); *CSH2‐8g41170* (Chr8: 53,704,391–53,709,030)	8//1281; 6//1398	81.2
	***CG12***	*CSH1‐4g18184* (Chr4: 13,265,074–13,242,681)	19//1887	*CSH2‐4g18265* (Chr4: 13,768,649–13,746,264)	19//1887	94.6
	***CG15***	*CSH1‐5g21515* (Chr5: 4,344,466–4,340,525)	3//3696	*CSH2‐5g21620* (Chr1: 4,394,638–4,397,534); *CSH2‐5g21621* (Chr1: 4,397,853–4,398,167)	2//2676; 1//315	96.2
	***CG19***	*CSH1‐1g01367* (Chr1: 15,129,445–15,126,553)	2//2775	*CSH2‐1g01371* (Chr1: 15,108,651–15,105,751)	3//2751	92.9
	***CG22***	*CSH1‐2g12012* (Chr2:51,644,520–51,647,132)	2//1956	*CSH2‐2g12107* (Chr2: 51,567,670–51,570,282)	2//1956	98.9
	***CG23***	*CSH1‐9g41543* (Chr9: 4,009,725–4,016,104)	15//1713	*CSH2‐9g41735* (Chr9: 3,999,705–4,006,110)	15//1713	98.9
	***CG24***	*CSH1‐8g40067* (Chr8: 43,569,471–43,577,027)	21//3024	*CSH2‐8g40255* (Chr8: 43,497,426–43,504,942)	21//3003	98.9
	***CG25***	*CSH1‐5g27331* (Chr5: 81,402,617–81,398,690)	4//3021	Unannotated gene (Chr5: 79,295,580–79,299,393)	–	62.6
	***CG26***	*CSH1‐5g21441* (Chr5: 3,499,521–3,495,607)	2//3594	*CSH2‐5g21555* (Chr5: 15,140,226–15,143,018)	2//3495	94.6

^a^ Region with high differences between the two haplotypes from the genome assembly and the two genotypes used in this study.

Notable differences between *C. sativa* haplotypes were also observed for the two genes that previously presented inconsistencies in mapping and PCR amplification: *CG8* (“oleosin 5‐like”) and *CG16* (“two‐component response regulator ARR2‐like”). The candidate gene *CG8*, located on chromosome 4, showed differences in annotated CDS between haplotypes, containing three exons totaling 420 bp in haplotype 1 and two exons totaling 477 bp in haplotype 2. Two primer pairs were designed to sequence the CDS of *CG8*, with expected product sizes of 638 bp for primer CG8_1 and 798 bp for CG8_2, closely matching those observed in Asian *Castanea* species (638 and 796 bp). Using the updated *C. sativa* genome, the expected sizes for primer CG8_2 were 388 bp for haplotype 1 and 335 bp for haplotype 2, while the expected size for CG8_1 remained close to the initial estimate (∼635 bp). For both CG8_1 and CG8_2 primers, PCR products for the Sur and Torito genotypes were around 320 bp. The reduction in size for primer CG8_2 was attributed to the deletion of a high‐repetition region containing an oleosin domain (Figure S2).

**FIGURE 4:**
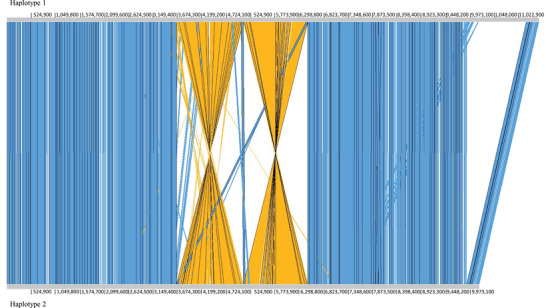
Genome variation in a region of chromosome 5 between haplotype 1 (CSH1‐chr5: 1–11,455,420) and haplotype 2 (CSH2‐chr5: 1–10,171,000) assemblies of *Castanea sativa*. Blue blocks represent aligned regions. There are two consecutive inversions (orange blocks), one located from approximately 3.7–5 Mb (CSH1‐chr5: 3,795,373–5,046,405 and CSH2‐chr5: 3,693,006–5,080,743) and the other from 5.2 to 6.4 Mb (CSH1‐chr5: 5,255,881–6,414,969 and CSH2‐chr5: 5,155,099–6,423,723). There is a deletion in haplotype 2 corresponding to the region of the haplotype 1 chr5, circa 9.9–11.2 Mb. Chromosome alignment performed with Act Artemis (v.18.2.0) using identity ≥ 90% and alignment score ≥10,500.

We identified structural rearrangements among haplotypes on chromosome 5, including a major deletion and two consecutive inversions (Figure 4). The candidate gene *CG16* was located in this region on chromosome 5 that was present in haplotype 1, but absent in haplotype 2 due to this deletion. This region spanned approximately 1.3 Mb and contained 86 genes (CSH1‐chr5: 9,909,454–11,184,824). Using a reciprocal BLASTn approach, only one gene from this area in haplotype 1 (CSH1‐5g21888) was identified as having a homolog in haplotype 2 (CSH2‐5g21842; Identity = 97.8%, Coverage = 100%, *E*‐value = 0). The remaining 85 genes in this region were deleted in haplotype 2. Additionally, two consecutive inversions were identified near the major deletion. The first inversion spanned approximately 3.7–5 Mb (CSH1‐chr5: 3,795,373–5,046,405 and CSH2‐chr5: 3,693,006–5,080,743), while the second extended from about 5.2 to 6.4 Mb (CSH1‐chr5: 5,255,881–6,414,969 and CSH2‐chr5: 5,155,099–6,423,723). The chromosome 5 in the genome of the Italian *C. sativa* cultivar (Bianco et al., 2024) showed the largest size differences within haplotypes, with haplotype 1 measuring 81.89 Mb and haplotype 2 measuring 79.79 Mb, a difference of over 2 Mb.

Interestingly, *CG16* also exhibited different band sizes between the Sur and Torito genotypes amplified with CG16_3 and CG16_4 primer pairs (Figure 3). For haplotype 1, the expected band sizes were approximately 1300 bp with primer CG16_3 (CG16_3_1300) and 1055 bp with primer CG16_4 (CG16_4_1050). These sizes aligned with the higher bands observed in Sur (CG16_3_1300 and CG16_4_1050), as well as the band CG16_3_1300 detected in Torito (Figure 3). Using the recently sequenced *C. sativa* genome, we ruled out nonspecific amplifications, confirming that these primers specifically targeted *CG16*. These results suggest that the Sur genotype is heterozygous for this gene, with one allele resembling those of Asian chestnuts (CG16_3_700 and CG16_4_720) and the other corresponding to haplotype 1 (CG16_3_1300 and CG16_4_1050). In contrast, the Torito genotype appeared homozygous yet distinct from Sur, showing CG16_3_1300 and CG16_4_720 bands. The CG16_4_720 band is consistent with Asian species, while CG16_3_1300 is aligned with Sur and haplotype 1. Notably, the CG16_4_1050 band in Sur and haplotype 1 included additional sequences containing repetitive motifs compared to the Asian alleles (Figure S3). However, unlike *CG8*, this region did not include a conserved domain. In addition to the significant differences detected in *CG8* and *CG16*, the CDS alignment revealed other variable regions that could serve as novel EST‐SSR markers (Table 5 and Table S5), particularly for *CG10* and *CG25*, which exhibited lower conservation in *C. sativa*, as indicated by their pairwise identity below 90% in the CDS alignment conducted in our study.

**TABLE 5:** Expressed sequence tag‐derived simple sequence repeat (EST‐SSR) with variability among the available coding sequence (CDS).

Gene ID	CDS position	Sequence
*CG3*	281	(ACA)n
	297	(CGA)n
	430	(AAAGAG)n
*CG10*	1513	(AT)n
*CG13*	676	(AAC)n
	1723	(CAA)n
*CG15*	128	(AA)n
	1754	(GAA)n
*CG17*	617	(CAG)n
	628	(CAA)n
*CG24*	202	(GGA)n
*CG25*	1959	(GAGGAA)n
	2000	(AAG)n
	2411	(AGA)n
*CG26*	85	(ATC)n

## 4 DISCUSSION

Climate change is intensifying drought events, posing a significant threat to forest ecosystems and highlighting the urgent need to identify adaptive traits in tree species. In this study, we explored the genetic basis of drought tolerance in *C. sativa* by integrating comparative genomics with experimental validation. Our approach successfully reconstructed CDSs of candidate genes and identified genetic variability within *C. sativa*, proving a valuable foundation for future research on drought adaptation in this species. Using the transcriptome profile of genes conferring drought tolerance in oak species as a starting point, we employed a comparative genomics approach to pinpoint the homologous sequences that may underlie drought tolerance in *C. sativa*. Mapping short reads from *C. sativa* to candidate genes proved to be an effective strategy, supported by the high percentages of identity and coverage, both ∼97% (Table 3; Supporting information S1). Compared to our previous approach (Perez‐Rial et al., 2024), this updated method significantly improved the metrics; that is, mapping identity increased from 87% to 97%, mapping coverage from 80% to 97%, and the number of short‐read sequences mapped rose from 9.7 to 14.2. Leveraging annotated information from Asian chestnut to determine CDSs enhanced the accuracy of the annotation predictions, improving the overall reliability of gene prediction. This enabled more precise comparisons at the CDS level. The effectiveness of this approach was further confirmed by comparing short‐read mapping results with sequences generated by Sanger sequencing (Table S4). Additionally, comparisons with genome assembly data, when available, demonstrated a high degree of identity between the sequences obtained by using our method and the genome assembly (Table 4), highlighting the potential of this approach to be used by researchers dealing with limited genomic resources.

Our findings corroborate the highly conserved genomic architecture among species within the Fagaceae family, as previously reported (Staton et al., 2020), and extend this conservation to the CDS level. However, we identified certain candidate genes with lower conservation, suggesting their potential role in explaining variations in drought tolerance across and within species (Alcaide et al., 2022; Hamanishi et al., 2010; Madritsch et al., 2019). As these genes could be key players in adaptive responses to environmental stress, they require further investigation. Notable differences were identified in certain genes between the assembled haplotypes of the Italian *C. sativa* Marrone di Chiusa Pesio cultivar (Bianco et al., 2024), particularly *CG16*, a gene located within a 1.3 Mb region exclusive to haplotype 1 (Table 4; Figure 4). While this fact could stem from an assembly error of haplotype 2, significant variation was also observed when this gene was amplified in Iberian genotypes (e.g., Sur and Torito; Figure 3).

Despite the overall genome conservation in Fagaceae, the presence or absence of specific genes and differences in annotation should be expected. Genomic diversity within species often exceeds what a single reference genome can capture (Bayer et al., 2020; Hu et al., 2022). Early pangenome studies in plants showed that 15%–40% of the gene content varies between individuals, with gene presence or absence linked to stress tolerance and environmental adaptation. In *Populus*, recent pangenome research estimated that 0.63%–1.42% of genes were hemizygous (i.e., present in only one of the two homologous chromosomes) across 10 species (Shi et al., 2024). Similar patterns have been observed in Fagaceae, where haplotype‐resolved genome analyses reveal substantial variation (Larson et al., 2025; Luo et al., 2024). For instance, in *C. mollissima* Hongli‐1, over 600 annotated genes differ between haplotypes (Qiao et al., 2024).

We also detected two large consecutive inversions of 1.2 and 1.3 Mb on chromosome 5 of *C. sativa*, close to the exclusive region of haplotype 1 that harbors *CG16* (Figure 4). Large structural variants of this type are rare among Fagaceae species with haplotype‐resolved genomes. In *Quercus alba*, only two inversions larger than 1 Mb have been identified on chromosomes 3 and 9 (Larson et al., 2025), while in *C. mollissima* Hongli‐1, 47 inversions were detected, spanning a total of 2.31 Mb in haplotype A and 2.17 Mb in haplotype B (Qiao et al., 2024). Structural variations have been linked to phenotypic differences in woody plants such as *Theobroma cacao* (Hämälä et al., 2021) and *Prunus persica* (Guo et al., 2020), where a 1.67 Mb inversion was associated with fruit shape. Chromosomal inversions, by suppressing recombination, can function as supergenes that maintain favorable combinations of adaptive alleles, playing a key role in local adaptation and evolutionary divergence (Kollar et al., 2025). Additionally, GO enrichment analysis in the haplotype‐resolved genome of *Quercus glauca* (Luo et al., 2024) suggested that many structural variations are involved in responses to abiotic and biotic stimuli. Therefore, the two consecutive inversions detected on chromosome 5 represent a relevant structural variation that should be further investigated.

We detected large polymorphisms in *CG8* (Figure S1) and *CG16* (Figure S2), which may have significant implications on the structure and function of the resulting proteins. These polymorphisms were notable for their potential to be easily detected by agarose gel electrophoresis due to the extensive variation observed in the CDS of these genes. *CG8* encodes an oleosin 5‐like protein, which belongs to a family of small, structural hydrophobic proteins found on plant lipid droplets (Cao, 2015; Shao et al., 2019; Yuan et al., 2021). Oleosins have been shown to prevent the fusion of storage organelles during dehydration, thereby contributing to freezing tolerance (Dong et al., 2024; Shimada et al., 2018). Their functional role in response to abiotic stresses, including drought, suggests a complex and finely tuned regulation of stress signaling pathways within this gene family (Kim & Hyun, 2024; W. Zhang et al., 2023). Moreover, oleosins have been proposed to support membrane integrity and osmotic adjustment under water deficit conditions (Shao et al., 2019). Variability in the oleosin gene family is common among terrestrial plants, with species‐specific duplications often driving functional divergence, positive selection, and rapid evolution (Liu et al., 2012; Zou et al., 2022). Given the well‐documented dynamic roles of this gene family, the variability observed in *CG8* may reflect an adaptive mechanism linked to environmental stress, including drought tolerance.

*CG16* was located in a region absent in haplotype 2 of *C. sativa* Marrone di Chiusa Pesio cultivar (Figure 4). PCR amplification of this gene revealed notable heterozygosity in Sur, which had one allele similar to Asian chestnut species (CG16_3_700 and CG16_4_720) and another allele resembling haplotype 1 of *C. sativa* Marrone di Chiusa Pesio cultivar (CG16_3_1300 and CG16_4_1050). In Torito, *CG16* appeared to be homozygous for distinct alleles (a mixed pattern, CG16_3_1300 and CG16_4_720). *CG16* was identified as “two‐component response regulator ARR2‐like”, belonging to the type‐B plant response regulators (RRs, called ARRs in *Arabidopsis thaliana*), transcription factors that play a central role in activating cytokinin (CK)‐responsive genes that regulate growth, development, and stress responses (Argueso et al., 2010; Hai et al., 2020; Mason et al., 2005; Nguyen et al., 2016). In *A. thaliana*, several type‐B ARRs have been shown to negatively regulate drought responses, with loss‐of‐function mutants exhibiting enhanced tolerance (Nguyen et al., 2016). Cytokinin signaling is closely integrated with ABA pathways, contributing to drought adaptation through hormonal cross‐talk (Hai et al., 2020). It will be important for future studies to determine whether the variability observed in *CG16* affects cytokinin‐responsive regulation, potentially influencing the *C. sativa* response to water deficit. We also identified variability in other candidate genes that could serve as genetic markers in *C. sativa*. For instance, *CG10* and *CG25*, associated with the response to stress and stimulus (Table 2), exhibited a high level of CDS variability with less than 90% identity in *C. sativa* (Table 4). In addition, we detected single sequence repeats in two other candidate genes functionally annotated as transcription factors, *CG13* (“WRKY transcription factor WRKY24‐like”) and *CG17* (“transcription factor TCP9”). Both genes belong to families involved in response to stress and environmental changes, participating in multiple biological pathways (Danisman, 2016; Li et al., 2024; Wu et al., 2024; Z. Zhang et al., 2023). Their potential as molecular markers for stress tolerance should also be emphasized.

The sampling of two genotypes may constrain the conclusions regarding the broader genetic variability of the species. Nonetheless, Sur and Torito were deliberately selected based on their documented drought tolerance within the chestnut breeding program. Although the study focused on these two genotypes, we were still able to detect polymorphisms across several candidate genes, indicating the presence of intraspecific genetic variation potentially associated with drought adaptation. Ongoing research includes the resequencing of additional drought‐tolerant and susceptible genotypes, along with qPCR‐based gene expression analysis to assess whether the observed variability correlates with differential expression under drought conditions. These efforts aim to clarify the functional significance of the identified candidate genes and evaluate their potential as molecular markers. Such markers could support the selection and deployment of drought‐tolerant genotypes adapted to Mediterranean environments, where climate change is expected to exacerbate water scarcity.

## 5 CONCLUSION

The methodology used in this study successfully identified candidate genes for drought tolerance in *C. sativa*, providing valuable insights into the genetic basis of adaptive responses to environmental stress. Despite the high genetic conservation observed within Fagaceae, particularly in *Castanea*, the analysis of sequences obtained from different approaches and databases has revealed notable differences with potential application as genetic markers for screening chestnut trees for drought tolerance. In particular, *CG8*, an oleosin‐like protein, and *CG16*, a two‐component response regulator, showed significant differences in the CDS, which is of interest for further study as they may play a critical role in adaptive responses to environmental stress.

Overall, our approach—including short‐read mapping coupled with Sanger sequencing—has proven effective in reconstructing CDSs and detecting gene variability in *C. sativa*. The high level of identity observed between the methodology we followed and the sequences obtained from the genome assembly opens prospects for researchers with limited genomic data. Our approach not only highlighted genes potentially associated with drought tolerance, but also established a reliable framework for studying genetic diversity and developing markers in plant species lacking complete genome references.

Given the increasing challenges climate change poses to forest ecosystems, identifying adaptive traits in tree species is more urgent than ever. The recognition of forestry's role in climate strategies underscores the importance of integrating genetic approaches into conservation and breeding programs. By providing genetic insights into drought tolerance, our study contributes to the ongoing efforts aimed at enhancing the resilience of *C. sativa* under changing environmental conditions.

## AUTHOR CONTRIBUTIONS

**A. Perez‐Rial**: Formal analysis; investigation; methodology; writing—original draft. **P. Castro**: Conceptualization; methodology; resources; writing—review and editing. **A. Solla**: Resources; writing—review and editing. **M. A. Martín**: Conceptualization; methodology; resources; supervision; writing—review and editing. **J. V. Die**: Conceptualization; funding acquisition; methodology; resources; supervision; writing—review and editing.

## CONFLICT OF INTEREST STATEMENT

The authors declare no conflicts of interest.

## Supporting information




**Figure S1**. Alignment of *in silico C. sativa* gene sequences with those of the Asian species C*. mollissima* and *C. crenata*, illustrating primer design strategy.

**Figure S2**. Alignment of *CG8* between *C. sativa* haplotype 1, *C. sativa* haplotype 2, and the Asian species *C. crenata* and *C. mollissima*.

**Figure S3**. Alignment of the theoretical CG16_4 amplicons between *C. sativa* haplotype 1 and the Asian species *C. crenata* and *C. mollissima*.

**Table S1**. Functional annotation of the initial gene data list selected from Madritsch et al. (2019).
**Table S2**. Designed primers for CDS Sanger sequencing.
**Table S3**. Features of CDS sequenced using Sanger sequencing.
**Table S4**. BLASTn of in silico CDS obtained by short‐read mapping against CDS obtained by Sanger Sequencing.
**Table S5**. Simple sequence repeats within the CDS of the candidate genes.

**Data S1**. Atlas of in silico putative CDS candidate genes for drought tolerance in *C. sativa*.

**Data S2**. CDS of candidate genes for drought tolerance in *C. sativa* sequenced using Sanger methodology.

## Data Availability

The sequences generated in this study are provided as FASTA files in the Supporting Information. The Sanger‐sequenced genes have been submitted to the GenBank database and are accessible under the accession numbers PQ766573 – PQ766583.
