# Supplementary material for: Decoding drought tolerance from a genomic approach in Castanea sativa Mill
Source: Plant Genome. 2025 Nov 9;18(4):e70116. doi: 10.1002/tpg2.70116 (PMC12598267; doi:10.1002/tpg2.70116)
Supplement: Supplementary file 1 [file TPG2-18-e70116-s002.pdf]

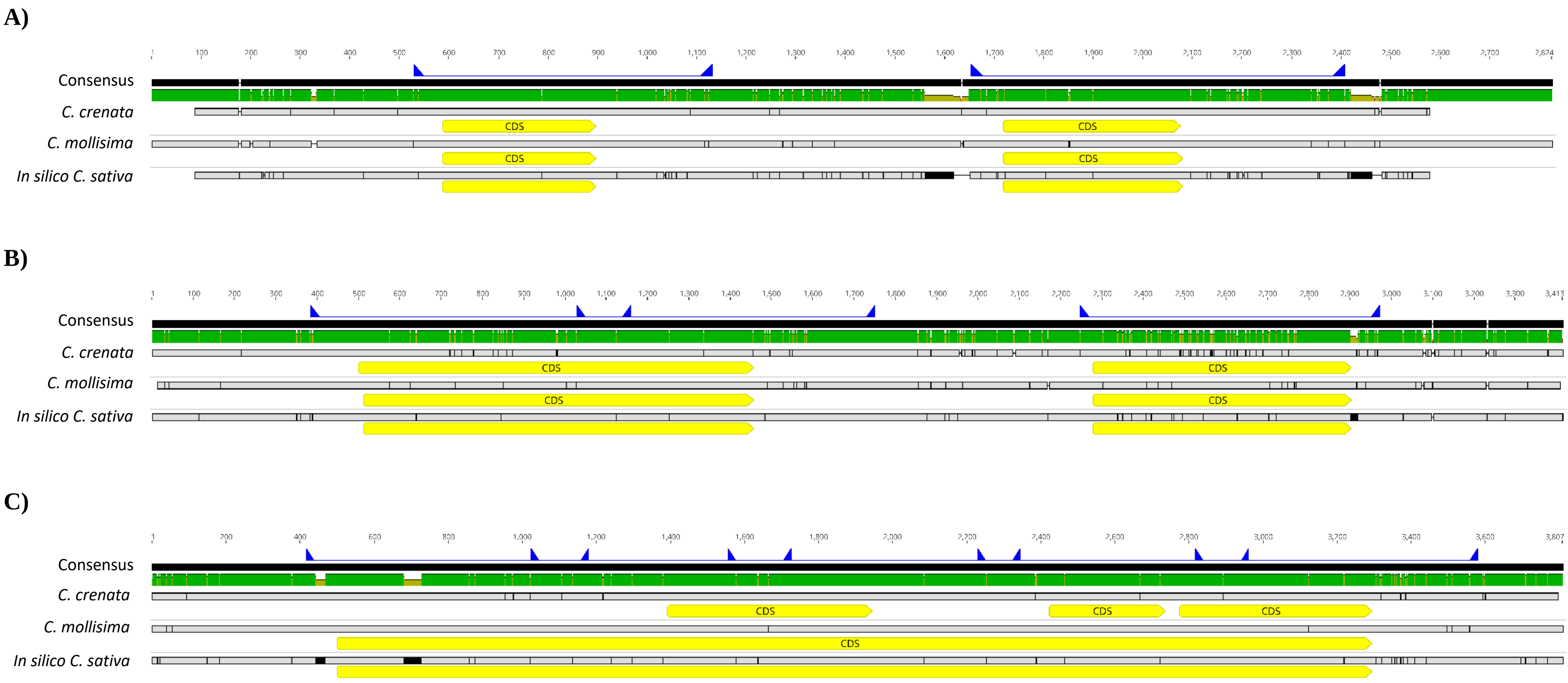

Figure S1. Alignment of *in silico C. sativa* gene sequences with those of the Asian species *C. mollissima* and *C. crenata*, illustrating three primer design strategies: **A)** *CG18*, an example with a single primer pair covering one CDS; **B)** *CG4*, an example with two primer pairs amplifying a single CDS in overlapping fragments; and **C)** *CG2*, an example showing discrepancies in CDS annotation between *C. mollissima* and *C. crenata*, where primers were designed based on the longest annotated CDS regions. Gene sequences are represented by gray bars, with black segments indicating regions of lower identity. Lines connecting the bars indicate alignment gaps. Sequence identity is shown above the alignment, with green indicating high identity and yellow indicating lower identity. Annotated CDS regions appear in yellow below each gene, and primer pairs are shown as blue triangles.
