# Supplementary material for: Decoding drought tolerance from a genomic approach in Castanea sativa Mill
Source: Plant Genome. 2025 Nov 9;18(4):e70116. doi: 10.1002/tpg2.70116 (PMC12598267; doi:10.1002/tpg2.70116)
Supplement: Supplementary file 2 [file TPG2-18-e70116-s003.pdf]

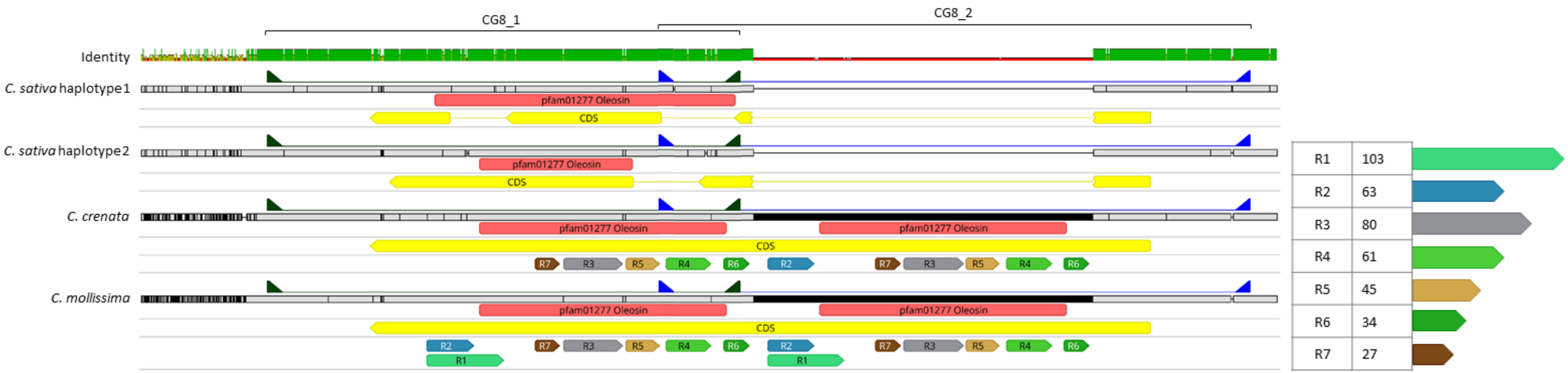

Figure S2. Alignment of *CG8* between *C. sativa* haplotype 1, *C. sativa* haplotype 2, and the Asian species *C. crenata* and *C. mollissima*. Gray bars represent the gene sequences for each species, with black segments indicating regions of lower identity. Lines connecting the bars represent alignment gaps, where no sequence is present. Sequence identity is shown above the alignment: green denotes high identity, while red indicates low identity. The annotated coding sequence (CDS) is displayed in yellow below each gene sequence. Primer pairs designed to amplify the CDS are shown as green (CG8_1) and blue (CG8_2) triangles, with their names indicated at the top of the figure. The region amplified by primer CG8_2 reveals a deletion in *C. sativa*, corresponding to an oleosin domain (pfam01277) present in the Asian species. Repetitive elements (R) within this region are represented as numbered arrows, and their size and color correspondence are indicated in the figure legend.
