# Supplementary material for: Decoding drought tolerance from a genomic approach in Castanea sativa Mill
Source: Plant Genome. 2025 Nov 9;18(4):e70116. doi: 10.1002/tpg2.70116 (PMC12598267; doi:10.1002/tpg2.70116)
Supplement: Supplementary file 3 — Figure S3. Alignment of the theoretical CG16_4 amplicons between C. sativa haplotype 1 and the Asian species C. crenata and C. mollissima. [file TPG2-18-e70116-s005.pdf]

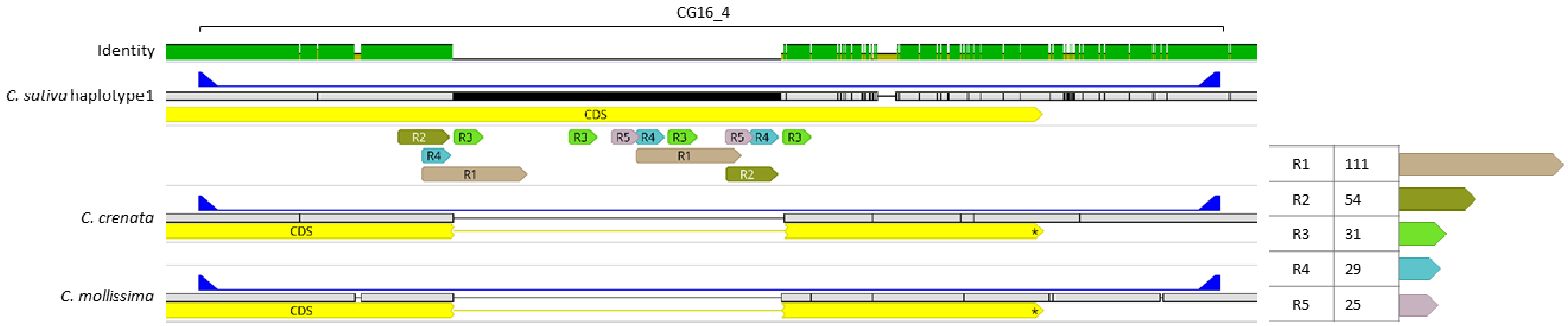

Figure S3. Alignment of the theoretical CG16_4 amplicons between *C. sativa* haplotype 1 and the Asian species *C. crenata* and *C. mollissima*. Primer pairs are shown as blue triangles. Gray bars represent the gene sequences for each species, with black segments indicating regions of lower identity. Lines connecting the bars represent alignment gaps, where no sequence is present. Sequence identity is shown above the alignment: green denotes high identity, while red indicates low identity. The annotated coding sequence (CDS) is displayed in yellow below each gene sequence. The region amplified by this primer reveals an insertion in *C. sativa* that includes repetitive elements (R), represented as numbered arrows; their size and color correspondence are indicated in the figure legend. Notably, this region does not contain any conserved domain.
