# Supplementary material for: Decoding drought tolerance from a genomic approach in Castanea sativa Mill
Source: Plant Genome. 2025 Nov 9;18(4):e70116. doi: 10.1002/tpg2.70116 (PMC12598267; doi:10.1002/tpg2.70116)
Supplement: Supplementary file 5 — Data S1. Atlas of in silico putative CDS candidate genes for drought tolerance in C. sativa. [file TPG2-18-e70116-s006.docx]

>CG1 [organism=Castanea sativa], in silico CDS obtained by short-read mapping

ATGGATGTTGAAGCAGTTGTCTCTATAGTGATACGGAAACTCACAGACCTGCTCATTCAAGAACCAATCATCTTCAACAA

GGCTATAGATGAGATAGAGCAAGTCAGAATCAGCTTAAGACAGATGCAGAGCTTCCTAATAGATGCAGAAGATAAAAAAG

AACACGAAGAAGGTGTTAAGNNNNNNNNNNNNNNNNNNNNNNNNNNNNNNNNNNNNNNNNNNNNNNNNATTGAAACGTTT

GTCCTGTGGAAAATGTATGCAAGGAGAATGGGGTACTTTTTCATCCCCAAGAATTTGAAGGCTGACTCTGACCTTCGCAA

TAAGATAGAGGAGATCAAGCACAAGATAAAAAACTTTGACAAAATGGAGACAGGTGGGGTCTCAAACCGAGGCGAGCCTC

TCATGATTCGGCAATTAACTTTGCACAGGTCAAGTTATCGAGATGAAGGGCTAACACTGAATCAGCAAAACAATGATTCC

AGCTATTCTGTGGATGAAGAAACAGACTTCATTGGCTTCGAGAAAGACAAAAGCAAGCTGGTGGCCATGCTAACTGGCAG

TGGCCCGGAATCCGGGGATTACCAAAAGTTTCCTGCCATTTCAGTTGTAGGCAAGCATGGCACTGGCAAGACTATCCTTG

CAAAGGCAATTGAGGTTAAAAGTCGTCTTGATCGTCGCGTCATTTCAGTTGTAGGCAAGCATGGCTCTGGCAAGACTACC

CTTGCTAAGGCAATTTATAAAAGTCCTGAGGTTAAAAGTGATTTTGATTGTCGCGCATGGGTTCTTGCCTCTGGGAACCT

TACAGATGTGCTGTTGAGCATATTGGAACAGATTGTCAATATAACTGTTGATAATAAATCTACGAAAGAAGAATTGACAC

AGAAGATCCATATGAATTTGCAGGAGAAACGGTACCTGGTGGTGTTGGACGATTTACAGACGCCTAGTTTATGGAAAGAA

CTTCTTGAGGCCTTTCCAGACACAAATAAAGGTAGCAGGATCATATTAACTACCATCAATTATCGCGTCGCTTTTTCAGC

AGATACAAGAGGAGAGCCTCACCGGCTCAATCCTCTGAATGAGAAAGACACCTGGAAATTATTCTTGAAAAAGGTACGCT

TACCAGACAACTTRATGTTCTCTGATGAAGAAGTCTTCAAGCTTAAAAGGAAACTTATAAGAAGGTGTAAGGGCTTACCT

CTGGCAATCGTGGTGCTTGGAGGTTTGCTGTCGACGAAGGATCCGAACTACGAAGTATGGTTGAAAGTTTTTGAGCACCC

AAGTTGGCAGCTAGACAGAAAGCAAGACCAGTTCTCAATTATATTGGCCCTGAGTTATAATGATCTGCCCTTCCACTTGA

GGCCTTGCTTCCTCTATTTACTGCTTTTCCCAAAAGATAAAGATATTCCTGTGAGAAGGTTGCAGCGGCTATGGCTTGCA

GAGGGATTTGTGAAGCATACACCCGAGAAGACTCCGGAAGATATGGTGGAGATATACTTCAAAGAACTTGTGCAAAGAAA

CTTGATTCAAATATTCAAATGGAGAAAAGACGGGAGTCCCAAAACATGTCGTATTGGTGTCTTACAAGATGACGGTAATT

TCATGTCAAAAGCTCAAGAGATTGGCCTTTTCCACATCCAAAAAATGTCAGAAAAGGAAACTCCTCAGCATGGTGTTCGC

CGGGTTACTGACTTTGTTGACCCCGAAGAGTACATCTCACAAATTCAAAATCTGCGCTCTTACTTGGCCTTYGACATTCA

AAAAAATGATATGCCTGCGACAGAAATTGATAGGTTTCTCAACAAAGTCACTGGTCAAAATTTTGGATTGCTGAGGGTGG

TTGATCTAGAGCGTGTTTACAAGCCTAAACTGCCCGACAATATGGGKAAACTGTTTCTCTTGTTGAGGTACTTGGGTTTA

AGATGGACCTTTTTGGATGCCCTTCCCCACTCTGTTGGTGAGMTGCCTTACCTTGAGACTTTGGATGTGAAGCACACATA

TATCAGCTCTCTACCGAGTTCTATTTGGAAGATGAAACACCTGCGCCATCTGTGTCTGAATGAAATACGTCTTGACATGT

CTCTGCAAAACCATGGCACTTCTCTTACTCATCTCCAGACATTATGGGGATTATTCGTAGACAAAAAGACTCCAGTGAAG

AATGGTTTGTATAGGTTAATCAATCTCAGGAAATTGGGTCTGACATGTCATTTAGATTCCTTTCAAGAATTAGATGAGTG

GATTGCAAGACTTGCAAGTCTTCAATCTCTCAGGATACGATCCAAGAACCAAAACGGTCAACCTTGGAAGCTCAATTTGA

AGCCTCTGTCAAGCCTTGAGAATCTCACCAACCTGTATTTGCTTGGAAGCTTACCGGAGCTACATGATAGGTATGAATTC

CCTCCTAAACTTACAGTTCTTACTTTGTCGGTCTCAAAGCTGGAGAAAGACCCCATGCCAATCCTAGCGCAGCTGCCAAG

CCTCTCTGTCCTCAGGCTATTGGCAGATTCCTACACTGGGAAGGAATTGAAGTGCCCACCCAAAGGGTTCATTAAGCTTC

AAATTTTGAAGCTTTGGATGCTAAAGGATTTGGAGACATGGGAGGTGGGGAAAGAAGCACTTAAGGAACTCCAAGAAGTA

GAAATCCGATGCTGTGACAAGCTAAAGGAACTTCCAGCCCCATTGTTTATTTTGGAAAACATTAAGAAAATAGTYTTRAC

GAACATGCCACAGAAATTTGTAGATGAGATTCCAGCCAAAGGATTAATCTTCCCCAAAACTCTGCAGTTCTGA

>CG2 [organism=Castanea sativa], in silico CDS obtained by short-read mapping

ATGGAAATTACCTCTCATCTCCTTGCAATTGCAGGGGCTTTTGTACTGGTACTATTGTACAATCTATGGAGGCTGAGAAT

TGGTAGTCACAAAAGTAAGGGTATGTTGGCCCCGGAGCCATCAGGTGCGTTGCCAATCATAGGTCACCTTCACAAACTAG

GTGGCCAAAACCCAATTGCCCGAACCTTGGCAGCTATGGCTGATCAATATGGTCCCATATTCACGATCCGATTTGGTATG

AAAAGTGCAATTGTTATTAGCAATCATGAGGCTGTCAAGGATTGTTTCACTACAAATGACAAGGCTTTGGCTGCACGTCC

AAGGTCTAGCCATGGAAAGTACCTTGGCTACAACTATGCAGGATTTGGGTTCATCAACTATGGAAAATTCTGGCTCAAGA

TGAGAAAGATAACCATGCTAGAACTCCTCTCTAGTCGTCGGCTTGAGACACTAAAGAATGTGCAAGATACTGAGGTCGAC

AATTTGATTAAAGATTTGTACACTCTTTGCATGAGCAAAGAGCACAACAACCAAGCTAAGGTGGTGATTAGTGAGTGGAT

TGAGCGTTTAACCTTCAATATAATTACTAAAATGATTGCAGGGAAAAGATACGTTGGAAACTTGAATGATGGGAATGACG

GAGAGGCACAAAGAATAGGGAAAATTATTAAAGAGTTCATGTATGTATCTGGGGTCCCTGTTGTTTCGGATCTTGTTCCA

TTTCTAGGAGGGCTTGAATATTTTCTGGGCCAAGTAAAATCCATGAAGCGAATTGCAAGGGAATTGGACACTCTAGTTGG

AAGTTGGGTTGAAGAACATGCTATGAGGAGGGCGGAAAGTGAACGAATTGACAAACCGGACTTTATTGATATCATGTTAT

CTGTAATTGAGGATGACGGCATGTTTGGCCATACGCGTGAAACCATTATCAAGGCAACAGCATTGAATCTCATCTTGGCT

GGGTCCGACACTACATCTCTTAACTTGACATGGCTCTTGTCYATATTATTGAAYAACAAGCACGCTTTGAAGCAAGCYCA

AGAAGAGCTCGATCTCAAGGTTGGTAGAGASAGRTGGGTGGACRAYCATGATATCAAAGAYCTAGTTTACCTCCAAGCCA

TAGTGAAGGAAACMTTGCRCTTGTACCCACCAAGCCCCCTATCAGTTCCACATGAGGCCATGGAGGATTGTCATGTTTGT

GGCTATTAYGTCCCAAAGGGTACTCGTTTGCTTGTTAATGTGTGGAAGTTGCACCGAGATCCAAGAATTTGGGAGGACCC

AGAAAAGTTTYTACCAGAGAGGTTCCTCACAAGCCATGCAAGTATAGATGCTTCAGGTCAACATTTTGAGTTCATACCAT

TTGGGTCTRGTAGACGATCTTGCCCRGGTTACACATTTGCMTTGCAAGTATCTCACTTGACACTAGCTCGATTACTTCAG

GGATTTGAGTTCACGACACCATTGAATATGCCAATAGACATGACTGAAGGCTTGGGCATTACCTTACCCAAGGCAACTCC

CCTTGAAGTTCTTCTCAATCCACGCCTTGCTCCGGAACTCTATCAANNN

>CG4 [organism=Castanea sativa], in silico CDS obtained by short-read mapping

ATGGCGTCGGAGACTGAAACGTTCGCATTCCAAGCCGAGATAAACCAGCTTCTCAGCTTGATCATCAACACCTTCTACTC

CAACAAGGAGATCTTTCTTCGTGAGCTTATCAGCAACGCTTCCGATGCTCTGGACAAAATCCGTTTTGATAGCTTGACTG

ACAAGAGTAAGCTCGAGGCGCAGCCTGAGCTTTTCATTCATATTATCCCTGACAAGGCCAGCAATACTATCACCATCGTT

GACAGTGGGATTGGAATGACCAAGGCTGACCTGGTAAACAATCTTGGAACCATTGCAAGATCTGGAACCAAGGAGTTTAT

GGAACACTTGGCTGCTGGTGCGGATGTTAGCATGATTGGTCAGTTTGGAGTTGGGTTTTACTCTGCTTACTTGGTTGCTG

AGAGGGTGGTTGTTACCGCAAAGCACAATGATGACGAACAGTATATCTGGGAGTCTCAGGCTGGTGGTTCTTTCACTGTA

ACTAGGGACACTTCTGGTGAGAACCTTGGAAGGGGTACTAAGATCACCCTTTTCCTCAAGGAAGATCAATTGGAATACCT

TGAGGAGCGTCGTCTTAAGGACCTTGTCAAGAAGCATTCTGAGTTCATAAGTTACCCAATTTCCCTTTGGGTGGAGAAGA

CTGTAGAGAAGGARATTTCAGATGATGAAGATGAAGAGGATAAGAAGGAGGAGGAAAAGAAGGAGGAGGAGGAAGGTAAG

GTGGAAGATGTTGATGAAGAAGAAAAAGACAAGGAAGAGAAGAAAAAGAAGAAGATCAAGGAGGTGTCCCATGAGTGGTC

ATTGGTCAATAAGCAGAAGCCCGTCTGGATGAGGAAGCCAGAAGAGATCACTAAGGAGGAGTACTCTGCATTTTACAAGA

GTCTCACCAATGACTGGGAGGAGCACCTTGCTGTGAAGCATTTCTCAGTTGAGGGTCAGCTTGAATTCAAGGCTATCCTC

TTTGTTCCCAAGAGGGCACCTTTTGACCTATTTGACACAAAGAAGAAGCCCAACAACATCAAGCTCTATGTCCGCCGTGT

CTTTATTATGGATAACTGTGAGGAATTGATTCCTGAATATTTGGGTTTTGTGAAGGGTATTGTGGATTCTGAGGATCTGC

CTCTCAACATTTCAAGAGAGATGCTGCAGCAAAACAAGATCCTCAAGGTTATCCGGAAAAATCTTGTCAAGAAATGTATT

GAGCTCTTCTTTGAGATTGCTGAGAACAAGGAGGACTACAACAAGTTCTACGAGGCATTCTCAAAGAATTTGAAGCTTGG

TATTCATGAGGATTCACAGAACAAAACCAAAATTGCCGAATTGCTTCGGTATCACTCCACCAAAAGTGGAGAGGAGATGA

CTAGCCTGAAGGATTACGTCACAAGGATGAAGGAGGGGCAGAGTGATATCTACTACATTACTGGAGAGAGCAAAAAGGCT

GTAGAGAATTCCCCATTCCTTGAGAAGCTGAAGAAAAAGGGATATGAGGTTTTGTTTATGGTTGATGCTATTGATGAGTA

CGCCGTGGGTCAGTTGAAGGAGTTTGAGGGGAAGAAGCTTGTCTCTGCAACCAAGGAGGGACTCAAGCTTGAGGAGAGTG

AAGATGAGAAGACTAAGAAAGAAGCATTGGCTGAGAAATTTGAGGGACTTTGCAAGGTGATGAAAGATGTGCTTGGTGAC

AAGGTTGARAAGGTGGTGGTATCTGATCGTGTGGTGGATTCCCCCTGCTGCTTGGTAACTGGAGAGTATGGATGGACAGC

AAACATGGAGAGAATTATGAAGGCTCAAGCACTGAGGGATTCAAGTATGGCTGGTTACATGTCTAGCAAGAAGACCATGG

AGATTAATCCAGAGAACCCCATCATGGAGGAATTGAGGAAGAGGGCTGAGGCAGACAAGAATGACAAGTCAGTGAAGGAC

TTGGTTCTATTGTTGTTTGAGACCTCTCTTCTTACATCTGGCTTCAGCTTGGATGAGCCCAATACTTTTGGCAACCGAAT

TCACAGAATGCTTAAATTGGGATTGAGCATTGATGAAGACACTGCTGAAGCTGACACTGAGATGCCTGCTCTAGAGGAGG

CCGATGCTGACGCAGAGGGGAGCAAGATGGAGGAGGTCGACTNN

>CG6 [organism=Castanea sativa], in silico CDS obtained by short-read mapping

ATGGCAGGCTCTATGACCATTTGTTTCTTTGTGGTGCTTTCGATCTCTATTTTGTTATCTTGGACCAACCCAAGAGGCGA

AACCCTAAAATCCTTCTCTCTCTCTCCTCTCTATTCTTCTACACAGTCGGCTTCTTCTTCCAATGAGGATATCTCCGGCT

TGAGGTACGATTTCTACCATGAAACTTGTCCACAGGCAGAGTCCATTGTAAGGACTCACATGGCTCGCTTATATTCCGAC

CATAACAATGTCTCTGCAGCTCTGCTGCGTCTTTTCTTCCATGACTGCTTCATTGAGGGCTGTGATGCTTCTGTCCTCTT

GGRTGACAGYAATGGTGACAAAAACCATTCCATMGAGAGGCAGGCCATACCCAATCAGACCNNNNNNNNNTTTGATAAAA

TYGATTCCATCAAGGAGGAGCTTGAAAASGCTTGTCCAGGGGTAGTTTCATGTGCTGATATTGTTTCTATTGCCACTAGA

GATGGCATTATGCTGGCTGGTGGCCCTTTCTATCCAGTKTTAACAGGCCGGAGGGACAGCATTCATTCATATTTCAATGA

AGCACTGGCCGCGATTCCAYGACCCGATGATAACATAACACAGACMCTTCACCTTTTTGACCTTAGAGGTTTTGATGAAA

GAGARACWGTCAGCCTTCTAGGYGCRCACAACATTGGGAAGATTGGCTGTGAATTCAYACAGAAACGTCTSTCTAACTTC

AAGGGGACAKGGCAGCCAGACCCAACTATAGCTCCTGATTTCCTAACTGAGATGAGAATGAGGTGCCAAGACAGTAATRG

GACCACCACTCGRCCYTCTTCTTCATCCATGGCATCATTGGAAATGAGTGAGTCAGCAGTGGGGATGTCATATTTGCAGG

CATTKTCATCTTCGATWTCATCTGGGGCAGGTTTTGATACTCACTACTATCAGAGCYTGTTAARTGGGAGAGGACTCCTT

TTTGCYGATCAGCAATTRATGGCTAATGAGAAGACTGCGAGACTGGTRAGGGCTTATGCTTCAGATGAYGGATCAACCTT

TCGAATGGACTTTGCAKGGGCAATGATGAAGATGTCAGGCCTTAAYRTTCTGACTGGATCTCAAGGTCAGGTCCGACGGA

AYTGCTCCTTGCCTTTGGTCAGTTCCTAA

>CG8 [organism=Castanea sativa], in silico CDS obtained by short-read mapping

ATGGCTGAGATCCACCAGCCACATCACTATCAACCACAACAGCAACACCACCAACGCCCCGGTNNNNNNNNNNNNNNNNN

NNNNNNNNCAAAAGTACTANCNGTTGTGACTCTNNNNNNTCATAGGCCTAGCTGTTTCAACCCCACTGCTTGTGATTTTC

AGCCCTGTTCTTGTCCCAGCAGCAATAGTCATTGGCTTGGCCGTGGCTGGATTTTTAACCTCAGGAGCTTTTGGAATCAC

AGGGCTTTCTTCACTGTNNNNNNNNNNNNNNNNNNNNNNNNNNNNNNNNNNNNNNNNNNNNNNNNNNNNNNNNNNNNNNN

NNNNNNNNNNNNNNNNNNNNNNNNNNNNNNNNNNNNNNNNNNNNNNNNNNNNNNNNNNNNNNNNNNNNNNNNNNNNNNNN

NNNNNNNNNNNNNNNNNNNNNNNNNNNNNNNNNNNNNNNNNNNNNNNNNNNNNNNNNGGCATGTTCTCTGAAAATAATCA

AGAGGGTCCTTCAGCTTCAAAAGTACTAGCAGTTGTGACTCTCTTACCCATTGGTGGCTTTCTTCTCATCTTTGCTGGTC

TCACATTGTTGGGACACTCATAGGCCTAGCTGTTTCAACCCCACTGCTTGTGATTTTCAGCCCTGTTCTTGTCCCAGCAG

CAATAGTCATTGGCTTGGCCGTGGCTGGATTTTTAACCTCAGGAGCTTTTGGAATCACAGGGCTTTCTTCACTGTCTTGT

ATAGCTAACTATCTGCGTCAAACAAGGGTGCCGGAGCACATGGATTATGCAAAGCGGCTGGTGCAGGACACGGTTGGTTA

TTATAATTAGAGAGCTAGGGAGATGGGCCACAATGTGAAGGAAAAAGCACAGGAAACTGGAAGATAGGTTACAGCTAGAA

CCCAAGAAGGCAGTGGAAGGACTCAAGAGGAGCGTAGGGGTACTGAAGTGGAGAAGTTGGAGGCNNNNGAAAGAGAGACC

TTTATGGGCTACTGA

>CG11 [organism=Castanea sativa], in silico CDS obtained by short-read mapping

ATGGCGGATACTGAGACGTTTGCTTTCCAGGCTGAGATCAACCAGTTGTTGAGTTTGATCATCAACACTTTCTACAGCAA

CAAGGAGATCTTTCTTCGTGAGCTTATCAGCAATGCCTCTGATGCTCTTGACAAGATTCGATTTGAGAGCTTGACCGACA

AGAGCAAGCTAGATGGTCAACCAGAGCTTTTCATCCACATCATTCCTGACAAGACCAACAACACCCTGACCATTATTGAC

AGTGGTATTGGAATGACTAAAGCTGATTTGGTGAACAACTTGGGTACCATTGCTAGGTCTGGAACCAAAGAGTTCATGGA

AGCCCTTGCAGCTGGGGCTGATGTGAGCATGATTGGTCAGTTTGGTGTTGGTTTCTACTCAGCGTACCTTGTTGCTGAGA

AGGTTATTGTCACCACAAAGCACAATGATGATGAGCAGTATGTGTGGGAGTCACAAGCTGGTGGGTCATTCACTGTTACC

AGAGACAACTCTGGTGAGGTCCTTGGTAGAGGTACCAAGATTACTCTCTACCTCAAGGARGACCAGCTTGAATACCTTGA

GGAGCGCCGCTTGAAGGATTTGATCAAGAAGCACTCYGAGTTTATCAGCTACCCAATTTCCCTTTGGATTGAGAAGACCA

CTGAGAAGGAGATTTCTGATGATGAGGATGAGGAAGAGAAAAAGGATGAGGAAGAGAAGAAGGACGAGGAGGGTAAGGTT

GAGGAGATCGACGAAGAGAAGGAGAAGGAAGAAAAAAAGAAGAAGAAGATCAAGGAGGTGTCCCATGAGTGGTCTTTGGT

TAACAAACAAAAGCCCATCTGGATGAGGAAGCCTGAGGAGATCGCAAAGGAAGAGTATGCTGCTTTCTATAAGAGCCTTA

CAAATGACTGGGAAGAGCACTTGGCTGTGAAGCACTTCTCAGTTGAGGGTCAGCTTGAGTTCAAGGCTATCCTCTTTGCA

CCAAAGAGGGCACCTTTTGATCTTTTTGACACAAGGAAGAAGCCAAACAATATTAAGCTCTATGTTCGTCGTGTATTTAT

CATGGACAACTGTGAGGAGCTGATCCCTGAATACCTTGGATTTGTTAAGGGTATTGTCGATTCTGAGGATCTTCCTCTCA

ACATTTCAAGAGAGACCCTTCAGCAGAACAAGATCCTTAAGGTCATCCGCAAGAATTTGGTTAAGAAGTGCATTGAGCTC

TTCTTTGAGATTGCTGAGAACAAGGAAGACTAYGCTAARTTCTATGAGGCTTTCTCTAAGAACCTCAAGCTTGGTATCCA

YGAGGATTCCACAAACAAAACAAAGCTTGCYGAGTTGCTCCGCTACCACTCCACCAAGAGCGGTGAAGAGATGACCAGCT

TGAAGGACTATGTGACCAGGATGAAGGAGGGCCAGAGTGACATCTATTACATCACTGGTGAGAGCAAGAAGGCTGTTGAG

AACTCTCCATTCCTTGAGAAGCTGAAGAAGAAGGGGTATGAGGTTCTGTACATGGTTGATGCTATTGATGAGTATGCTGT

TGGTCAGCTTAAGGAATTCGAGGGCAAGAAGCTTGTTTCTGCTACCAAGGAAGGTTTGAAACTTGATGAGAGTGAAGATG

AGAAGAAGAGACAGGAAGAGTTGAAGGAGAAGTTTGAGGGTCTCTGCAAGGTGATCAAGGATGTTTTGGGTGACAAGGTT

GAGAAAGTTGTGGTCTCTGACCGTGTTGTCGATTCTCCCTGCTGTTTAGTGACTGGTGAGTATGGATGGACTGCCAACAT

GGAAAGAATCATGAAGGCACAGGCTTTGAGGGACAGCAGCATGGCTGGCTACATGTCAAGCAAGAAGACCATGGAGATCA

ACCCTGAGAACTCTATCATGGAGGAGCTCAGGAAGAGGGCTGATGCTGACAAGAATGACAAATCAGTGAAGGACCTTGTT

CTCCTGCTTTTCGAGACTGCCCTCCTCACCTCAGGCTTCAGCCTTGACGAGCCCAACACCTTTGGCAACAGGATCCACAG

GATGCTGAAACTCGGTTTGAGCATTGATGAGGAAGCAGCTGATGGTGATGCTGAGATGCCCCCATTGGAGGAAGCTGATG

CTGATGCTGAGGGCAGCAAGATGGAGGAAGTTGATTAA

>CG13 [organism=Castanea sativa], in silico CDS obtained by short-read mapping

ATGGCTTCCTCTTCTGGGAGCTTGGATACCTCAGCTAACTCACACCCGGCTAGCTTCACTTTCTCTACTCACCCTTTCAT

GACAACCTCTTTCTCTGACCTCCTTGCCTCACCCAACGATATTGATGACCAAAACACCACATTAGAGAATAGAAATCGTA

GCTTGTCAGATCGTATAGCYGAGAGAACTGGGTCGGGTGTGCCTAAATTCAAGTCAATCCCACCTCCTTCTTTGCCCATT

TCTCCACCTGCTGTGTCTCCAAGCTCTTACTTTGCTATCCCAGCTGGGTTGAGCCCAGCTGAGCTCTTGGACTCCCCTGT

TCTTCTCAACGCTTCTAACATTCTGCCATCTCCAACAACAGGAACATTCCCTGCTCAGGCCTTCAATTGGAGGAGTAATT

TTAGCAACCAGCAGCAGAATGTTAAACAAGAAAACAAAAACTACTCAGATTTCTCTTTCCAAACTCAACAAAGGCCTCCT

TTATCATCAACAACAAACTATCAGCCTTCAAATACTGCGATTCAAACTGTACAGCCACAAGCCTGGAGTTTCCAAGAACC

CACCAAGCAGGATGATTTTTCCACAGGAAAGAGTATGGTAAAGGCTGAATTCGGTTCAATGCAGGGCTATTCCTCTGATA

TCACCACCATAACAAGTAACATTCAAAGCAACAATAACAACAACAACAACAACAGCAACAACGGTGGGCTCCAATCAGAA

TATGGTAATTATCACCAACAGCCTCAACAGCCTCAAACACTGAGTAGGAGGTCTGATGATGGGTACAATTGGAGGAAATA

TGGGCAAAAACAAGTTAAAGGAAGTGAAAATCCTAGAAGTTATTACAAGTGCACATACCCCAATTGCCCAACAAAGAAGA

AAGTTGAGAGGTCCTTAGATGGGCAAATTACTGAGATAGTTTACAAGGGTAGTCATAACCATCCCAAGCCTCAGTCTACT

AGGAGATCATCCTCAGCTTCTTCTCATGCCATTCAAGTTTCTAATTCTTCCTCCCATGAACTTCATGATCAGTCATATGC

CGCCCTTGGTAATGGACATATGGATTCGGTTGCAACCCCAGAAAACTCTTCCATATCGATGGGGGATGAGGATTTTGATC

AGAGTTCTCAAAAGAGCAAGTCCGCAGGAGATGACTATGATGAGGATGAGCCCGATGCCAAAAGATGGAAAAAAGAGGGT

GAAAATGAAGGTATATCAGCAGCCGGGAGCAGAACAGTGAGGGAACCTAGAGTTGTAGTTCAGACAACTAGTGATATAGA

TATTTTAGATGATGGATATCGTTGGAGGAAATATGGGCAGAAAGTGGTCAAGGGCAATCCAAACCCAAGGAGCTACTACA

AGTGCACATATCCAGGGTGTCCAGTGAGAAAGCATGTTGAGCGAGCATCTCATGATCTAAGGGCAGTGATCACAACCTAT

GAGGGGAAGCACAACCATGATGTTCCTGCAGCCCGTGGCAGTGGCAGCCATTCTGTCAATAGGCCTTTGCCAAGCAACAA

TAATAACAACATTAGTGCTGCCACAGCAATTAGGCCTTCAGCCATTACCCATCACACTAACAATTCTGTGAACAACCACC

ATCTTCACAATCTAAGACTACCAACATCTGAAGGGCAAGCACCCTTCACCTTGGAGATGTTGCAGAGCCCCGAGAGCTTC

GGCTTCTCGGGTTTCGGGAATGCCATTGGTTCCTACATGAACCAACAACAAATCACAGACAATGTGTATTCTAGAACCAA

AGAAGAACCAAGGGATGACATGTTTTTCGAATCTTTGCTTGCATAG

>CG14 [organism=Castanea sativa], in silico CDS obtained by short-read mapping

ATGGCTGACGTTCAAATGGGAGACAGAGACACAGAGACCTTCGCGTTTCAGGCAGAGATTAACCAGCTTTTGAGTCTCAT

CATCAACACCTTCTATAGCAACAAGGAGATCTTCCTTCGTGAGCTCATCAGCAATGCTTCTGATGCGTTGGATAAGATCA

GATATGAGAGCTTGAGAGACAAGAGCAAGCTTGAGAGTCAACCCGAGCTCTTCATTAGGATTGTACCAGACAAAGTCAAC

AAGGCCCTCTCAATCATAGACAGCGGTATAGGCATGACTAAAGCAGATTTGGTGAACAACTTGGGAACAATAGCAAGGTC

AGGAACCAAGGAGTTCATGGAGGCGTTGCAGGCTGGGGGTGATGTGAGCATGATTGGGCAGTTTGGTGTTGGGTTCTACT

CGGCTTACCTTGTTGCTGACAAGGTTATTGTGACTAGTAAGCACAATGATGACGAGCAGTACATCTGGGAGTCTCAGGCT

GGTGGCTCTTTCACTATCACCAGGGATGTCAATGGCGAGCCCTTGGGTAGAGGAACAAAGGTCACTCTTTTCCTCAAGGA

AGACCAGTTGGAGTACTTGGAGGAGAGGAAATTAAAGGACCTTGTGAAGAAGCACTCTGAGTTCATCAGCTACCCCATAT

ACCTCTGGACGGAGAAGACCGCTGAGAAAGAGGTCAGTGATGATGAAGATGATGAAACCAAGAAGGAAGAGGAGGGTGAT

GTAGAGGATGTTGATGAGGAAAAGGAAAAGAAGTCCAAGAAGAAAAAGGTTAAGGAGGTGAGCCACGAGTGGCAGCTCGT

CAACAAGCAGAAACCAATTTGGCTGCGAAAGCCAGAGGAGATCACCAAGGAAGAGTATGCCTCCTTCTACAAGAGCCTCA

CCAATGACTGGGAGGATCACCTTGCTGTGAAGCACTTCTCTGTTGAAGGGCAACTTGAGTTCAAAGCTATCCTTTTCGTT

CCTAAGAGAGCCCCATTTGATCTATTTGACACAAGGAAAAAGTTGAATAACATTAAGCTCTATGTTAGGCGGGTGTTTAT

CATGGACAATTGTGAAGAACTTATTCCTGAGTATCTCAGCTTTGTGAAGGGTGTGGTGGATTCTGACGATCTTCCACTCA

ATATTTCCCGTGAAACGCTACAGCAAAACAAGATTCTCAAGGTGATTAGGAAGAACCTGGTGAAGAAGTGCATTGAGATG

TTCAATGAGATTGCAGAGAACAAGGAGGACTATGCCAAATTCTATGAAGCTTTCTCTAAGAATATTAAGTTGGGAATCCA

CGAAGATAGCCAGAACAGGACTAAGCTGGCTGACCTGCTGAGGTACTACTCAACTAAAAGTGGTGATGAAATGACCAGCT

TGAAGGACTATGTAACAAGAATGAAGGAGGGTCAGAAAGATATCTATTACATAACTGGTGAGAGCAAAAAGGCTGTTGAG

AACTCACCTTTCCTTGAGAAGCTGAAGAAGAAGGGCTATGAAGTCCTCTTCATGACTGATGCCATTGATGAGTATGTTGT

TGGGCAGTTGAAGGAATATGATGGGAAGAAGCTTGTTGCTGCCACAAAGGAAGGCCTTAAGCTAGAGGATGACTCTGAGG

AAGAGAAAAGGAAAAGGGAGGAAAAGAAGAAATCATTTGAGAACCTGTGCAAGACAATTAAAGACATTCTTGGGGACACG

GTGGAGAAAGTGGTGGTGTCTGATAGGATTGTAGATTCACCTTGCTGCTTGGTCACTGGGGAATATGGATGGACAGCTAA

CATGGAAAGGATCATGAAAGCACAGGCTCTAAGGGGCAACAGCATGAGTTCTTACATGTCAAGCAAGAAGACAATGGAAA

TTAACCCAGATAATGGCATTATGGAAGAGTTAAGGAAAAGGGCGGAGGTTGACAAGAATGACAAGTCTGTGAAGGACCTA

GTGTTGCTGCTCTATGAGACTGCCCTCTTGACTTCTGGGTTCAGCCTCGACGAACCCAACACATTCGCCTCAAGAATTCA

CAGGATGCTGAAGCTGGGTCTGAGCATTGAGGAGGATGAGAGTACTGGTGAGGATGCTGAAATGCCTCCATTGGAGGAGG

ATGGCAATGAGGAGAGCAAGATGGAGGAAGTCGACTGA

>CG16 [organism=Castanea sativa], in silico CDS obtained by short-read mapping

ATGTTCCACATGAAGAACAATGGCAATGCCTTGTTGAAGGAAAGCGACACAAATTTCAATAATTTTCCCCTTGGTTTGAG

CGTTTTGATTGTGGATCACGATCAGCAAAGTCTGCAAGAAACGGAGGCGAACCTTCGGATATTTGGATATGAAGTTACTG

AATGCAATCGAGTGGAGGATGCTTCAACTCTGCTAAGGATGGAGGGAAGAGTATTTCATATTATTATAATTGAACAATGC

TTGCTTGGTGTGAATGAATTTGAGCTTCTTAGAATTGGTAGAGAGATGGATTTGCCTGTCATTGTGACATCTGAGGATGG

TCAACCTAACAATATTGTTCAATGGAGTTTGGAAAATGGCGCCTGTGATTATCTGCTGAAGCCTATCCCGATGAGTGTTC

TTAAGATGGTGTGGAAATATATGTTTTTCAATAATGTGTCTAGAGTGAAAAATCAGCGGATGTCTTGGACACCAGACTTT

CATGAAAAGTTTGAAGAAAGTGTGCAAAAACTGGGGGGCGCTGCTAGGGNNNNNNNNNNNNNNNNNNNNNNNNNNNNNNN

NNNNNNCTATAAGGGTTTGAAGATGTTGGTAGGGAAAAGGTTTCCAGCCATCTTCAGAAATACCGTGATAGCCTTAAAAA

AAAGTCGCAGCCGCATATGCTGGGGTGCAAAGACAAAGATGGTACCAGTTCAGAGCATATTCAGTCCAGATATGAAATGG

AGGAACATGCACCAATGACTTTGGTTGATCAAGTCTTGAAGACCGCAGTTGCAAATGAGCCAGATATATTATCACAACAG

CAACAAGCTTCAGCAGCAAAGAACACTCAAGGCTTTCAGAGTGTAACATTGCCTTTTGTAACCAACGTACCAACCACTGG

AGGTAGTACTAGTTGTGGAAACCAAAGCAACGCTTTGATGACCCGGATGGATGAAGCATTTTCAAGTGAACAGATGCGGA

ATGAATTCACTGGAGGTCAGCCTTTTGACTACGAAAGCTTTCTCAATGATATNNNNNNNNNNNNNNNNNNNNNNNNNNNN

NNNNNNNNNNNNNNNNNNNNNNNNNNNNNNNNNNNNNNNNNNNNNNNNNNNNNNNNNNNNNNNNNNNNNNNNNNNNNNNN

NNNNNNNNNNNNNNNNNNNNNNNNNNNNNNNNNNNNNNNNNNNNNNNNNNNNNNNNNNNNNNNTTTTAACAACCTCTGA

>CG17 [organism=Castanea sativa], in silico CDS obtained by short-read mapping

ATGGCTATGTTTCAGAAACAAGAGCTTGAAGAAGAATACAGAGGCGCCACGAGAACCGTCGATCTCAGAATCAGCGAAAT

CAGCGGTGCACAAGAAGCGGGGGCCACGTCGCCGCCTAAGCCGAAGGCAGAGCCGGACTCGGAGGAGGAGGACGAGGAGA

ACATGTCACGTGCGATGCAAGTCATGCCAGTAGCTATGCACGTGCCTTCGGGAATTCCCATGACCAAAGCGGTGGCTAAG

AGAGGCTCCACCAAGGACCGCCACACCAAGGTGGAGGGTCGCGGCCGGAGGATCCGAATGCCCGCCACTTGCGCCGCTCG

GATCTTTCAGCTGACCCGAGAACTTGGCCACAAGTCCGACGGAGAAACCATCCGTTGGCTACTCGAGCACGCCGAGCCCG

CCATCATCGCCGCCACCGGAACCGGTACCGTCCCCGCCATCGCCATGTCCGTTAATGGGACTCTCAAAATCCCAACCACG

CCCGCTCCAACGTCCGACCCGAAACCCGGCGACCCACCCGTTAAGAAGAAACGCAAACGACCCGCCAACAGCGAATACAT

AGACATAAACGACGGCGTTTCGGTCTCCTCTGGGCTCGCTCCAATATCAACAGCAACAGCAGCAGCAACAACAACAACAA

CAACCATGCCTCAAATACAAGCAGTGCCTCAAGCTTTGATTCCCATGTGGGCTATACCATCAAACGCCGTCGTTCCGGGC

GCGTTCATTATGGTCCCTCCGATGACGTCAATTCCCGGAGCTCCGAACCCGGCTCATATATTCACATTCCCAGCCACTGC

AACGCCTTTGATTAACATTTCGGCCCGACCCATATCGTCGTTTGTGTCCTCCATGGCTAATATAGCCCCSCCAGTTCAAA

TCCAAGCCAGCTCAGCAGCTTCGTGTTCAAGCTCTGCTACAGCTATTACAACAAGTACAACAACAATTGCTACAACTAGT

GCTCCCACTGCGACTATTTCAACCACTCAGAAGCTTAGANNNNNNNNNNNNNNNNNNNNNNNNNNNNNNNNNATGTCTCG

GTCTTCGAAACACTGA

>CG18 [organism=Castanea sativa], in silico CDS obtained by short-read mapping

ATGGAAGAAGTGAAGCTGCTTGGATTTTGGCCAAGCCCCTTTAGCTATAGAGTGATATGGGCTCTGAAGCTGAAGGGTGT

GAAATATGAGTACATAGAAGAGGACCTAAGCAACAAGAGTGATAGGCTACTGCAGTACAACCCAGTTCACAAGAAGATTC

CGGTTCTTGTTCATGGTGGCAAACCTATAGCTGAGTCTCTAGTTATCCTTGAATACATCGAAGAAACTTGGCCTCAGAAC

CCTTTGCTGCCAAAGGATGCCTATGAAAGGGCATTAGCTCGGTTCTGGATTAAATTTGGAGCAGATAAGGGYCCTATTTT

CTCTGCATTTTTCCGATCTACAAAAGAGGAGCTTGATCAGAATGCAATAAAAGAAATGGTGGAATATCTGAAAAYCTTGG

AAGAGCAAGCTCTAGGGGACAAAAAGTTTTTTGGTGGCGACAATATAGGATTGGTAGACATAGCATATGGATGGCTGTGT

CACTGGTTCATAGGCATGGAAGAAATGGGAGGAGTTAAACTGCTAGGACCAAGTACTGTGCCTCGCTTGCATGCATGGGC

TGAGAATTTCAAGCAACTTCCTATAATCCAAGAGAACCTACCTGACTATACAAAAATGTTGGCACATTTTAAATCATTGA

GGGAGAAAAATACTGCCTCTGATGCCCGTTAG

>CG20 [organism=Castanea sativa], in silico CDS obtained by short-read mapping

ATGGACAAGTACGAGGGAGTGAAGGATTTGGGGGCTGGGAATTTTGGTGTGGCAAGGCTCTTGAGGCACAAGGAGACCAA

GGAGCTTGTTGCCATGAAATACATCGAACGTGGCCTCAAGATTGATGAGAATGTGGCTAGAGAGATTATCAACCACAGAT

CACTTCGGCACCCCAACATAATCCGATTCAAGGAGGTGGTTTTGACCCCCACACATTTGGCTATTGTGATGGAATATGCA

GCCGGCGGAGAGCTTTTTGAACGAATCTGCAATGCCGGTAGATTCAGTGAAGATGAGGCTCGATACTTTTTTCAGCAGCT

GATCTCTGGTGTCAATTATTGTCATTCCATGCAAATATGCCATAGAGATTTGAAGCTGGAAAACACACTTTTGGATGGAA

GCCCTGCACCACGCTTGAAAATTTGTGATTTTGGTTATTCTAAGTCATCTCTGCTGCATTCAAGACCCAAATCTACAGTT

GGAACTCCGGCATATATTGCACCGGAGGTGCTTTCACGGCGAGAATATGACGGCAAGATGGCAGATGTATGGTCATGTGG

AGTGACTCTCTATGTTATGCTGGTGGGAGCATATCCTTTCGAAGACCAAGATGATCCCAGGAATTTTAGGAAAACAATTC

AGAAAATAATGGCTGTTCAGTACAAAATCCCTGACTATGTTCACATATCTCAAGATTGCAAACACCTGCTTTCTCTCATA

TTTGTTGCAACTCCATCCAGGAGAATTACACTTAAAGACATCAAGAACCACCCTTGGTTTTTAAAGAACTTGCCAAGNNN

NNNNNNNNNNNNNNNNNNNNNNNNNNNNNNNNNNNNNNNNNNNNNNNNNNNNNNNNNNNNNNNNNNNNNNNNNNNNNNNN

NNNNNNTGGGGGAGGCAAGAAACCCACCTCCATCATCTAGGCCTGTCAGGGGCTTTGGCTGGGGAGCTGAAGAAAATGAG

GAAGGCAGTGAAGACATAGATGCAGAGGTGGAGGAAGAAGATGATGAAGAAGATGAGTATGATAAGAGGGTCAAAGAGGT

TCATGCAAGTGGAGAATTTCATATCAGTTAA

>CG21 [organism=Castanea sativa], in silico CDS obtained by short-read mapping

ATGGGTATTAGGCTAGTAAATGAAATGGAAGAGGGGAGAGAGAGAGAAGGAGGGAAGGTGTTGAGTCTGATAGAGAAGGC

GACCAACTCCACAGCTGCTGAGGTGGACCCACGTCTCCTTAAGGCCATCAAATCCGTAGTCTGCTATTTGGATACGGAAC

TCCAACTTGCCGCCAATACCCTTTTGGATCTCATGAAGCGCGACCACTCTCAGGTAAGGTACCTGACACTCCTGATAATT

GATGAACTGTTCATGCGYTCGAAGCTTTTCAGAAGCATTCTTGTTGACAACTTGGATCAGGTGCTGAGTTTGAGTGTTGG

ATTCAGAAGAACTCTGCCTCTCCCTGCTCCTCCTGCTGTCGCTTCCATTTTGCGCTCTAAGGCAATTGAATTCTTGGAGA

AGTGGAACTCTTCCTTTGGGATTCATTACAGGCAGCTCAGATTAGGGTTTGATTACCTTAAAAACACCCTCAAGTTGCAG

TTTCCTAATCTACAGGCCAATGCAGCTCGGATTCAGCAGGAGAGAGCAGAACGGGAAAGGCGGTCAAGAGAGATTTTGCT

AAAGAAATTTGAAATGTTCAAGGACAATTTCTCATCTATTAAGGAAGAGATCATGTCTACCATTGAYGAGATTGGGGAAT

GCTTAGACATAGTCCGTACAAATGAGGARTTTATGCCTCTGCCTCCTACAGATGATGAATATTTCGAAGAGTTTCGTTCT

TCTGAACTACTGCAAATCCGTCTCAATACTTTAAAAGAAGGGGAAAAGGTTCACGAGAACACTGACAATAAAGTGGTTTT

TGATGCATTAAGGGAGCTGTACAAGCTTCTAGAGACAAAGCATTTKGTTTCAGTTCAAGAATGGATYTCTTTTCTTCTAA

GGGTTGAAGTRGCAGACAACAGGTTCAGAGATTCCGCTTTAAAGGAGTTAATYGATATMCAAAATCGTCTCAAATCRGTG

AAGAAGAAGTGTGAAGAATCAGGTTGTGCYCTTCCAAACACTGCAGATCGCGATGAAGAAGAAGATGATTTCTGGGAGGA

GGGWAAGATTGGTTCACTTGAGAGCGAGAGATCTACTGTGCCCAATAAGCAAGATGAAGATTTTTCCATGAAAGTAACAT

CTAATAAGTTCAAAAATAGAACTCCTGAAAGCAGTAAAAAAGATTGTAATGACAATGAGATTCTCAGTCCTGAAGGCGGT

GAAACCAATTTGGACCCTTTAAGAAGTAAGCTTCTGGCTGAAGCTCCTGTGATGAAGTGGGGCTCTTTCTTGGATAACTG

GGGTTCAAACAGGAAGGTTTTGGCTAACCAGCGGGGATTGGAGCTTGAAAGTCACTGGGGTAGGGTGGAYTATGACGCGG

TTATTYYAGCTGAGAAAATGCCCGAACTGAATGTACATGCAACTCTTTATGAAGAGCAGCAAACTGACATTCAACCCTGC

AGGGCTCCTTTGAGCAAAGGGGGRCTTTGTCAGAGAAGAGACCTGAGAGTTTGKCCATTTCATGGACCTATYATACCTCG

AGATGATGAAGGAAAGCCACTCAATCARAACTCTTTAAAAGAAGAGATATCTCTTGATTTGGGGATCAATTCCATTGAGC

AGTTAGCAAAACAAGCTGTGAAGAATGTTCGTGAGAGAGATAAAGAAGTAGCAAATAAGAGAGAAATTGATAAAAAGTCA

CTGAAGCGTGCAAAGCTTGCGAAAATTCGGGAGCACAATGAAACAGYTCTAAGGGATGCTGCCTTGACATCAACTTCAAG

ATCTGCATCTRTTGGAGAAGATATGGGGGTGACTGATGGTGAGAAACYGTCAGCYAGAAACAAGAAGGAAACACTCTCAT

CCATGCTGCACAAGAAAGTGACACCAAAAGATAGGATAGCTCAGAGGCTTTTGAATWCACGGGCAAAGGATTCAACAACA

AGACAGCTCACATTGGGTGAAGATGCAAATTACCGAGAAGCCTTCCCAAATCAATGGCAATGA

>CG3 [organism=Castanea sativa], in silico CDS obtained by short-read mapping

ATGGAATCAATCCAATTGGAAAATATCGAAATATCCGCTGATTCATCAAAAGCCAATGATCACGGAGAAGCTCAACATGA

AATCGAGATCGAGAAGAGAGAAATCCCACTACCAGAAGAAGAAGACGAAGACGAAGAATTAGACGACACCGACGACATAA

CCGAAACGTCGTCGTCCGTGACGGACAAGTCATCAATAAACGAGGACGTAGTAAAGATACTCCAACCTCATTCGCAGCTC

CCAAAGCCAGAGGCGCCTCCAGGCCTTTCTAATCCAAACCACAACAACAACAACAACAACGACGACGACNNNATGTCGTT

AATCCCCGGAGCATCCTCAATCGGAAAGTTCATCAGAGAGCGAAGCAACGAGTTCTCTGTAGCAATCGTAAAACGACTGT

CGTCGCTGAAGGAGAACAGCAACGACGACAAAGAGAAAGAGAAAGANNNNNNCAACGAAAACGATGTGACGCAGTTTGAT

CTGTCGGGGCTAAAGGTAATCGTAAAACACAAAAACGACGCGGAACTGAAAGGTCGAATCAGCTTCTTTTCTCGGTCGAA

TTGCAGAGACAGTACAGCGGTTCGCAGGTTCTTTCGGGAGAAGGGATTGAAATACGTAGAAATAAACATCGACGTGTATC

CAGAAAGAGAGAAAGAGCTGGTGGAAAGGACAGGAACCTCGTCCGTACCGCAGATATTCTTCAACGACAAGTTGTTCGGG

GGTTTGGTGGCTTTGAATTCGTTGAGGAACAGTGGGGGTTTCGATCAGAGGGTGAGGGAAATGTTGCGTACGAAATGTCC

ATCGGATGATGCTGCACCTGCAGCGCCTGTGTACGGGTTTGATGACCAGGAGGAAGAATCGACGGACGAGATGGTTTCTA

TCGTTAAAGTGTTGAGGCAGAGATTGCCCATCCAGGACCGTCTGATGAAGATGAGGATCGTTAAAAACTGCTTCTCTGGG

AGAGAGCTGGTGGAGGTCCTCATTCACCACTTTGACTGTGGCCGTAAAAAGGCTGTTTACATTGGATTGCAGCTTAGCAG

GAAGCATTTTATACATCACGTTTTTGGGGAAAATGATTTTGAAGATGGGAACCATTTTTATCGTTTTCTTGAGCACGAAC

CATTTATTCCCAAATGCTTCAATTTCCGAGGATCTATAAATGACAGTGAACCAAAGCCTGCAACTATGATTGGCCAAAGG

CTCACCAAGATAATGACTGCCATACTGGAGTCCTATGCCTCTGATGACAGGCGCCATGTTGATTATACGGGCATCAGTAA

CAGTGAAGAATTTCGGAGGTACATAAATTTGGCTCAAGAACTTCACCGGGTGAACCTACTAGCTCTCTCAGAAAATGAGA

AGTTAGCCTTCTTTTTGAACTTGTACAATGCCATGGTCATCCATGCCGTGATCAGGATTGGTTGTCCAGAGGGAGTAATT

GATAGGAGATACTTCTTTTCTGACTTCCAGTATTTGGTAGGAGGGTATCCCTATTCCCTTAACATAATTAACCACGGTAT

CCTTAGAAACAACCGGAGACCTCCTTACACCTTGGTCAAGCCCTTCGGCACTGGAGACAAGCGTTTGGAGCTTGCTCCTG

CTAAAGTAAATCCATTAGTCCACTTTGGGCTTTGCAATGGGACAAGGTCAAGCCCAACTGTCCGATTTTTCTCACCTGAA

GGAGTTGAATCCGAATTAAGATGTGCTGCTAGAGAGTTCTTTCAGGGTGATGGAATGGAGGTCGACTTGGAGAAGAGGAC

CGTGTATCTCACCCGAATTATTAAGTGGTGCAATGTGGATTTTGGAAATGACAAAGAAATACTAAAGTGGATCCTCAATT

ACTTGGATGCAACTAAATCAGGTCTTTTGACACATCTTTTGGGGGATGCAGGCCCAATAAATATTGTATNNNNNNNNNNN

NNNNNNNNNNNNNNNNNNNNN

>CG5 [organism=Castanea sativa], in silico CDS obtained by short-read mapping

NNNNNNNNNNNNNNNNNNNNNNNNNNNNNNNNNNNNNNNNNNNNNNNNNNNNNNNNNNNNNNNNNNNNNNNNNNNNNNNN

NNNNNNNNNNNNNNNNNNNNNNNNNNNNNNNNNNNNNNNNNNNNNNNNNNNNNNNNNNNNNNNNNNNNNNNNNNNNNNNN

NNNNNNNNNNNNNNNNNNNNNNNNNNNNNNNNNNNNNNNNNNNNNNNNNNNNNNNNNNNNNNNNNNNNNNNNNNNNNNNN

NNNNNNNNNNNNNNNNNNNNNNNNNNNNNNNNNNNNNNNNNNNNNNNNNNNNNNNNNNNNNNNNNNNNNNNNNNNNNNNN

NNNNNNNNNNNNNNNNNNNNNNNNNNNNNNNNNNNNNNNNNNNNNNNNNNNNNNNNNNNNNNNNNNNNNNNNNNNNNNNN

NNNNNNNNNNNNNNNNNNNNNNNNNNNNNNNNNNNNNNNNNNNNNNNNNNNNNNNNNNNNNNNNNNNNNNNNNNNNNNNN

NNNNNNNNNNNNNNNNNNNNNNNNNNNNNNNNNNNNNNNNNNNNNNNNNNNNNNNNNNNNGGTGTTGGATGCTGCCAGAC

ACCAATTCCTAAGCACCTCAAGACATTAAATATTACTCTTCAAAAACTAGGCTCAAACAATATAAGCGATAATTCGAAGA

GTAATATTCCATGCATCTATGCCTTCTTAACAGATCCAACCTTGTTCAATATGTCCACCATAGACCTCCACATTGACCCA

TCAATTGATGATCGGTATCCTACTCCGCCAGTGGTGCTTGATTGGGTGGTGGGAAAGGAAAAGTGCGAAGCAAGTGAAGG

ACCCTCAGGATACGCCTGCGGCGACACTGGAACTTCGTGCATCCCCTCTGACAATGGCCACGGATATCGTTGCTCTTGTC

AAAAAGGATACATGGGAAACCCCTACCTCCGCCAAGGCTGCCAAGTTCTAGGAGCAATCCTTTCATTTGTGATGAGCATT

CTCCTAATCTTCATTCTCTATAAAAGGAGGAGACAAGAAAAAAACTTCACAAAACATGGTGGTTTAGTATTGCAGAACCA

AAGGGTAAGAATATTTAAAGAGGAAGAGCTAATAAAAGCCACCAATAACTATGGCCATCTTCTCGGTCAAGGTGGTTTCG

GATCAGTTTACAGAGGGGTATTAGCGAATGATACCCACATTGCCGTCAAGAAGCCTAGGGGCTTGGACAAGACAGATCTT

AACCTACAATTTCAACATGAAATTTGTATTATTTCCCAGGTCAATCACAAGAATGTCGTCAAGCTCTTAGGTTTATGTCT

ACACACTGACATTCCATTGCTCGTTTATGAATTCATATCAAATGGAACACTCTTTGAACATATCCATAAGACTAAGTCAA

ATTTCTTAAGGTCATGGAAGAATTGTCTAAGAATAGCTGAAGAGACAGCATCCGCCCTAAATTACTTGCATTCTTCAGCA

GACCCACCAATCATTCATAGAGATGTAAAGTCCAGTAACATACTCTTAGATGATCACCATACTGCAAAAGTATCTGATTT

TGGAGCTTCGGTTCTCATTCCAATCGGTCAAAATGGACTAGATATAGCCGTGCAAGGAACTCTCGGTTACCTGGACCCTG

AATATCTTACAACAGGTAATTTAACCGCAAAAAGTGATGTATATAGTTTTGGAGTTCTCCTCGTAGAGCTCTTAACAGGA

CAGAACCCGTGCACTTTTACTAGGTTTGGAGAAAGGAGTAACCTCATTCAATATTTCATCTCTTCGTTACTAAACAAAAA

TCTCTTTCAAATTTTGAATGTTGAGGCCATTGATAGAACTGAAAGGGAGCAAATACTGGAAGTTGCTAAACTGGTAATGA

ATTGTCTTAATGGTTGTGGGGAAAAAAGGCCAACAATGATGGAAGTGGCAGAGGTTCTTGCTAAGCTAATGAGAGTGTCC

CGTGAAAACTTGTTAGTTCAGCATAACAGTGAAGATACAGAGAGCTTAACTAGTGACTTACCATTCCATGTTTTTGAGTT

TGAGGATGCAACTTCTCAGATGGCCTGCCAATTATCTGAATTCCATCTCAGTGAAACCCCATTGATATTGAACCAAGTTG

ACTCGTCGATATATTTATGA

>CG7 [organism=Castanea sativa], in silico CDS obtained by short-read mapping

ATGGCCACTTTGTTCGTAATTTCTAAGCACTTTTATGCCCATGTTGTACCCAGTGTCTGTCAATTGTTTCGTGCTCATGT

TCGTCCTCATCTCCGTGCTTTTTCCATTATATATTTATCCCTAGTCCTTGGGCTTGCGTACTTGGCTATAATCNNNNNNN

GTTACAGACAGTTTCAAAAATTGAAGGACGAGTTCAAGTGGCTTAGATTTGAAGAGAAGTTTATGAATGCTCTCATAGAA

GATGTAAAATACGTTTCAGATTTGGGGAAAGAAATAGATAAGAAAGCCCATCACCTTTGGGATTCACAAATGAGACCAGG

AGCAACTGTACAACTAAGCGATGTTGAGAAAGAATGGGTAAAAGCAATCGATGCAGTGGTTCATAAGGCAAAGTCTTGCC

GCCAAACCTACCAGAGACTGAGTCGAAGAAGACAATTTCTGAGGTGGGTTTTCTCCGTTGTAAYGGATTTCMGAAAAATC

AGTGSCCTCCGCACTGAAATAGATGCTGTGATCGATGAGATGTATGATCATCTCTGCAGGAAATGTAGAAAGATCTATGG

ATCACTAGAGAGGTCAAGGTCTATTGTTAGAAGCCTGCAGGACAGACCACTGGTGGAACAAAACTCTTATACTTATAATC

GAGCTGTGCGACTGCCACCAACTCATTCTATTGAAGAGAAATTGAAGGACCTAATCCATAGAAAGCCGGATTTAGTACGA

GACAAGAAAGACGACATACAGTACAAAATTGAGTTTCGGGTGAAGCTACTGCTTGCTTTCCTGGAAGATTTAGATGGGCT

TAGAATGGACACTAAAACGGAGAAGGCCTGGGTGAAGCTTGTGGAGGAGGCCATACTCGAACTACAGCAAGACATGGAGA

GCATACTGAAAATAGCAAATCGAATGAGTTGGATTTTCTATATCGGTAATTGGATAGCAAAGCGTAAGCTCAAGAAGAGC

CATGAGCACTAYGAAAGAAAGTTTCGGAACCTCATTGCACATAAGGACATCTGTGATTTCAAATTTATTAGAAGAGATTC

ATTAAAATCTGTCCATCGGTCGCAGAAAAAGCAAATGTTTTCATCTGAGGCCACAGCAGCCAGTGACGATGGTATTTCTT

CTCATCTGAACAACTTTCACAATCAGCTAAGTCAGGGGCAAGCCATGGAAGATTTCACACAACTACACAGTCGTTTAAAG

AATGTACACAAACTTCTCATGGATGCAAAAGCAATTGAAGGTATAAAGCATTCAAGAATGGCTTGGACGGATCAACTGAA

GRAAATTATTGAAGATGCAGGAAAATCATTGAAATCCCATGGAGAAAGTTCMCGMTCGGAGTGGAGTGAAAATCAAAACA

AGACAGAGTCTCCGTCAAAGTTCAAAGAAATTAGGCGATTTRATATAGCACTCAGAATTCTCAGCAGAAGCATAACAATA

TTTCGGATCGAACAGAGGAGAGAGGAGAACTTGGTAGTCGGCATGGATGAAGATGTACATAAARTAGTCTCACAACTAAT

CACCAACAGTGAGAATTGCTCCACGCATTTTATTGTGGGGATGAAGGGCATCGGTAAGACAACACTGGCGAAGATGGTTT

TTTACCATAGTGCTATTCAGAACCATTTTGARTCCAGATACTGGGTGCCTCTAACTGATAGAGTGGATGAGGAAAAGAAT

GTAATCCTGAAAAGATTAGGACAGACGTTAATGCCTGCACCGGCAACCAATAAGGAAGGGAAGGAAAAAGAGGAANGNNC

NNNNNNNNNNNNNNNNNAAAAANATTACTCRATCAAGGAGCTGAATGGGTTCTTGAAGGGTAAGAAGTACCTTATAGTTC

TGGACAGCATCTTGTCAGTGGAAGCCTGGGAGAGTTTAAAAGCCGCATTCCGGGATAGCACAAATGGGAGCAGAATTTTG

ATCACCACACGGCACRAGAGTGTAGCTTCAAATGCTGAGCACTACTACMAACTTCGACTACGAACCAAAGATGAGAGCTT

GAGTTTGTTTCAACAGATGGTCGACCTTCCCTCTGAAACTTCTGACCAGCCTGAMCTCTCTCCAGAAGCAGGAGAAKCWC

AAAAASTGAATTCRCTCGCACRYAAAGKAGTAGGAAGGTGTGGGGGCTTGCCACTTTCCATCTTAAGTCTTGGGTATCTA

TTTTCAGGGACGGAGAATGTGACTTATGAGAAGTTGTTGAAGGTGCTTGACCATATTGATCATAACCAAACACCATGGAC

GGAAATTATAGATTCCGAAGTAAAAGACTTGCAACCACCTCTAGGGCAATGTCTTTCTTATTTTGGACTCTTCCCCAAGG

ACACCGAAACTTCAGCAAGGAGATTAGTTGCTTTGTGGGTTGCAGAAGGGTTGGTAAARCCAAGTGAGCAAGAGCCTCCT

GAATTAGTTGCAARGAAKATTYTGARAGAGTTGATAARCCKCAACTTGGTACAAGTGGTGGAAAGAARGCCTAACGGGAA

GGAAAAGACATGTAGTTTCCCTAGTGCTCTGCGAGATTTATGGTTGCGATCAAATGCAAGCTTTGATCAGCGGNNNNNNN

NNNNNNNNNNACTCTTAGTCATGRWMRYGRTAAAARTTTACAAAATTTATTGCGAAGCTGCAGAAATCCYCGCTCAATTC

TCTTTTTTGATACTCGAGAAGGWAATAAACCTGGAGAAGAAATAGGTAACTTCCTTTCTCTGGGTATTGCAAGYGGTCAT

CTMTTACAGTTACAAGTCCTTGATCTTGAACAYGTATTTCGACCCCAATTGCCAGACACTATAGGAAAATTAATTCATTT

AACATATTTGGGGTTAAGGTGGACTTACCTGGAGAAGATCCCAYCGTCAATTGGCAACTTAACMAACCTCCAAACTCTGG

ATGTGATGCATACTTATATCCGTGTTCTCCCTAGTTCAATATGGAAATTGAAGAAACTTCAGCACCTATGCATGAACCAA

ATTTATCGAAGCCAAATCAAGCATCAATCAAGTGGGAGTTCTTTGCAAAATCTCCRAACAYTGAGGGGTGCATTTGTAGA

TAAGGACAGTCCTCTAAAGGATAGCCTCCGTAGGTTGACCAACCTTAGAAAATTGGCACTAGCTTTCCAACTGAACCTGT

CACAGCAAATAGCACTAGCTGAGAGTCTTTTGAAMCTGAAGMAGCTTAAAGCTTTGAAGTTGAAATCAATTGATGAAATG

GGTCAGCCTCAAGATCTAAAGGCGACTTTCTTGTTAGGCCTTGAAAATCTTTCCAACCTATATCTGTTTGGAAAACTAGA

GAATGCATCCATTAATAGACTCCCACAAAGCCTAACTGACCTTACCTTATCTGCCTCTGGACTTTTAGATGATCCAATGC

CAGAGTTAGAGAAACTTAAAAAGCTTGAATGTCTCAGTTTGTACTCTGGTTCTTATACTGGGAAAAGCATGTCATGCTCC

AAGGGAGGCTTTCCCCAGCTTCATGTTCTGAACTTCTGGATGCTTAAGGAATTGGAAGAATGGAATGTGGTGGAGGAAGC

AATGCCAAAGCTCAAGAAGTTRGAGATTCGTTCCTGCAACAGTTTGAAGGTCCCAACTGGGTTGGGGMMTCTRAAGACTC

TTAGTGAATTGAAGTTAAAGGATATGCCRGTGAAATTCATAGCAGAAATTGAGAAAACCAAGGAGATAATTTGGGRCGAC

ATTGCTCTATCTCCRGTCATAAATTATTGA

>CG9 [organism=Castanea sativa], in silico CDS obtained by short-read mapping

TCKCTCCTCAAAACACTTTTCGATCAATTCATCCACAAATATTTCCAYGAAGCCGTTGCTAAGRTGACTCTCAWAGACGC

TTTCCTCTTCCTTATTGTTCACTCCATTGATAAGTTGGGGATATGGCCTCGAWTACCAGTGATCTYAGGYCTCTTCTATC

TGGGAATTCGCYGGCACCTTCATCAAGAGTACAACTTGTTCAACGTCGGTAGATCTGCGGTCRGGGTTCGGTTTAACCCC

ATGGANNNTTATCCATATAGGACAGCTGATGGAAAATACAATGATCCCTTTAATGGAGATGCCGGCAGTGAAGGAACTTT

CTTTGGTAGAAATGTTCTCCCTGTTGATCAAAAGAATAAGTTAATGAAACCAGATCCAATGGTGGTGGCCACAAARCTTC

TTGCTCGGAGAAAATTCAMAGACACAGGRAAGCAATTCAACATGATAGCAGCTTCTTGGATTCAGTTTATGATACATGAT

TGGATCGATCACTTGGAGGACWCCAAGCAGATTGAGCTGACTGCACCTAGAGAAGTTGCRAACCARTGCCCTCTCAWGTC

ATTCAAGTTTTACAAGACAAAGGAGGTTCCAACTGGCTCTTACGATATCAAATCTGGTGCACTAAACATTCGTWCACCAT

GGTGGGATGGAAGTGCTGTTTATGGGAGCAATGCAGAAAAGTTGCAMAAAGTAAGAACTTTCAAAGATGGGAAGCTTAAA

ATATCATCAGATGGGCTTCTCCTCCATGACAAAGATGGTGTYGCTGTGTCAGGAGATGTTCGTAATAGTTGGATTGGTRT

CTCAACATTGCAGGCCCTTTTCATTAAAGAACACAATGCAATCTGTGACGCCCTCAAGAGAGAATATCATCACTTGGATG

ATGAAGAGTTGTATCGTCATGCAAGGCTAGTGACATCTGCAGTTATTGCTAAGGTCCATACCATAGATTGGACCGTGGAG

CTCCTCAAAACTGATACGTTGCTTGCAGGGATGCGAGCCAATTGGTATGGTTTGTTGGGAAAGAAATTCAAGGACACATT

TGGACATGTTGGAGGAGCCATCTTAGGAGGTTTCMTAGGTCTAAAGAAACCGAATAATCATGGTGTTCCTTATTCATTGA

CTGAGGAGTTTGTTAGTGTTTATYGGATGCACTYGCTTTTACCCGATTATCTTCATTTAAGAGACATCTCAGCTGCACCY

GGGCCAAACAAATCTCCACCAYTGTYMGRAAAGGTTCCTCTACCAAATTTGATTGGTCTTARAGGAGAAACGGCCTTGGT

AGAAATTGGGTTTGAAAAGCAAATGGTTTCAATGGGCCACCAAGCTTCTGGGGCYCTGGAGCTTTGGAACTATCCTACAT

GGCTTAGAGACCTTATACCACAAGAYGTGGATGGCCGAGATAGGCTTGATCATGTGGACTTACCAGCCCTTGAAGTGTAT

AGGGATAGGGAGAGGAATGTTGCAAGGTATAATCAGTTCCGTAGGGCTTTACTATTGATACCAATTTCAAAATGGGAAGA

TCTAACGGATGATAAAGAAGCAATTCAAACACTTGAGGAAGTGTACGGTGATGATGTTGAACAACTYGATTTGCTTGTGG

GTCTCATGGCARAGAAGAAGATCACAGGGTTCGCAATTAGTGAAACGGCTTTTATAATATTCTTACTAATGGCAAGCAGG

AGGCTGGAAGCAGATAGGTTCTTTACAAGCAATTTCARTGAGGAGACATACACGAAAAAAGGACTTGAATGGGTTAATAA

TACAGAGAGTTTGAAGGATGTGCTAAAACGTCATTATCCAGAAATGGTAGAGAAATGGATGAACTCTACAAGTGCTTTCT

CGGTGTGGGATTCACCTCCAAATGCTGACAACCCAATCCCACTCTATCTTCGTGTTGCTCAGTGA

>CG10 [organism=Castanea sativa], in silico CDS obtained by short-read mapping

ATGCAAAATATGGAACTGGAAGGAGGAGAAAGAAGAKCATCATCAGGAAAAGATGATGGGTTTAGAGCAAAATATGTTGA

AGAAGCAAAAAAGCAAGCAGGGCTAGCAGGGCCTCTYATAGCCGTTAGTATATTGCAGTACAGCTTACAAGTTATATCAG

TGATGTTCAATGGTCATTTAGGAGAGCTGCCTCTTTCTGGTGCTTCCATGGGATCTTCCTTTGCTTCAGTCACTGGTTAC

ACTGTCCNNCTAGGAATGAGAAGTGCATTAGAGACAATGTGGGCAAGCCTATGGAGCAAAACAGTATCACATGCTTGGTG

TTCACACACAAAGAGCTATGCTAACCCTCCTGAGCTTAAGCATCCCTCTAGCAATAATTTGGTATTGCACAAGCAATATT

CTCATAGCTCTACACCAAGACMATGAAATATCTAATGAAGCTGGAGTTTTCAATCGTTGGATGATCCCAAGCCTTTTTGC

CTANGGTMTYCTTCAATGYCTAAACAGATTTTTACAAACACAAAACAAKGTTATTCCTATGTTGATAAGCTCTGGATTCA

CGGCTTTGCTACACGTTCTTGTTTGTTGGGTTCTTGTGTTTAAAATCAAGCTTGGGATTAAAGGAGCTGCCTTGGCAATT

TCCATTTCCAATTGGATTAATGTGCTTTTGTTGGCAATTTATGTAAGATTTTCCCAAGCTTKCRWGAAAACTTGGACWGG

GTTTTCCAAAGAAKCCTTKCATGATATTCTYAGCTTYMTAAACATTTAACTCTATGAAAATTTTGACTTACAGCTTCGAA

TATTGGTCCTTTGAAATGATTTTTTTCTTATCTGGACTTCTACCAAATCCAAAACTAGAGACATCAGTGTTATCAATAAG

CCTTAACACGTGTTGGATGGTTTATATGATTTCGAGAATTTTCTTGGTATTGTATAGCACAAGAGTGTCAAATGAAATGG

GTGCTGGATGCCCACATGGTGCACGTTTGGCTCTTCGTGTCTTGATCACATTTGCTCTCTCTGAGGGTGCAACAATAGCA

GTTACTACACTTTTGGTGCGCAATGTTTGGGGAAAGCTTTATAGCAGAGAAGAAGAAGTTATTAGATATGTTGCTAGAAT

GATGCCACTGCTTGCATTATCAGACTTCTTGGATGGATTCCAATGTGTACTTTCAGGTCTTAAAATCTTGTCACAAGTAC

TACTCAAAGGCTGCTAGAGGATGTGGGTGGCAAAATCTATGTTCATTTATAAATCTTGGCGCTTACTATTTTGTGGGAAT

TCCTTCTGCTGTGCTCTTTGCTTTCATCTTTCATTTTGGAGGCATGGTGAGGGCTTTGGATGGGTATCATATGTGGACTT

TCTGTTCAAGTTGTGGCACTTGTAGCAGTAAACTTATTCGCTGATTGGGATAAAGAGGCAAAGAAAGCTGTGCATGTTGC

TCAGGGAGGTGACCTTACTCTAGGTATGGGAAGCGCATTAGAGACTCTATGTGGACAAGCCTATGGAGCAAAACAGTACC

ACATGCTTGGTGTTCACACACAGCGAGCTATACTAACCCTCCTAGGCTTAAGCATTCCTCTAGCAATAATTTGGTATAAT

ACAAGCACCATTCTCATACTTCTACACCAAAACCATGAAATAKCTACTGAAGCTGGAGTTTTCAATCGTTGGATGATYCC

AAGCCTTTTTGCCTATGGTATCCTTCAATGYCTAAACAGATTTTTACAAACACAAAACAAYRTTATTCCTATGTTGATAT

GCTCTGGATTCACARCTTTGCTACATGTTCTTGTTTGTTGGGTTCTTGTGTTTAAAATCAAACTTGGGATTAAAGGAGCT

GCCTTGGCAATTACCATTTCCAATTGGGTTAATGTGCTTTTGTTTGCGATTTATGTAAGATTGTCTCCAGCTTGCAAGAA

AACTTGGACTGGGTTTTCAAAAGAAGCCTTGCATGATATTCTCAGCTTCCTAAAGCTAGCGGTTCCTTCAGCAATTATGA

TATGCCTAGAATATTGGTCATTTGAAATGGTTGTTCTCTTAGCAGGACTTCTCCCAAATCCAAAACTAGAGACATCGGTG

TTATCAATAAGCCTTAACACATGTTGGTTGGTTTATATGATTTCTGTTGGTCTTGGTGGTACTATAAGTACAAGAGTGTC

AAATGAATTGGGTGCTGGGAGCCCATATGGTGCACGATTGGCTCTTCGTGTGATGATCATAGTTGCTCTCTCAGAGGGTA

CAATAATAGCAATTACTACAATTTTGGTGCGCAATGTTTGGGGAAAGCTTTATAGCAAAGAGGAAGAAGTTATCARATAC

GTTGCTAAAATGATGCYACTGCTTGCATTATCAGACTTCTTGGATGGATTCCAATGTGTWCTTTCAGGGGCTGCTAGAGG

ATGTGGGTGGCAAAATCTGTGTTCATTTATAAATCTTGGTGCTTACTATCTTGTGGGAATTCCTTCTGCTGTGCTCTTTG

CTTTTGTCTTCCATTTTGGAGGCAAANNNNNNNNNNNNNNNNNNNNNNNNNNNNNNNNNNNNNNNNNNNNNNNNNNNNNN

NNNNNNNNNNNNNNNNNNNNNNNNNNNNNNNNNGCAAATAAAGCTGTGCGTGGTGCTCAAAGAGGTGGCAGCGTAATTGT

TGAGGAGGAAGAGGAACTATAG

>CG12 [organism=Castanea sativa], in silico CDS obtained by short-read mapping

ATGGAATTGGACCCAGATGATGTGTTCAGAGACGACGAAGACGACGCCGATAATGAATTCTATCGGGAAAAGGAGTCGAC

CAAAGAGCTTCTGGTTTACCTTGTCGATGCTTCCCCCAAAATGTTCTCCGCTTCTTGCCCTTCCGGAGATCAAAACGATG

AAACCCATTTTCAGATTGCTGTTAGTTGTATCTCGCAGTCGCTGAAGACCCAAATCATTAATAGATCGTATGATGAAGTT

GCTATTTGCTTCTTTAACACCAGGGAAAAGAAAAATTTGCAGGAGTTAAATGGTGTTTTTATATTTAATGTTGCTGAAAG

GGANNNGTACCTAGACAGGCCAACAGCAAGGCTTATAAAAGAATTTGATTGCATAGAGGAAAGGTTTATGAAAGAAATTG

GGAGTCAGAATGGTATTATCTCTGGGACTCGAGAGAATTCTCTCTACAATGCTCTTTGGGTAGCACAAGCACTTCTGCGT

AAAGGATCTGCAAAGACAGCTTATAAACGGATGCTTCTTTTTACAAACGAAGATGATCCTTTTGGGAATATCAAGGGAGC

GACGAAAATAGATATGACAAGAACCACATTGCAGAGAGCTAAAGATGCCCAAGACCTAGGCATCTCAATTGAACTTCTTC

CCTTGAGTCAGCCTGATGAGGAGTTCAATGTTTCACTTTTCTTTGCTGATTTGATTGGATTGGAAGGGGATGACCTTGCT

CAGTTTTTACCCTCAGTGGGAGAGAAATTGGAAGATATGAAGGATCAGCTGAGAAAGCGAATGTTCACGAAGCGCATAGT

TAAAAAAATCGCATTTTCAATTGCTAATGGATTATCAATTGAACTGAATACATATGCTTTGATACGTCCCACTATACCAG

GAGCAATTACCTGGCTTGATTCTGTTAGCAATCATCCCTTGAAGACTGAAAGATCTTTCATCTGTTCGGATACTGGTGCA

TTGTTGCAAGAACCTCTTAAGCGCTACCAACCTTACAAAAATGAGAATATTAAAATTTCAGTGGAGGAGCTATCGGAGAT

AAAAAGAATTTCTACTGGACAGCTTCGTCTTTTAGGATTCAAGCCATTAAGTTGCTTGAAAGATTATCATAACTTGAGGC

CATCGACATTTATTTTTCCCAGTGATGAGGAAGTGTTTGGTAGTACTTGCATTTTCATTGCCCTGCACAGATCCATGTTA

CGGCTTGAGCGATTTGCAGTTGCATTTTATGGTAGTTCTTCTCATCCACAGCTGGTTGCCCTTATTGCTCAAATGTGTAA

GAGKTGTGGGGAGTCGGTGGATCATCTTCTACTTCATTGCTCCATAGCTTGGTAGCTTGGGAGTTGTGGTCTTTGGTTTT

CTGCTTGTTTGGTATTCAATGGGTTATGCCTCATAGAAAGTTTGCGCGACATYGTCACATTGATGTTTGGAAATTAGTGC

CTCACTGCTTGATTTGGTGCATTTGGCGAGAAAGGAATGCTAGAAGCTTTGAGGGTTGCGAACGCTCTTTGCTGGAGATT

TAAGTCTTTTTTCTTACACACTCTCTTTGAATGGAGTGTGGTTTTTTCTCATTTTTCTTGTTCTTCCTTTTCTATTTTTC

TTGACCGTTGTTCTTTTGTTTCTTGAATGGTTAGCCTTACCAATCTCCTGAAAAATTCTCAGAAAGAGATTATCAGCCAT

GGTGGTCAGGTTGAGCCGCCAGGAATGCACATGATATATCTTCCATACTCTGAAGACATCAGACATGTTGAAGAGCTTTA

TTCAGATTCAAATGGTGCTGCACCAAAAGCAATTGATGATCAAATTGAGAAGGCTACTGCTTTAATGGAACGTATCGATT

TGAAAGATTTTTCAGTATGCCAATTTTCYAACCCTGCCTTGCAGAGGCACTATGCAGTATTGCAGGCCTTGGCTTTGGAG

GAGCCCGACATCCCAGAAACGATAGATGAAACCCTCCCTGATGAGGAAGGCATGGCTAGACCGGGGGTAGTTAGTGCAGT

AGAAGAATTCAAGCTTTCTGTCTATGGTGACAACTATGACGAGGAAAAGGACCTTGTGGGAAATGGAAAAGCAAGTGAGA

TCTCAAAAAAACGAAAAGCTATTGCTGATAATGCGGTTAAGGAGTCTGCAAACTATGATTGGGATGACCTTGCAGACAAT

GGGAAGTTGAAGGATTTGACGGTGGTGGAGTTGAAATACTACTTGACAGCACACAACCTTCCTGTTGCTGGAAAGAAAGA

GGCTTTGATCAGCAGGATATTAACTCACATGGGAAAGTAA

>CG15 [organism=Castanea sativa], in silico CDS obtained by short-read mapping

ATGGCAACTTTAGTCACTACCATTCTCTATAAAATCTTGTATATCCTGACTTACGCCCTTTTCTATTTCGTTCTCTTTGT

TATCTTCGTCATCATCCTAAGCATTTACTGCTGTTGCTGTCGGAGAAAAAAAAAAAGTCAGACATCGACCATGAGTCGAA

TAAAAAAGATTGGGAGTTGCTGCGTAGTTTTCAAAAAGACGTGGAAAACGTTTTGAACTCTGGCAAACCGATGAAGACCA

TCAGGGAATGACTGCACAACTACAAGAATCTGTGACAAAGTGGCATGAAAAAAGTATGAAGGATCTCCCTAATAGTGCTG

AATTCAATGATAAAAGTTATAAAAGCAAGGATGAAGACGAAWAACTCAAGAACATGGTACTTGAAGCCAAAAATATCCAC

CGAAATTTGGACAAGTTAAAGGACAAGATCAGATACTTGCAGGTCAGACCTACTGATGATGAACGGCAGGCTCTTGTGCA

AGAATTCAAAGAATTCGTTAAGGGTCTCATCGCTGGTGTGCAGGAAGTAGACAATCTCATCCCTGAAGTGAAAAAGGAAG

ACAAATTCATTGCTGATATCATTGCTGATGTGCAAGAAAAAGACGAACCCATTGCTGGCACTTCAGATTTACCTGGTGAT

ATTAAAGCGGAAATACAGTCGTTCACATTGTATKGGGCACCATTCCGTGCTTTCCTGATCGATTTACAAAGTTCTGGTCT

TGAAACTGAAACGGAAAAGKCCTGGTTRAAGGAAGCGAAAGAAATCACTGATAAAGCGCAGCATGCTATCGATAAGTTTC

TAAAGTATACCAGAAAATGGAAGTGGATACTCTCATATTTTAGTAATAAGAAAGMAAGGAAAGAGCTTAAARCGGAGATG

AAGCGCATTAACACAGGGTTCGAGGAATTCCTCGAGAGAAAGGAGAAGTTCGGTATTCAATTTAAAAGATACTCCACCAG

AAGAGAAACAGACCCATTACAATCCATATCTGGAACCTCACAAGAGCAGGCATCCAATAATGTGCAGATCTCAGAAMATG

GCAGCGGTAATTTTAATAAGGAATTAGAGAATCTCAAGAAGTATGAGAAATCGCTCCAAGAGAAGTCTCTTAAAGGGCTC

CAAGAATTATGCACGACGTTTACCAACGTCCACGGTGAAGCTGCAGAAAATGGAGAAGGCATAAAAAGTTCAACAGNNNN

NNNNNNNNNNNNNNNNNNNNNNNNNNNNNNNNNNNNNNNNNNNNNNNNNNNNNNNNNNNNNNNNNNNNNNNNNNNNNNNN

NNNNNNNNNNNNNNNNNNNGAGAAGTCCCGCGAAGAAACTAGAAGAAGTTTGACCAAATTGAGGGCGCTTTGAAAATTAT

TGAAAAAACAACAAAAGCATACAAGTTTGAGATTAGAAAAGAGATGGGAGTAGTCGGTTTGGATGAAGACATACAGGCAG

TGGCCTCACAACTGATAACTGAAAATAAACATGTTGTTTGCGTTGTGGGGATGAAGGGTATCGGTAAGACCACCCTGGCA

AAGAATACATATAGCCACCGTGATATTTTGGATCATTTTCYGGCACGTGCTTGGGTGACTCTAACTGATCTAGAGACCAA

CGATTACGATGCCATTTTTAAAGATGTGGCAAAGCAGGTCTCGGAACCTGATATTAATGGATCACAGGAGGAAGAGAACA

GAAGGGGAGAGTGGAATAATAAGGTATGTGCTATCTTGAAGAAGAAGAAGAAGTATTTCGTGGTTCTAGATGATTTCACA

GCGGAAAATGACTGGGAAACTCTAAAAGCAGCATTTCCAGAGACAGGAGGGAGTAGAATTTTGCTCACCACACGTAACCA

GTGTGTTGTTAGGACAGYGTGTGGCAAACTCCATTGTCTTAGATTACGAACCAAAGAAGAGARCTGGAGATTGTTTATCC

AGATGATGGGAGATGGCTTTGATTCTGAGGACGTAAAAAAATTAGCTGAAGAAACTGTTGGACAATGTGGGGGCTGGCCA

CATCAAATTTTACATTTAGGGTATCTCATGTCAGCGAAAGATGATAAGTTGCAAGAATGGCCCAAGWGCATCACTCCRGC

TCAGGAACCATGGCTCAAATACCTGAAAATAGAAGGTCGTATTAAACTCGAAAATTGGCTTGAGAGTCATAAAATAGAAA

TTAATGATTGGTCTGACCATGAAGGTAAAAATGATTTGCTTGACCATCTGAGCAAATTTTTTGCTTTCTTCAAACTCTTT

CCTAGAGACTATGAAATTCCTGCAAGGAGATTGGTCGCTTTGTCAGTTGCAGAAGGGTTGGAGTTGGGGGAAGAAGGTCG

TGACAAGAATAAAATCACGAAACTAGTTGCAATGGATTATTTGTCAGTGTTGATTGATCTCAACTTAATTCAAGYAGTTG

AAATTATTAAAGGGAAAGTAAAGACATGTCGCCTGCCCAGTGCTCTCGAAGAACAGTTCTTGCTTGAAAGTTGAAAGTAA

GATTTCACAGTATCATCTTGTTGATCTCCTTGACAAAAACTCTGAAATTTCTAATGATATTCATGGTAAGGGTACTAATT

CACCAAAATTTCAGCAAAGTTACAGAGATCTCATCTCCTATCTGTCATTTGACACTAGAGAAGGAAATAAACCTGGAGAA

GATGTAGGGAAGTTTCTTCGTAAGGGCATTGCAAGTGGGTGTTTCCACGGTTTAAAGGTTCTTGATCTTGAACATGTATT

CAGACCTCAATTGCCCAACACCATCGAACAATTAGTTCAGCTGATGTATTTAGGGTTAAGGTGGACTTACCTGGAGGAAA

TTCCATCATCCATAGGCAACTTACTGAACCTCCAAACCCTTGATGTGAAGTATACTTRTTTGCAGAAACTTCAACACCTA

TACTTGAACGAGAGTCATCGAAGTAAATTTGTGTCTCAACCAAGGAGCAGTTCCCTAAAGAATCTGAAAACATTGTGGGG

TTTATTCTTGGNNNNNNNNNNNNNNATAAAGAATGGGCTGAACAAGCTAAACAACCTTACAAAGCTAGGACTAGCGTTCC

AGCTGGAGTTGCCTCAGCAAGAAGAATTGGCCCGTTGGGTGAAAGGTCTGAAATACCTCAAATCTCTGAGATTGAGATCG

GTTGACGAGAACGAGATTCAATCCATCAAAAACCTATTTCCAGACACCTCTGACTTTCATCTGTCTCACCTTACGTTGTC

TGCCTCGGGGGGTCTCTCCAGTCTTAAATCACTCTGTTTCTACTCTGACTCTTACAAAGGAAAACATATGCTTTTCATTA

AGAAGAGCTTTTCCCAACTTCAGGTTTTGAGACTTTGGAATCTAGAGAAGCTAGAAATGCTGACGGTGGAGGAAGAAGCA

ATGCNNNNNNNNNNNNNNNNNNNNNNNNNNNNNNNNNNNNNNNNNNNNNNNNNNNNNNNNNNNNNNNNNNNNNNNNNNNN

NNNNNNNNNNNNNNNNNNNNNNNNNNNNNNNNNNNNNNNNNNNNNNNNNNNNNNNNNNNNNNNNNNNNNNNNNNNNNNNN

NNNNN

>CG19 [organism=Castanea sativa], in silico CDS obtained by short-read mapping

ATGGCTGACAGTGTTGTTTCTTTGCTCCTGGAGACTCTGAACCAGCTGCTAGACCAAGATGCTGATCTGCTTCCTGAAGT

GGAGGATGAGCTAAGATCATTCCACGCGGAGCTTGGATTGGCAAGTGGCTTTATTAGAAACTCTGTGGGTCAAGGGAATG

CCGGAGAGGTGGAAGTTATGGCGGAGGAAATCAACAAGGTTGCTTATCTGGTTGAGGATGACATTGACNNNNNNNNNNNN

NNNNNNNNNNNGCAAAGGATGAGAGGTGAGTTCAGGAAGTTCATTTATGGCTTTGACCACAGGAACAAGCTTCGAGATGT

CTCAGGAACTATCCACAGCAGCATGAAGGAGGTCAAACAAATTTACGGTGAAAGAGGAAGCAGTGTCCAAGCTAGCAGCC

ATTCATTTTTAGCAATTGACAGGAAGATCGAGGAAGTTGACCTGCTATGCTTTCCCGACAAAATGGAGAGCTTATTGAAG

CAGCTATCTAACTTGGACCAGCGTCGTGCGGTTATTTCAATTGTTGGAGAGGYTGGAATCGGCAAGACTGCTCTTGCCAA

GAAAATCTATAATAAAAGTTCTGTTCAGGGTCAATTCAAATACCGTGCATGGGTCCATGTATCTCGGTATTGCAGAGCTG

GAGAGTTGTTGTTTGGAATTTTAGAGTCTCTTCAGCTGATCACTACTGGAGAGAGGCAAGACATGACAGAGAAAGAGCTG

CAAGAGAAGCTATCGAAGTACTTAGCAAATAGGCGGTATCTGATAGTCCTGGATGATCTGCGGAAATTAGAAGCAGGATG

GGAGGAGATCATAGAACAGGCCTTTCCTGATGATATGGATSGAAGTAAAATCTTGTTCACTTGTCGGCCTGAGCATGAAC

CTTCGCTTAATCGTACAACCATCCACCAACGCTATTTACTTGAGCGGCTTGATAAGGCAGAGAGGTGGCAAGTCCTACGC

GAGAGAGTGTTCAGTGCAGTAACTAATAAAGCAAATTTGGAATCACTGGGGGAACAGATGGCAGAAAAATGCRAGGGGCT

ACCACTCTCACTTGCAATTTTAGGCCGATTGTTGGTGATGCAGGGGGACAGTGTTGAAGCCTGGTCCAGATTAGCTAGTA

CTTTAGATCAAAGCATAGATCTGCATTTGCTTCTAAGCTTTATTCACGAACATTTACCCGACCACTTGAGCCTCTGCCTT

CTCTATTTTGGTATGTTCCCAGAAGGGGCTGAAATACCCACAAAGCAACTGATCCAATTGTGGATGGCCGAAGGATTTAT

TCGGCAAAAGGGAAGTCGAAGAATGGAGGACTTTGGACAACAGTACTTGGAGGAGCTTATTGGTCTCCACTTGATCCAAG

CGACTGCAAGGAGAAACGATGGCGGAGTGAAGACGTGCCAAATCCATGATCTTATCTGGAAGTTTTGCATTTCCAAGGGT

AGAGAAATGAAGTTTCTTGAAGTCCCAGATAAATCAAGTGATGCTCCTGAAAAGACTGGGGAGAAGACTCGAAGAGTAGC

CATTCATGGTACTTTTTCTTCGCTGAAGGATGTTTTCTCAGAACTCCACAACTGTAAAGGTGTCCGTTCTTTCCATTTTT

CTGTCCCAGGTGATAATGTTAGGCTTCCCACATTTAAGTGGAATTCACTTTATGAAGGCTTCAAGTTGCTCAGGGTGTTG

AACCTTGGGATGGTAGAAGTCTTGGAAATTCCCAAGGAGGTTGGAAGGTTAATTCATTTGAGGTACTTGAGGATAAGAGC

TCCAGGCATCAGGCATGTGCCTTCCTCTATATGCAATCTCCTAAATCTTCAGACACTGGACATGAGAGAGTCTAGTTTGA

GCAACTTGCCAGATGAAATTTGGAAGCTGCAGCAGCTACGACATCTCAAATTGTTTCATCTCCGTAGCCTGCCTGAGCAT

TGGGGAGCTGATGAGAAATCTCTTGAGAACCTCCAAACCTTGTCTTGCATGCGTCCCCAGAAAGGAATGAAGCGCCTCAT

GGTCAGGGCCAGGTTCCCTAATATTAACAAATTAAAACTAGCTAGCCAAAACCCTGATGAAACAGCTGATTTTCTTGAAA

GCCTTGACCATCTATACCACCTTCAATCCCTCAAAATTGAGTCACCTTCTAAACTTCCTGATCCAAGTGCATTTCCATTG

ACACTCACCAAAATAACTTTACAGGATACAACTCTTGGGGACAGTTGCATGAAAAGGCTAGAGAAGCTGCCTAATCTTCG

CATACTCAAACTACGAAAAAATTCTGTTTCTATAGAGGAGATTACTTTCAATGCAGGTGGGTTTCCTCAACTCCAAGTCC

TCCAAATGGTGGAACTGGAAATAACTAAATGGACACAAGGAGGAGAAGCAATGGTGAATCTTCGGCATCTGGTGATACAA

AAATGTGTTCAATTAACAGATTTTCCAAAGAGCTTGTCTAGTCTGCGAAAACTAGAGGTGATTTTGCCCACTGCAAATCT

AAGAAAGGCCCTTCAAGATTATGGGTGTAAGGGTGGTCTTGAGATGGTGATAAATCCTCCCCTGGACATGTATGATGAAT

CAACAATGATCAATCCTCCCCAGGATGAGTCTAATGCATCAACAAAGATCAATCCGCCCCAGGACGTGTCTGATGCATCA

ACAATGATCAATCCTCCCCAAAACGTGTCTGATGCATCAACAACAATCAATCCTCCCCAGGATGTGTCCGATGCATCAAC

AACAATTAATCCTCCCCAGGACGTGTCTAATGCATCAACTTAA

>CG22 [organism=Castanea sativa], in silico CDS obtained by short-read mapping

ATGGAAGAGGGGAGAGAGAGAGACGGAGGGAAGGTGGTGAGTCTGATAGAGAAGGCGACCAACTCCACAGCTGCTGAGGT

GGACCCACGTCTCCTTAAGGCTATCAAATCCATTGTCCGCTATTCGGATATAGAGCTCCGACTCGCCGCCAATACCCTTT

TGGATCTCATGAAGCGCAACCACTCTCAGGTAAGGTACCTGACACTCCTGATAATTGACCAATTGTTCATGCGYTCGAAG

CTTTTCAGAAGCATYCTTGTTGACAACTTGGATCAGKTGCTGAGTTTGAGTGTTGGRTTCAGAAGAACTCTGCCTCTCCC

KGCTCCTCCTGCTGTYGCTTCCATTTTGCGCTCTAAGGCAATTGAATTCTTGGAGAAGTGGAACTCTTCCTTTGGGATTC

ATTACAGGCAGCTCAGATTAGGGTTTGATTACCTTAAAAACACCCTCAAGTTGCAGTTTCCTAATCTACAGGCCAATGCA

GCTCGGATTCAGCAGGAGAGAGCAGAACGGGAAAGGCGGTCAAGAGAGATTTTGCTAAAGAAATTTGAAATGTTCAAGGA

CAATTTCTCATCTATTAAGGAAGAGATCATGTCTACCATTGAYGAGATTGGGGAATGCTTAGACATAGTCCGTACAAATG

AGGAATTTATGCCTCTGCCTCCTACAGATGATGAATATTTCGAAGAGTTTCGTTCTTCTGAACTACTGCAAATCCGTCTC

AATACTTTAAAAGAAGGGGAAAAGGTTCACGAGAACACTGACAATAAAGTGGTTTTTGATGCATTAAGGGAGCTGTACAA

GCTTCTAGAGACAAAGCATTTKGTTTCAGTTCAAGAATGGATYTCTTTTCTTCTAAGGGTTGAAGTRGCAGACAACAGGT

TCAGAGATTCCGCTTTAAAGGAGTTAATYGATATMCAAAATCGTCTCAAATCRGTGAAGAAGAAGTGTGAAGAATCAGGT

TGTGCYCTTCCAAACACTGCAGATCGCGATGAAGAAGAAGATGATTTCTGGGAGGAGGGAAAGATTGGACCACTTGAGAA

TGAGAGATCTACTGTTCCCAATAATCACGATGAATATTTTTCCATGAAAATAACTTCTAATAAGTCAAAAAATAAAACTM

CTGAAAGCAGTAAAARAGATTGTAATGACAATGAGATTCTCAGTCCTGAAGGCGGTGAAACCAATTTGGACCCTTTAAGA

AGTAAGCTTCTGGCTGAAGCTCCTGTGATGAAGTGGGGCTCTTTCTTGGATAACTGGGGTTCAAACAGGAAGGTTTTGGC

TAACCAGCGGGGATTGGAGCTTGAAAGTCACTGGGGTAGGGTGGAYTATGACGCGGTTATTYYAGCTGAGAAAATGCCCG

AACTGAATGTACATGCAACTCTTTATGAAGAGCAGCAAACTGACATTCAACCCTGCAGGGCTCCTTTGAGCAAAGGGGGR

CTTTGTCAGAGAAGAGACCTGAGAGTTTGKCCATTTCATGGACCTATYATACCTCGAGATGATGAAGGAAAGCCACTCAA

TCARAACTCTTTAAAAGAAGAGATATCTCTTGATTTGGGGATCAATTCCATTGAGCAGTTAGCAAAACAAGCTGTGAAGA

ATGTTCGTGAGAGAGATAAAGAAGTAGCAAATAAGAGAGAAATTGATAAAAAGTCACTGAAGCGTGCAAAGCTTGCGAAA

ATTCGGGAGCACAATGAAACAGYTCTAAGGGATGCTGCCTTGACATCAACTTCAAGATCTGCATCTRTTGGAGAAGATAT

GGGGGTGACTGATGGTGAGAAACYGTCAGCYAGAAACAAGAAGGAAACACTCTCATCCATGCTGCACAAGAAAGTGACAC

CAAAAGATAGGATAGCTCAGAGGCTTTTGAATWCACGGGCAAAGGATTCAACAACAAGACAACTCACATTGGGTGAAGAT

GCAAATTACCGAGAAGCCTTCCCAAATCAATGGCAA

>CG23 [organism=Castanea sativa], in silico CDS obtained by short-read mapping

ATGGTAGGAGCAGAGGGAGGACAATCCTTGGAGCAAACGCCAACATGGGCAGTAGCAGTAGTGTGTTTTGTGCTGGTTGT

AGTTTCTATCATCCTTGAACATGTCATTCACTTAATAGGAAAGTGGTTAACAAAGAGACATAAAAGTGCTCTTTATGAAG

CTCTTGAAAAGATAAAATCAGAGCTTATGCTATTGGGGTTCATATCCTTCCTCCTAGCAGTAGGACAAGGACCCATTTCG

AGTATATGCGTAACAAAGGCTGTAGGGGCAACATGGCATCCCTGTAATAAAAAGCAAGAGAAAAAACTAAACAAGGACGA

AGAGAATGGCCACCGGAGGCTTCTAACAATTTCAGACTTTGGCGGCGGTGGTGATACTCGACGTGTTTTAGCCGCTGCAG

GATATGACAAATGCGCAGACAAGGGTAAAGTTTCTTTTGTCTCAGCGGATGGCATCCATCAACTCCATATCTTCATCTTT

GTGCTTGCAGTTTTTCACGTACTTTACTGTATAATCACTATGGCTTTGGGCAGAGCAAAGATGAGAAAGTGGAAGGCATG

GGAAATGGAAACAAGGACAGCTGACTACTATTTCTCCAATGACCCAGAAAGATTTAGGTTTGCCAGGGACACATCGTTTG

GGCGGCGGCACTTGAGCTTTTGGAGCCGATCATCAGTCCTTCTATGGACAGTTTGTTTCTTCCGACAGTTTTTTAGATCA

GTCCCCAAAGTTGATNNNNNNACCCTGCGCCATGGTTTTATTGTTGCACATCTAGCACCTGAGAGTCAAACAAAGTTTAA

TTTCCAGAGATACATCAAGAGGTCACTTCAAGAGGATTTCAAAGTCGTTGTTGGTATAAGCCCAACAATTTGGTTTTTTG

CCGTATTGTTTCTACTATTTAATACCCATGGTTGGTTTTCTTATCTGTGGCTGCCCTTCCTTCCACTGATTATARTTTTA

ATGGTTGGGACAAAGCTACAAGTCATTATAACCAAGATGGGTTTGAGAATTCAAGAGAGAGGAGGGGTGGTTAAAGGAAC

TCCACTAGTTCAGCCTGGTGATGACCTTTTCTGGTTCAATCGCCCTCGCCTCATTATTTATCTTATCCACTTTGTTCTCT

TTCAGAATGCCTTTCAACTGGCCTTCTTTGTGTGGACTTGGCATCAATTTGGSTTAAAATCTTGCTTYCATGAGAAGTTG

GAAGATGTGATCATCAGGATATCTATGGGGGTCCTCATACAGATACTATGCAGCTATGTGACTCTTCCTCTATATGCTTT

GGTGACACAGATAGGTTCGACCATGAAGCCAACAATCTTCGACGAAAGAGTGGCAAATGCACTACGAAAGTGGCACCACA

AAGCAAAGAAAAACATAAAACAGAATCAACATTCAACCAGTACGCCAGAAACCCCAATGCATTCCATGTCTCCTGTCCAT

CTTCTTCATCACTAYCAAGGTGAACTAGACAGCATTCATAAAACGCAAAGGATGTCCAATTTCAAYAATGAAGGTWGGGA

TATGAAKGGCCCCCATTCATCCTCTCACCACCATGATCARRYATCATTKCCTMAGAACCAGAGAAACCATGGACAAGAAG

AGAGCRGTGCTCATGWGCCGAGCTCCACACATTCKCCTTCATCTCGTCATCCATTTCGTGTTCAGCATGAAATTGACGTT

CACTCAATRGATTTCTCATTTGACAACAGCTAG

>CG24 [organism=Castanea sativa], in silico CDS obtained by short-read mapping

ATGTCACGACGTGGCGGCGGTAGGCAACCGGACTATCGTCGTGATCAACCGTCACCGGCGTCTCAACAAGGCAGTGGAAG

AGGCAGAGGCGCTGGTGGCGGACGAGGCGACGGCGGTGGACGTGGCGGACGAGGCGACGCTGGTGGACGTGGCGGACGTG

GTGACGCTGGTGGACGAGGCGCCGGAGGTAGAGGCGCCGGTGGAGGAGGAGGAGGAGTAGGCCGCGGTGCTGTTCCTTAT

AGTTTCTCTCCGGTGCCTGCTCCGGTTGTTTCACAACCATCATCGTACGCTCCACCTTCAACTTCGGCTCCTCCTCCGCC

TCAGCCTCGGCCTCAGCCTCAGCCTCAGCCTCAGCCTCAGCCATCGTCGTCCACGGTGGCTGAGCTTGCCAGAGGTGTCG

AAAGGACAGTCACATTGCAGGAGCGAGCTCAGCCTTCGTCCTCGAAAGCCTTGGTCGTTCCGAGAAGGCCTGGTTACGGA

ACCGTCGGAAAGAAAATCCAAGTCCGAGCAAATCATTTTCTCGTCGAAGTCGCCGATAGAGATCTCCACCACTACGATGT

GACTATAACGCCTGAGATAACATCAAAGAAAGTGAATCGGAGTGTAATGACTCAGCTAGACACAATGTACCGAGAGTCAC

ATTTTGGTAACAGAAGGGCTGCTTATGATGGCAGGAAGAGCCTTTATACTGCAGGAGCATTGCCATTTACATCCAAAGAG

TTCATTGTCAAGTTACAAAATGAGGACCATCCTACATCTTCATCTTCTGGTTCTGCGAGAAAAGAACGCCAATTCAAAGT

GGCAATTAAGTTCGTTGCTAAGGTTGACTTGCAGCACCTGAAAGAGTTTTTAATCAGTAGGCAGTCAGATGTTCCGCAAG

AAACAATTCAAGCTCTTGATGTGGTATTCAGAGCAGAACCATCATTGAAATATACTGTTGTGGGGAGGTCATTTTTTCAT

CCTAGCTTGGGTACTCAAGGTGAATTGGGTGATGGTGTTGAGTTTTGGAGAGGCTACTACCAATCTCTCAGGCCAACACA

GATGGGGCTTTCTCTAAATATAGATGTGTCAGCCAGAGCGTTCTACGAACCACTTCGTGTGACTGACTTTCTTGTAAAAC

ATTTCCGTTTTAATCTGTCAAAGCCTCTGTCTGATCAGGACCGTCTTAAGATTAAAAAGGCTTTGAGAGGAATAAAGGTA

GAGCTTACTCATACGGAGTATGCAAAAAGTTACAAAGTCAATGGTGTTTCGCCACAACCGGTTAGCCAGACAATGTTTAC

CCTTGATGACAAGAAAACAAAGACATCAGTTCTTCAATATTTTCATGACAAATATAACATTGTTCTCCAGTATGCGTTTT

TGCCTGCCCTTCAAGCTGGTAGTGATTCAAAACCAGTTTACTTACCGATGGAGGTCTGTGTGATTGCTGCTGGACAGAGA

TACACTAAGAGATTAAATGAAAAACAAGTAACTAACCTATTAAGAGCAACGTGTCAACGGCCAATTGACAGGGAACGGAG

TATCACAGGGATGGTAAAGCATAATGATTTTAGTAGAGTTAAGCTTGTACGTGAGGAATTTGGAATTGAAGTACGAGAAG

GACTTACAACAGTTGATGCTCGAGTGTTGCCGTCTCCAATGCTGAATTATCATGAAACTGGGAGAGAATCAAGGGAACAT

CCTAGGATGGGGCAATGGAATATGATAAACAAGAAAATGGTCAACGGTGGTAGAGTGTCATATTGGACGTGTGTGAATTT

CTCTGCTCGGGTGAACAGAGATTTTCCGTTCCAATTCTGTGAGAAATTGGTCAACATGTGCAACAGCAAAGGAATTGATT

TTTACCGAGACCCTTTGATACCAATACATTCAGCTCATGCTGGTCAAATTGATAGGGCTCTCATGGATATTCACAAGCAA

TGTGCAGCCAAACTTAAAGAAATTGAGCCAGGAAAACAGCTTCAGCTGTTGATAATTATTTTGCCTGATGCTAYTGGGTC

ATATGGGAAGATTAAACGTATATGTGAAACGGAGCTAGGAATTGTTTCTCAGTGCTGTCAGCCCAAGCAAGCATCAAAAC

TTAGTATGCAGTACCTGGAAAATTTGGCTCTCAAGATCAATGTGAAGGCTGGGGGACGTAACAATGTGTTAACTGATGCT

ATTCAAAAAAGAATTCCTCTTGTATCTGATCGCCCTACAATTATATTTGGTGCGGATGTGACACATCCACAACCGGGAGA

GGACTCTAGCCCTTCAATAGCAGCGGTAGTGGCTTCTATGGATTGGCCGGAGGTAACCAAATACAGAGGACTTGTCTCTG

CCCAGGATCACCGTGAAGAAATTATCCARGATCTCTATAAATCAGTGCAGGATCCTCAAAGGGGTTTGGTTCATGGAGGC

ATGATCAGAGAACAACTTATTGCTTTCAGAAGAGCAACTGGGCATAGACCCCACAGGATTATTTTCTACAGAGATGGTGT

TAGTGAAGGACAATTTAGTCAAGTTCTGCTTTATGAGATGGATGCAATAAGAAAGGCCTGTCTCTCACTAGAGGAAAATT

ACCTGCCGCCAGTTACTTTTGTTGTTGTTCAAAAAAGACATCATACACGCCTCTTTCCTACAGATAATCARACRGACAGG

AGTGGCAATATTCAACCAGGTACTGTTGTCGATACKARGATTTGCCATCCAACTGARTTTGACTTCTACCTTAACAGCCA

TGCTGGTATACAGGGGACTAGCYGGCCTWYRCASTATCATGTGTNNNACGATGAGAACAATTTTTCTGCAGATCTCTTAC

AAGTGCTAACCAATAATTTGTGTTACACGTATGCAAGGTGCACTCGATCAGTTTCCATAGTTCCTCCTGCATATTATGCC

CATTTAGCAGCTTTTCGAGCACGCTATTACATTGAGGGTGAAACATCAGATGGTGGTTCTACTGGGGGTGAGAACAGAGC

AGAGTTNNNNNNNNNNNNNNNGATCAAGGATAACGTGAAAGATGTCATGTTTTATTGCTGA

>CG25 [organism=Castanea sativa], in silico CDS obtained by short-read mapping

ATGGCAGCTGATGCACTACTTACTGATCTCTYTAAGCAGTTGGCTTCAATCKCTKTTCAGCTGGCTAAACAAGAGATCAA

GTTGCTTGTTGGCGTTGATGAWGAAGTCCAAAAGCTTCAAGACAAGCTYGGAWTCATCAAGGCAATGCTGGAYGATGCCG

AKAAAAGACATGCAGTGAAGCKGGATACTGAGAAGCTTTGGTTAGAGCAGCTCCAAGACAAATACTAYGAGATGGATGAC

GTTTTGGACACCTGGAGCACTGCAAGGATCAAAGCAGAGATAGAGAAAGAAGAAGGAAAACCAGCTGATATTAATGCTCC

WGCTGTTGTGAAGAAGAAGGTATGCTCCTTYTTCCCATCTCCATCATGTTGTTTTAACCTTCCACTGCGTCATGATCTTG

GTCACAAGATTAAARRACTGAATGAAAAATTAGATRAGATTTTCAATGACAGAGAGAAGTATGGGATTGACTTTAACAGG

CAACCTGAAGTAGTTGTGCGACCAATAACTACGTCCTTTGTGGATGAGTCCGATATAATTGGTCGTGATACATATAGGGA

TGACCTACTGAGYGTCCTCTTAGGCAAGGGTAGTCCAAAGGAAAGAAAACCCCATGTCATATCTTTGGTGGGCATGGGAG

GTCTKGGAAAAACYRCTCTWGCCCAAATAGCCTAYAATCATAGTGAGGTGMAGGCCCATTTTGATAAAAGAATGTGGGTT

TGTGTTTCTGATCCTTTCGATCAGTGCAAGRTTGCCAAAGCAATCATTGAATCTYTTGAGGSTCNNNCTCCCARCRCTRC

TGAATTACAAAGTTTGTTAAATAAACTTTATAGTTTGATTGAGGGAAAGAGGTTCTTTCTTGTCCTAGATGATGTGTGGA

CTGAAGACTCCACAAAATGGGAGCCCTTCARAAATGCACTCAAATRTRGTGCCGAAGGTAGTAGAATTCTAGTGACCACA

CGTAAAACTAGGGTTGCAAACATGATGGAGAGTTCTCYCATAATCAATTTGGGGGTATTGTCCTCTGATGTCTGTTGGTT

GATTATCAAGAAAATAGCATTTTCTGATGATTATGGAGAGCAATATAGGGATTTAGAAGACWTGGGCAAACAATTAGCAA

ATAAGTGTCAAGGTTTGCCACTTGCTGCAAAGATTCTAGGGAGTTACCTACGTGGCAAGATGAGTAAAAAAGAGTGGGAG

AAGGTTTTGCACAATGATTTATGGAAATTAGAAGATATTGAAAATGATTATCAATTCAATAAATTTGAGTTAATTATCAA

TTGGATGGCACAAGGRTATATCAACTCAAAAGAAAATATGGAGATGGAAGWSARAGSAGAATCAATGGCAAAAGATGTGT

GCTTCAAAATCAATTATAGTGGNNNNNNNNNNNNNNNNNNNNNNNNNNNNNNNNNNNNNNNNNNNNNNNNNNNNNNNNNN

NNNNNNNNNNNATGGCAGCTGATGCACTACTTACTGATCTCTYTAAGCAGTTGGCTTCAATCKCTKTTCAGCTGGCTAAA

CAAGAGATCAAGTTGCTTGTTGGCGTTGATGAWGAAGTCCAAAAGCTTCAAGACAAGCTYGGAWTCATCAAGGCAATGCT

GGAYGATGCCGAKAAAAGACATGCAGTGAAGCKGGATACTGAGAAGCTTTGGTTAGAGCAGCTCCAAGACAAATACTAYG

AGATGGATGACGTTTTGGACACCTGGAGCACTGCAAGGATCAAAGCAGAGATAGAGAAAGAAGAAGGAAAACCAGCTGAT

ATTAATGCTCCWGCTGTTGTGAAGAAGAAGGTATGCTCCTTYTTCCCATCTCCATCATGTTGTTTTAACCTTCCACTGCG

TCATGATCTTGGTCACAAGATTAAARRACTGAATGAAAAATTAGATRAGATTTTCAATGACAGAGAGAAGTATGGGATTG

ACTTTAACAGGCAACCTGAAGTAGTTGTGCGACCAATAACTACGTCCTTTGTGGATGAGTCCGATATAATTGGTCGTGAT

ACATATAGGGATGACCTACTGAGYGTCCTCTTAGGCAAGGGTAGTCCAAAGGAAAGAAAACCCCATGTCATATCTTTGGT

GGGCATGGGAGGTCTKGGAAAAACYRCTCTWGCCCAAATAGCCTAYAATCATAGTGAGGTGMAGGCCCATTTTGATAAAA

GAATGTGGGTTTGTGTTTCTGATCCTTTCGATCAGTGCAAGRTTGCCAAAGCAATCATTGAATCTYTTGAGGSTCNNNCT

CCCARCRCTRCTGAATTACAAAGTTTGTTAAATAAACTTTATAGTTTGATTGAGGGAAAGAGGTTCTTTCTTGTCCTAGA

TGATGTGTGGACTGAAGACTCCACAAAATGGGAGCCCTTCARAAATGCACTCAAATRTRGTGCCGAAGGTAGTAGAATTC

TAGTGACCACACGTAAAACTAGGGTTGCAAACATGATGGAGAGTTCTCYCATAATCAATTTGGGGGTATTGTCCTCTGAT

GTCTGTTGGTTGATTATCAAGAAAATAGCATTTTCTGATGATTATGGAGAGCAATATAGGGATTTAGAAGACWTGGGCAA

ACAATTAGCAAATAAGTGTCAAGGTTTGCCACTTGCTGCAAAGATTCTAGGGAGTTACCTACGTGGCAAGATGAGTAAAA

AAGAGTGGGAGAAGGTTTTGCACAATGATTTATGGAAATTAGAAGATATTGAAAATGGTCTTTTGGGGCCATTTTTTTTG

AGTTATTATGAATTGTCTCCATCAGAGAAACAATGTTTCTTATTTTGTGCTGTCTTTCCAAAAGATTATCAATTCAATAA

ATTTGAGTTAATTATCAATTGGATGGCACAAGGRTATATCAACTCAAAAGAAAATATGGAGATGGAAGWSARAGSAGAGC

ACTACTTTGAAAAKTTAGCCATGCACTCTTTYTTTCAAGACTTTRARAAAGATRAKAATGATGGTAAAATTGTAAGTTGC

AAAATGCACGATATTGTGCATGACTTTGCAGAATCAATGGCAAAAGATGTGTGCTTCAAAATCAATTATAGTGGNNNNNN

NNNNNNNNNNNNNNNNNNNNNNNNNNNNNNNNNNNNNNNNNNNNNNNNNNNNNNNNNNNNNNNNNNNNNNNNNNNNNNNN

NNNNNNNNNNNNNNNNNNNNNNNNNNNNNNNNNNNNNNNNNNNNNNNNNACCCAAGGAACTTGAGAAATTACCTCAAGGG

ATAGGTWGGTCCATTTTGGAACTTCCAAATGAGGTGGAAAAATTGATACATTTAAGATTACTCAAGTTATCTTGTAAKGA

GATAAAAGAATTGCCTGAAAGTATATGTAATTTATGTAATTTACAAAGTTTGGATGTTAGTGAGTGTTGGGAACTTGAGA

AATTACCTCAAGGGATAGGTAAACTAATTAATTTAAGACACCTCCNNNTGCTTGATGAAGGTTTTGAACAAGAAGGAATA

AAATCGTTTCCAAAGGGATTTGGGAGATTAACTAGTCTTAAAACATTAGGATACTTTCCTGTAGGTAAGGGCGAAGAAAT

ATGTAAATTGGGAGAATTAGAATATTTGAACCACATTCAAGGGAAACTTKGGATAYTTGGGTTGAAAAATGTGGTAGATT

TTGGTGCGATTGAGAATACATTGAAGAAGAAGAACCACCTCCGTGATTTGYRKCTAGTATTTGACACATTAGWRGWNNNT

GACACATTAGAGGAGGAAGAGGAAGAGGAAGAGGAAATTAAGACGGAGGAAGAAAGAAGAAGAAAAATGGAGAAAGATGT

AGCCATTCTAAATGCCTTAGAGCCACCTCCCTGCTTGGAATCTTTAMTAATTGGTTWTTTCTATAAGGGCACCACAATGT

ATCCCAATTGGATGATGTCCAAATTGACCTATTTRAAAARRCTTARWATCAGARACTGCCCAAACCTGAAGCTTTTGCCT

CCTTTGGGGAAGCTGCCGTTGCTCGAAGAATTASAAATAGMTWKSACTCCAATGATTCGAAAGGTGGGAGATGAATTTTT

GGGAATAGACATAGAAGAAGAAGAATTATCAGAATCATCCAAAAACAATAACAGCAAAGACATCATCATATTCCCAAACC

TTARATCTCTCRMATTYYKWYRTTTGKRGAAGTGGGAAGAATGGACTGGGATGGGAGGAACAATAGAAGAAGAAGAAGAA

GAAGAAAAAGATAATAGTGCTAATGCTTTTGTTACTAATAATACTCCAAATATTAAAATAATGCCACTTCTTCATTCCTT

GCAWATTWRGGAKTGCGRCAGTCTAAAGTCTCTGCCAGACTACCTGCGTAATACACCATTACAGAAATTGGTAATCKATG

AMTGTCCAATTCTCGAGCAGCGTTGCGAAAGAGGGATAGGAGAKWACTGGCCCAACATTTCTCACATTCCAAACATCCRA

ATTGATTRKAAGTACGTGCAAAGAGACGGTCAACTTGGAGACAGTGCAGATTCAGAGGTAAGGGCGAAGAAATATGTAAA

TTGGGAGAATTAGAATATTTGAACCACATTCAAGGGAAACTTKGGATAYTTGGGTTGAAAAATGTGGTAGATTTTGGTGC

GATTGAGAATACATTGAAGAAGAAGAACCACCTCCGTGATTTGYRKCTAGTATTTGACACATTAGWRGWNNNTGACACAT

TAGAGGAGGAAGAGGAAGAGGAAGAGGAAATTAAGACGGAGGAAGAAAGAAGAAGAAAAATGGAGAAAGATGTAGCCATT

CTAAATGCCTTAGAGCCACCTCCCTGCTTGGAATCTTTAMTAATTGGTTWTTTCTATAAGGGCACCACAATGTATCCCAA

TTGGATGATGTCCAAATTGACCTATTTRAAAARRCTTARWATCAGARACTGCCCAAACCTGAAGCTTTTGCCTCCTTTGG

GGAAGCTGCCGTTGCTCGAAGAATTASAAATAGMTWKSACTCCAATGATTCGAAAGGTGGGAGATGAATTTTTGGGAATA

GACATAGAAGAAGAAGAATTATCAGAATCATCCAAAAACAATAACAGCAAAGACATCATCATATTCCCAAACCTTARATC

TCTCRMATTYYKWYRTTTGKRGAAGTGGGAAGAATGGACTGGGATGGGAGGAACAATAGAAGAAGAAGAAGAAGAAGAAA

AAGATAATAGTGCTAATGCTTTTGTTACTAATAATACTCCAAATATTAAAATAATGCCACTTCTTCATTCCTTGCAWATT

WRGGAKTGCGRCAGTCTAAAGTCTCTGCCAGACTACCTGCGTAATACACCATTACAGAAATTGGTAATCKATGAMTGTCC

AATTCTCGAGCAGCGTTGCGAAAGAGGGATAGGAGAKWACTGGCCCAACATTTCTCACATTCCAAACATCCRAATTGATT

RKAAGTACGTGCAAAGAGACGGTCAACTTGGAGACAGTGCAGATTCAGAGNNNNNNNNNNNNNNNNNNNNNNNNNNNNNN

NNNNNNNNNNNNNNNNNNNNNNNNNNNNNNNNNNNNNNNNNNNNNNNNNNNNNNNNNNNNGCCGCCAATGCAACMCGAAG

GTCCAGACGGAGAATCTGAGCTCCTACGCCATGGTTAAGATCGAGTATTCGGATGGCACGAAGGACGGTGGGGCCCAGAG

GACTAGTCCGGCGGGTCCCACATCTCCGGCCGGGAGGTCGGTGCCTGGAATCGTTGTTAAAGAAGACGCCANNNNNNNNN

NNNNNNNNNNNNNNNNNNNNNNNNNNNNNNNNNNNNNNNNNNNNNNNNNNNNNNNNNNNNNNNNNNNNNNNNNNNNNNNN

NNNNNNNNNNNNNNNNNNNNNNNNNNNNNNNNNNNNNNNNNNNNNNNNNNNNNNNNNNNNNNNNNNNNNNNNNNNNNNNN

NNNNNNNNNNNNNNNNNNN

>CG26 [organism=Castanea sativa], in silico CDS obtained by short-read mapping

ATGGCAACTTCATTTACTACCATTCTCTATAATTTCTCTCATTCCCTGTCTGATGCCATTTTTCGTTTCATTATCGACTT

TGTTATCATCATCGTAATCGGCCTAACCATTTACTACTGTTTCTGTGGGACAGAAAAAAATAATGACAACGACTACAAGT

GGATTAAAAATGAATGGAAGTTGCTGAGGCTTCTTCAAAAAGACGTGGAAAGCGTTTCGAAGTCCGATTCTGAAATGGAT

AAGGAAGTCTATAATTTCTGKCAAACCGAAGAACCCAACCAGGGAATGACAGCACGACTAAAACCAACTGAGGAAGAATG

GTATAAGAAAAGCACAAAGTTAGAGCGTCTCTCTTTAAATCTTGAAGACACTGATAAAACTTCTAAAAGCATGTCTGGAA

CCGCTATTCGTGGCTTCAAGAAAGAAATAGATCAACTCCTTGGTAAAAAGAAGACGGAGGCTAGAGACATCTACAAAACA

CTGGAGAATTTAAAGTCTAAGATCAKAAAGTTGCAGATCAGACCAATTGATGATGAACACAGCGCTGATGATGCGCACAA

AGAAGGCAAAGATCCCATTGCTCATCTCGTTGATGATGTGAAAGAAGTAGACGAACTCATTCCTAATGTGAAAAGAGAAG

ACAGAAGCTTTTCTGCTCCCCCTGCTGATGTTAGAGCTGAAATACAGAAATTCAAAATGCATCTGGCACTAATCCGTGCT

TTCCTGACTGATTTACAAAGGTTTGGTCTCAAAACTCAAACGGAAAAGGCTTGGCTGGAGGAAGCAAATGAGATAGTTTC

TGATAAAGAAACTATCAAAACTTTGATAAAGAAAACCAGAAATCCACAAGGGATGAGTTTATATTTACTCCTAACTCGGA

GAGCAATGAATGAGCTTAGGGACGAGATGAAGCGTATCAACACAGGGTTCGATGAATTCCTCRAAAGAAAGGTGAGGTTC

GGTTTTCAATCTATCAGACCATTCATCAGAAGAGAAACAGAGCCATCAAAATCCGACTCTGGATGGCTTCYATGTTGAAT

TTATGCAACAAGTTTAAGGAGGTCCATGGAAAATTTGAAGAAGCAAAATCTATAGAAGGCATAACCAGTTCAACAGAGGA

AAGGTYGAAGCAAATGAAGAAATTGACTGAAGATACAGAGTATTATTTAAAAAAATATAMAAAGAATTCAACTGAGCTGA

GTTACTTTTTTAAACATTGGAGAAACCCTAAGAAGACACTTCGCGAGAACTTGCAACAAATCGACGGCGCTTTGGATATT

CTTGCAAAAACTACGGAAGCATATAGCTTTGAAATTGGGGAAGAGATGAGAGCAGTCGGATTGGATGATGACATACAGGA

AGTGGTCTCAAAACTGAAGAAATCTGGAGACGATGCTAAACATTTCGTTTGCATTGTGGGGATGAAGGGTATCGGTAAGA

CCACCCTGGCGAAGAATATATATGAKYACAGTGATATTGTGGATCATTTTCCGGCTCGTGCTTGGGTGACTCTAACTGAT

CAAAAGGCCAACGACTACGATGACGTTTTTAAAAATCTAGCAAAGAAGGTATCAGACTCAGAACCTAAGAATAAGAGGTA

CCTTGTGGTTCTCGACAACTTCACAGCAGAAAATGAAAATGTCTKGAAARCTCTAAKAGAAGCATTCCCAGCAGGGACWG

ATGGGGGTAGAATTTTGCTCACCACACGTGACAGGTGTGTAGCTGAGGAAGTTGATCAGAGTATCGAACTTCATCATCTT

AGGTTACGAACCAAAGAAGAGAGCTGGAGATTGTTTATCCAGATGGTGGATGATTTTGGGTCTGCTACTAATGATGTAAA

AAGACTAGCTGAAGAAATTGTTGGAAGATGTGTGGGCTTGCCACTTCAAATTTTGCAATTTGGGTATCTTATGTCCGGGA

AAGATGTGAAGGATACAAAGTTGTCAGAATGGCTGAAGCGCATCAATCCGCCTCAGAAACCATGGCTTGATCATATCCAG

GGAGTAGGAATTCCTGATAGGCCTGATTATTGGAAAAAAGTTTTTCCTTTCTTCAATCTCTTTCCTGGAGACTGTGAAAT

TCCTGCAAGGAGATTGGTCGCTTTGTCCGTTGCAATGGATGATTTAAAACAGTTGATTGACCTCAACTTAATTCAAGCTG

TTGAAATGAATATTAAAGGAAAACAAAGAAATATCGCCTGCCCAGTGCTATCAGAGAATTCAACTTCTCTGAAARTAAGA

GGCCAGAGAATCRTCTTGCTGATCTTCTTGACAASAAGTCTGATTGTTTCAATGATATTCATGGTAACGGTACTTTATCA

CCAACAAATCAGCAAACGTACAAAGCTTTAATCTCCTATCTCTCATTTGATACTCGAGAAGGAAATATACCTGGAGAAGA

CATAGGGAAGTTTCTTCAAAAGGGCATTGCAAGTGGTTGTTTCCAAAGTTTAGAAGTTCTTGATCTTGAACATGTATTCA

GGCCTCAATTGCCCAAAACCTTAGGAAAATTAGGTCATCTCATGTATTTAGGCTTAAGGTGGACTTACCTGGAGGAAATC

CCACCATCCATAGGCAACCTAGTGAACCTTCAAACTCTGGATGTGAAGCATACTTATGTCACAACTCTACCTAAGTCCAT

ATGGAAATTGCAGAAACTTGAACGCCTATACTTGAACGAGAGTCATCGAACTAAATTCGTATCTCAACCAAGGAGCAGTT

CCCTAAAGGATCTACAAATATTAWGGGGTTTGTTCTTGGATAATGCAAGTGATATAAAGAATGGGCTGAACAAGCTGAAC

AAGCTTAAAAAACTAGGACTGGCATTCGAGCTAGAGTTGTCACAGCAACACGAATTGGCAAATTGGGTGAAAGGTCTGAA

TTCCCTCGAATCTTTGAGATTGAGATCAGTTGACGAGAACGGTGATGCTCAGGATCTTCGCTTGGATAATTTGTCAGGCC

TTAAAAATCTCTCCAGACTATATTTGTTTGGAAAGCTAAAGATTCCTTCCATCAAAGATCTATTTCCAGACACTTTGTCT

GACCTTACGTTGTCTGCCTCGGGGCTTGAGGATGAYCCAATGCCAACATTAGGAAAGCTCAACAGTCTTAAATCACTCTG

TTTCTACTTTGACTCTTATAAAGGAAAACGGATGGTTTGCCAGGGCTTTTCCCAACTTCAAGTTTTGAAACTTTGGAATC

TAAAGGAGCTAGAAGAGCTGAAGGTGGAGAAAGAAGCAATGCAAAAACTGAGGGAGCTAGAGATCAGGCGCTGCGAAAAT

TTGAAGGTCTACACTGGTTTTAAGCACTTAACGAGTCTCCAAGAATTGAAATTGACAAAGATGTCATCAAATTTCACCAC

AGAKATAGAGAAAARGAGGTTAGAAATTTGGTATGCTCACCCTCCCAGGATCACAAAGTCAACTGGTAGTCCGAGTTGA
