# Supplementary material for: Decoding drought tolerance from a genomic approach in Castanea sativa Mill
Source: Plant Genome. 2025 Nov 9;18(4):e70116. doi: 10.1002/tpg2.70116 (PMC12598267; doi:10.1002/tpg2.70116)
Supplement: Supplementary file 6 — Data S2. CDS of candidate genes for drought tolerance in C. sativa sequenced using Sanger methodology. [file TPG2-18-e70116-s001.docx]

>CG1 [organism=Castanea sativa], CDS

ATGGATGTTGAAGCAGTTGTCTCTATAGTGATACGGAAACTCACAGACCTGCTCATTCAAGAACCAATCATCTTCAACAA

GGCTATAGATGAGATAGAGCAAGTCAGAATCAGCTTAAGACAGATGCAGAGCTTCCTAATAGATGCAGAAGATAAAAAAG

AACACGAAGAAGGTGTTAAGAAGTGGGTGGATCAATTTCTTGCTGTTGTTTATGAAGTGGAGGATGCCATTGAAACGTTT

GTCCTGTGGAAAATGTATGCAAGGAGAATGGGGTACTTTTTCATCCCCAAGAATTTGAAGGCTGACTCTGACCTTCGCAA

TAAGATAGAGGAGATCAAGCACAAGATAAAAAACTTTGACAAAATGGAGACAGGTGGGGTCTCAAACCGAGGCGAGCCTC

TCATGATTCGGCAATTAACTTTGCACAGGTCAAGTTATCGAGATGAAGGGCTAACACTGAATCAGCAAAACAATGATTCC

AGCTATTCTGTGGATGAAGAAACAGACTTCATTGGCTTCGAGAAAGACAAAAGCAAGCTGGTGGCCATGCTAACTGGCAG

TGGCCCGGAATCCGGGGATTACCAAAAGTTTCCTGCCATTTCAGTTGTAGGCAAGCATGGCACTGGCAAGACTATCCTTG

CAAAGGCAATTGAGGTTAAAAGTCGTCTTGATCGTCGCGTCATTTCAGTTGTAGGCAAGCATGGCTCTGGCAAGACTACC

CTTGCTAAGGCAATTTATAAAAGTCCTGAGGTTAAAAGTGATTTTGATTGTCGCGCATGGGTTCTTGCCTCTGGGAACCT

TACAGATGTGCTGTTGAGCATATTGGAACAGATTGTCAATATAACTGTTGATAATAAATCTACGAAAGAAGAATTGACAC

AGAAGATCCATATGAATTTGCAGGAGAAACGGTACCTGGTGGTGTTGGACGATTTACAGACGCCTAGTTTATGGAAAGAA

CTTCTTGAGGCCTTTCCAGACACAAATAAAGGTAGCAGGATCATATTAACTACCATCAATTATCGCGTCGCTTTTTCAGC

AGATACAAGAGGAGAGCCTCACCGGCTCAATCCTCTGAATGAGAAAGACACCTGGAAATTATTCTTGAAAAAGGTACGCT

TACCAGACAACTTAATGTTCTCTGATGAAGAAGTCTTCAAGCTTAAAAGGAAACTTATAAGAAGGTGTAAGGGCTTACCT

CTGGCAATCGTGGTGCTTGGAGGTTTGCTGTCGACGAAGGATCCGAACTACGAAGTATGGTTGAAAGTTTTTGAGCACCC

AAGTTGGCAGCTAGACAGAAAGCAAGACCAGTTCTCAATTATATTGGCCCTGAGTTATAATGATCTGCCCTTCCACTTGA

GGCCTTGCTTCCTCTATTTACTGCTTTTCCCAAAAGATAAAGATATTCCTGTGAGAAGGTTGCAGCGGCTATGGCTTGCA

GAGGGATTTGTGAAGCATACACCCGAGAAGACTCCGGAAGATATGGTGGAGATATACTTCAAAGAGCTTGTGCAAAGAAA

CTTGATTCAAATATTCAAATGGAGAAAAGACGGGAGTCCCAAAACATGTCGTATCGGTGTCTTACAAGATGACGGTAATT

TCATGTCAAAAGCTCAAGAGATTGGCCTTTTCCACATCCAAAAAATGTCAGAAAAGGAAACTCCTCAGCATGGTGTTCGC

CGGGTTACTGACTTTGTTGACCCCGAAGAGTACATCACACAAATTCAAAATCTGCGCTCTTACTTGGCCTTTGACATTCA

AAAAAATGATATGCCTGCGACAGAAATTGATAGGTTTCTCAACAAAGTCACTGGTCAAAATTTTGGATTGCTGAGGGTGG

TTGATCTAGAGCGTGTTTACAAGCCTAAACTGCCCGACAATATGGGGAAACTGTTTCTCTTGTTGAGGTACTTGGGCTTA

AGATGGACCTTTTTGGATGCCCTTCCCCACTCTGTTGGTGAGATGCCTTACCTTGAGACTTTGGATGTGAAGCACACATA

TATCAGCTCTCTACCGAGTTCTATTTGGAAGATGAAACACCTGCGCCATCTGTGTCTGAATGAAATACGTCTTGACATGT

CTCTGCAAAACCATGGCACTTCTCTTACTCATCTCCAGACATTATGGGGATTATTCGTAGACAAAAAGACTCCAGTGAAG

AATGGTTTGTATAGGTTAATCAATCTCAGGAAATTGGGTCTGACATGTCATTTAGATTCCTTTCAAGAATTAGATGAGTG

GATTGCAAGACTTGCAAGTCTTCAATCTCTCAGGATACGATCCAAGAACCAAAACGGTCAACCTTGGAAGCTCAATTTGA

AGCCTCTGTCAAGCCTTGAGAATCTCACCAACCTGTATTTGCTTGGAAGCTTACCGGAGCTACATGATAGGTATGAATTC

CCTCCTAAACTTACAGTTCTTACTTTGTCGGTCTCAAAGCTGGAGAAAGACCCCATGCCAATCCTAGCGCAGCTGCCAAG

CCTCTCTGTCCTCAGGCTATTGGCAGATTCCTACACTGGGAAGGAATTGAAGTGCCCACCCAAAGGGTTCATTAAGCTTC

AAATTTTGAAGCTTTGGATGCTAAAGGATTTGGAGACATGGGAGGTGGGGAAAGAAGCACTTAAGGAACTCCAAGAAGTA

GAAATCCGATGCTGTGACAAGCTAAAGGAACTTCCAGCCCCATTGTTTATTTTGGAAAACATTAAGAAAATAGTCTTAAC

GAACATGCCACAGAAATTTGTAGATGAGATTCCAGCCAAAGGATTAATCTTCCCCAAAACTCTGCAGTTCTGA

>CG2 [organism=Castanea sativa], CDS

ATGGAAATTACCTCTCATCTCCTTGCAATTGCAGGGGCTTTTGTACTGGTACTATTGTACAATCTATGGAGGCTGAGAAT

TGGTAGTCACAAAAGTAAGGGTATGTTGGCCCCGGAGCCATCAGGTGCGTTGCCAATCATAGGTCACCTTCACAAACTAG

GTGGCCAAAACCCAATTGCCCGAACCTTGGCAGCTATGGCTGATCAATATGGTCCCATATTCACGATCCGATTTGGTATG

AAAAGTGCAATTGTTATTAGCAATCATGAGGCTGTCAAGGATTGTTTCACTACAAATGACAAGGCTTTGGCTGCACGTCC

AAGGTCTAGCCATGGAAAGTACCTTGGCTACAACTATGCAGGATTTGGGTTCATCAACTATGGAAAATTCTGGCTCAAGA

TGAGAAAGATAACCATGCTAGAACTCCTCTCTAGTCGTCGGCTTGAGACACTAAAGAATGTGCAAGATACTGAGGTCGAC

AATTTGATTAAAGATTTGTACACTCTTTGCATGAGCAAAGAGCACAACAACCAAGCTAAGGTGGTGATTAGTGAGTGGAT

TGAGCGTTTAACCTTCAATATAATTACTAAAATGATTGCAGGGAAAAGATACTTTGGAAACTTGAATGATGGGAATGACG

GAGAGGCACAAAGAATAGGGAAAATTATTAAAGAGTTCATGTATGTATCTGGGGTCCCTGTTGTTTCGGATCTTGTTCCA

TTTCTAGGAGGGCTTGAATATTTTCTGGGCCAAGTAAAATCCATGAAGCGAATTGCAAGGGAATTGGACACTCTAGTTGG

AAGTTGGGTTGAAGAACATGCTATGAGGAGGGCGGAAAGTGAACGAATTGACAAACCGGACTTTATTGATATCATGTTAT

CTGTAATTGAGGATGACGGCATGTTTGGCCATACGCGTGAAACCATTATCAAGGCAACAGCATTGAATCTTATCTTGKCT

GGGTCTGACACTACATCTCTTAACTTGACATGGCTCTTGTCTATATTATTGAACAACAAGCACGCTTTGAAGCAAGCCCA

AGAAGAGCTCGATCTCAAGGTTGGTAGAGAGAGATGGGTGGACAATCATGATATCAAAGACCTARTTTACCTCCAAGCCA

TAGTGAAGGAAACCTTGCGCTTGTACCCACCAGGACCCTTAGCAGTTCCACATGAAGCCATGGAGGATTGTCATGTATGT

GGCTATTATGTTCCAAAGGGTACTCGTGTTTTTGTCAATGTGTGGAAGTTGCACCGAGATCCAAGGATTTGGGATGACCC

AGATAAATTTATTCCAGAGAGGTTCCTCACAAAACATGCAAATATAGATGCTTCAGGGCAACATTTTGAGTTCGTACCAT

TTGGGTCTGGTAGACSATCTTGCCCAGGTTACACATTTGCCTTGCAAGTATCTCACTTGACACTAGCTCGATTACTTCAG

GGATTTGAGTTCACGACACCATTGAATATGCCAATAGACATGACTGAAGGCTTGGGCATTACCTTACCCAAGGCAACTCC

CCTTGAAGTTCTTCTCAATCCACGCCTTGCTCCGGAACTCTATCAATGA

>CG4 [organism=Castanea sativa], CDS

ATGGCGTCGGAGACTGAAACGTTCGCATTCCAAGCCGAGATAAACCAGCTTCTCAGCTTGATCATCAACACCTTCTACTC

CAACAAGGAGATCTTTCTTCGTGAGCTTATCAGCAACGCTTCCGATGCTCTGGACAAAATCCGTTTTGATAGCTTGACTG

ACAAGAGTAAGCTCGAGGCGCAGCCTGAGCTTTTCATTCATATTATCCCTGACAAGGCCAGCAATACTATCACCATCGTT

GACAGTGGGATTGGAATGACCAAGGCTGACCTGGTAAACAATCTTGGAACCATTGCAAGATCTGGAACCAAGGAGTTTAT

GGAACACTTGGCTGCTGGTGCGGATGTTAGCATGATTGGTCAGTTTGGAGTTGGGTTTTACTCTGCTTACTTGGTTGCTG

AGAGGGTGGTTGTTACCGCAAAGCACAATGATGACGAACAGTATATCTGGGAGTCTCAGGCTGGTGGTTCTTTCACTGTA

ACTAGGGACACTTCTGGTGAGAACCTTGGAAGGGGTACTAAGATCACCCTTTTCCTCAAGGAAGATCAATTGGAATACCT

TGAGGAGCGTCGTCTTAAGGACCTTGTCAAGAAGCATTCTGAGTTCATAAGTTACCCAATTTCCCTTTGGGTGGAGAAGA

CTGTAGAGAAGGAAATTTCAGATGATGAAGATGAAGAGGATAAGAAGGAGGAGGAAAAGAAGGAGGAGGAGGAAGGTAAG

GTGGAAGATGTTGATGAAGAAGAAAAAGACAAGGAAGAGAAGAAAAAGAAGAAGATCAAGGAGGTGTCCCATGAGTGGTC

ATTGGTCAATAAGCAGAAGCCCGTCTGGATGAGGAAGCCAGAAGAGATCACTAAGGAGGAGTACTCTGCATTTTACAAGA

GTCTCACCAATGACTGGGAGGAGCACCTTGCTGTGAAGCATTTCTCAGTTGAGGGTCAGCTTGAATTCAAGGCTATCCTC

TTTGTTCCCAAGAGGGCACCTTTTGACCTATTTGACACAAAGAAGAAGCCCAACAACATCAAGCTCTATGTCCGCCGTGT

CTTTATTATGGATAACTGTGAGGAATTGATTCCTGAATATTTGGGTTTTGTGAAGGGTATTGTGGATTCTGAGGATCTGC

CTCTCAACATTTCAAGAGAGATGCTGCAGCAAAACAAGATCCTCAAGGTTATCCGGAAAAATCTTGTCAAGAAATGTATT

GAGCTCTTCTTTGAGATTGCTGAGAACAAGGAGGACTACAACAAGTTCTACGAGGCATTCTCAAAGAATTTGAAGCTTGG

TATTCATGAGGATTCACAGAACAAAACCAAAATTGCCGAATTGCTTCGGTATCACTCCACCAAAAGTGGAGAGGAGATGA

CTAGCCTGAAGGATTACGTCACAAGGATGAAGGAGGGGCAGAGTGATATCTACTACATTACTGGAGAGAGCAAAAAGGCT

GTAGAGAATTCCCCATTCCTTGAGAAGCTGAAGAAAAAGGGATATGAGGTTTTGTTTATGGTTGATGCTATTGATGAGTA

CGCCGTGGGTCAGTTGAAGGAGTTTGAGGGGAAGAAGCTTGTCTCTGCAACCAAGGAGGGACTCAAGCTTGAGGAGAGTG

AAGATGAGAAGACTAAGAAAGAAGCATTGGCTGAGAAATTTGAGGGACTTTGCAAGGTGATGAAAGATGTGCTTGGTGAC

AAGGTTGAGAAGGTGGTGGTATCTGATCGTGTGGTGGATTCCCCCTGCTGCTTGGTAACTGGAGAGTATGGATGGACAGC

AAACATGGAGAGAATTATGAAGGCTCAAGCACTGAGGGATTCAAGTATGGCTGGTTACATGTCTAGCAAGAAGACCATGG

AGATTAATCCAGAGAACCCCATCATGGAGGAACTGAGGAAGAGGGCTGAGGCAGACAAGAATGACAAGTCAGTGAAGGAC

TTGGTTCTATTGTTGTTTGAGACCTCTCTTCTTACATCTGGCTTCAGCTTGGATGAGCCCAATACTTTTGGCAACCGAAT

TCACAGAATGCTTAAATTGGGATTGAGCATTGATGAAGACACTGCTGAAGCTGACACTGAGATGCCTGCTCTAGAGGAGG

CCGATGCTGACGCAGAGGGGAGCAAGATGGAGGAGGTCGACTAA

>CG6 [organism=Castanea sativa], CDS

ATGGCAGGCTCTATGACCATTTGTTTCTTTGTGGTGCTTTCGATCTCTATTTTGTTATCTTGGACCAACCCAAGAGGMGA

AACCCTAAAATCCTTCTCTCTCTCTCCTCTCTATTCKTCTACACAGTCGGCTTCTTCTTCCAATGAGGATATCTCCGGCT

TGAGGTACGATTTCTACCATGAAACTTGTCCACAGGCAGAGTCCATTGTAAGGACTCACATGGCTCGCTTATATTCCGAC

CATAACAATGTCTCTGCAGCTCTGCTGCGTCTTTTCTTCCRTGASTGCTTCATWGAGGGCTGTGATGCTTCTGTCCTCTT

GGATGACAGCAATGGTGACAAAAACCATTCCATAGAGAGGCAGGCCATACCCAATCAGACCTTGAGGGGTTTTGATAAAA

TTGATTCCATCAAGGAGGAGCTTGAAAAGGCTTGTCCAGGGGTAGTTTCATGTGCTGATATTGTTTCTATTGCCACTAGA

GATGGCATTATGCTGGCTGGTGGCCCTTTCTATCCAGTTTTAACAGGCCGGAGGGACAGCATTCATTCATATTTCAATGA

AGCACTGGCCGCGATTCCACGACCCGATGATAACATAACACAGACCCTTCACCTTTTTGACCTTAGAGGTTTTGATGAAA

GAGAGGCTGTCAGCCTTCTAGGCGCACACAACATTGGGAAGATTGGCTGTGAATTCATACAGAAACGTCTCTTTAACTTC

AAGGGGACAGGGCAGCCAGASCCAACTATAGCTCCTGATTTCCTAACTGAGATGAGAATGAGATGCCAAGACAGKAATAG

GACCACCACTCGACCTTCTTCTTCATCCATGGCATCATTGGAAATGAGTGAGTCAGCAGKGGGGATGTCATATTTGCAGG

CATTTTCATCTTCGATTTCATCTGGGGCAGGTTTTGATACTCACTACTATCAGAGCTTGTTAAGTGGGAGAGGACTCCTT

TTTGCTGATCASCWATTAATGGCTAATGAGAAGACTGCRAGACTGGTAAGAGCTTATGCTTCAGACGATGGATCAACCTT

TCGAATGGACTTTGCACGGGCAATGATGAAGATGTCAGGCCTTAATGTTCTGACTGGATCTCAAGGTCAGGTCCGACGGA

ATTGCTCCTTGCCTTTGGTCAGTTCCTAA

>CG11 [organism=Castanea sativa], CDS

ATGGSGGATWCTGAGACGTTTGCTTTCCMGGSTGAGATCAACCMGTTGTTGAGTTTGATCATCAACMCTTTYTACAGCAA

CAAGGAGATYTTTYTTYGTGAGCTTATCAGCAAWGCCTYTGAWGCTCTTGACAAGATTCGATTTGAGAGCTTGACCGACA

AGAGCAAGCTAGATGGTCAACCAGAGCTTTTCATCCACATCATTCCTGACAAGACCAACAACACCCTGACCATTATTGAC

AGTGGTATTGGAATGACTAAAGCTGATTTGGTGAACAACTTGGGTACCATTGCTAGGTCTGGAACCAAAGAGTTCATGGA

AGCCCTTGCAGCTGGGGCTGATGTGAGCATGATTGGTCAGTTTGGTGTTGGTTTCTACTCAGCATACCTTGTTGCTGAGA

AGGTTATTGTCACCACAAAGCACAATGATGATGAGCAGTATGTGTGGGAGTCACAAGCTGGTGGGTCATTCACTGTTACC

AGAGACAACTCTGGTGAGGTCCTTGGTAGAGGTACCAAGATTACTCTCTACCTCAAGGAGGACCAGCTTGAATACCTTGA

GGAGCGCCGCTTGAAGGATTTGATCAAGAAGCACTCTGAGTTTATCAGCTACCCAATTTCCCTTTGGATTGAGAAGACCA

CTGAGAAGGAGATTTCTGATGATGAGGATGAGGAAGAGAAAAAGGATGAGGAAGAGAAGAAGGACGAGGAGGGTAAGGTT

GAGGAGATCGACGAAGAGAAGGAGAAGGAAGAAAAAAAGAAGAAGAAGATCAAGGAGGTGTCCCATGAGTGGTCTTTGGT

TAACAAACAAAAGCCCATCTGGATGAGGAAGCCTGAGGAGATCGCAAAGGAAGAGTATGCTGCTTTCTATAAGAGCCTTA

CAAATGACTGGGAAGAGCACTTGGCTGTGAAGCACTTCTCAGTTGAGGGTCAGCTTGAGTTCAAGGCTATCCTCTTTGCA

CCAAAGAGGGCACCTTTTGATCTTTTTGACACAAGGAAGAAGCCAAACAATATTAAGCTCTATGTTCGTCGTGTATTTAT

CATGGACAACTGTGAGGAGCTGATCCCTGAATACCTTGGATTTGTTAAGGGTATTGTCGATTCTGAGGATCTTCCTCTCA

ACATTTCAAGAGAGACCCTTCAGCAGAACAAGATCCTTAAGGTCATCCGCAAGAATTTGGTTAAGAAGTGCATTGAGCTC

TTCTTTGAGATTGCTGAGAACAAGGAAGACTATGCTAAGTTCTATGAGGCTTTCTCTAAGAACCTCAAGCTTGGTATCCA

CGAGGATTCCACAAACAAAACAAAGCTTGCCGAGTTGCTCCGCTACCACTCCACCAAGAGCGGTGAAGAGATGACCAGCT

TGAAGGACTATGTGACCAGGATGAAGGAGGGCCAGAGTGACATCTATTACATCACTGGTGAGAGCAAGAAGGCTGTTGAG

AACTCTCCATTCCTTGAGAAGCTGAAGAAGAAGGGGTATGAGGTTCTGTACATGGTTGATGCTATTGATGAGTATGCTGT

TGGTCAGCTTAAGGAATTCGAGGGCAAGAAGCTTGTTTCTGCTACCAAGGAAGGTTTGAAACTTGATGAGAGTGAAGATG

AGAAGAAGAGACAGGAAGAGTTGAAGGAGAAGTTTGAGGGTCTCTGCAAGGTGATCAAGGATGTTTTGGGTGACAAGGTT

GAGAAAGTTGTGGTCTCTGACCGTGTTGTCGATTCTCCCTGCTGTTTAGTGACTGGTGAGTATGGATGGACTGCCAACAT

GGAAAGAATCATGAAGGCACAGGCTTTGAGGGACAGCAGCATGGCTGGCTACATGTCAAGCAAGAAGACCATGGAGATCA

ACCCTGAGAACTCTATCATGGAGGAGCTCAGGAAGAGGGCTGATGCTGACAAGAATGACAAATCAGTGAAGGACCTTGTT

CTCCTGCTTTTCGAGACTGCCCTCCTCACCTCAGGCTTCAGCCTTGACGAGCCCAACACCTTTGGCAACAGGATCCACAG

GATGCTGAAACTCGGTTTGAGCATTGATGAGGAAGCAGCTGATGGTGATGCTGAGATGCCCCCATTGGAGGAAGCTGATG

CTGATGCTGAGGGCAGCAAGATGGAGGAAGTTGATTAA

>CG13 [organism=Castanea sativa], CDS

ATGGCTTCCTCTTCTGGGAGCTTGGATACCTCAGCTAACTCACACCCGGCTAGCTTCACTTTCTCTACTCACCCTTTCAT

GACAACCTCTTTCTCTGACCTCCTTGCCTCACCCAACGATATTGATAACCAAAACACCACATTAGAGAATAGAAATCGTA

GCTTGTCAGATCGTATAGCTGAGAGAACTGGGTCGGGTGTGCCTAAATTCAAGTCAATCCCACCTCCTTCTTTGCCCATT

TCTCCACCTGCTGTGTCTCCAAGCTCTTACTTTGCTATCCCAGCTGGGTTGAGCCCAGCTGAGCTCTTGGACTCCCCTGT

TCTTCTCAACGCTTCTAACATTCTGCCATCTCCAACAACAGGAACATTCCCTGCTCAGGCCTTCAATTGGAGGAGTAATT

TTAGCAACCAGCAGCAGAATGTTAAACAAGAAAACAAAAACTACTCAGATTTCTCTTTCCAAACTCAACAAAGGCCTCCT

TTATCATCAACAACAAACTATCAGCCTTCAAATACTGCGATTCAAACTGTACAGCCACAAGCCTGGAGTTTCCAAGAACC

CACCAAGCAGGATGATTTTTCCACAGGAAAGAGTATGGTAAAGGCTGAATTCGGTTCAATGCAGGGCTATTCCTCTGATA

TCACCACCATAACAAGTAACATTCAAAGCAACAATAACAACAACAACAACAACAGCAACAACGGTGGGCTCCAATCAGAA

TATGGTAATTATCACCAACAGCCTCAACAGCCTCAAACACTGAGTAGGAGGTCTGATGATGGGTACAATTGGAGGAAATA

TGGGCAAAAACAAGTTAAAGGAAGTGAAAATCCTAGAAGTTATTACAAGTGCACATACCCCAATTGCCCAACAAAGAAGA

AAGTTGAGAGGTCCTTAGATGGGCAAATTACTGAGATAGTTTACAAGGGTAGTCATAACCATCCCAAGCCTCAGTCTACT

AGGAGATCATCCTCAGCTTCTTCTCATGCCATTCAAGTTTCTAATTCTTCCTCCCATGAACTTCATGATCAGTCATATGC

CGCCCTTGGTAATGGACATATGGATTCGGTTGCAACCCCAGAAAACTCTTCCATATCGATGGGGGATGAGGATTTTGATC

AGAGTTCTCAAAAGAGCAAGTCCGCAGGAGATGACTATGATGAGGATGAGCCCGATGCCAAAAGATGGAAAAAAGAGGGT

GAAAATGAAGGTATATCAGCAGCCGGGAGCAGAACAGTGAGGGAACCTAGAGTTGTAGTTCAGACAACTAGTGATATAGA

TATTTTAGATGATGGATATCGTTGGAGGAAATATGGGCAGAAAGTGGTCAAGGGCAATCCAAACCCAAGGAGCTACTACA

AGTGCACATATCCAGGGTGTCCAGTGAGAAAGCATGTTGAGCGAGCATCTCATGATCTAAGGGCAGTGATCACAACCTAT

GAGGGGAAGCACAACCATGATGTTCCTGCAGCCCGTGGCAGTGGCAGCCATTCTGTCAATAGGCCTTTGCCAAGCAACAA

TAATAACAACATTAGTGCTGCCACAGCAATTAGGCCTTCAGCCATTACCCATCACACTAACAATTCTGTGAACAACCACC

ATCTTCACAATCKAAGACTACCAACATCTGAAGGGCAAGCACCCTTCACCTTGGAGATGTTGCAGAGCCCCGAGAGCTTC

GGCTTCTCGGGTTTCGGGAATGCCATTGGTTCCTACATGAACCAACAACAAATCACAGACAATGTGTATTCTAGAACCAA

AGAAGAACCAAGGGATGACATGTTTTTCGAATCTTTGCTTGCATAG

>CG14 [organism=Castanea sativa], CDS

ATGGCTGACGTTCAATSGGGAGACAGAGACACAGAGACCTTCGCGTTTCAGGCAGAGATTAACCAGCTTTTGAGTCTCATC

ATCAACACCTTCTATAGCAACAAGGAGATCTTCCTTCGTGAGCTCATCAGCAATGCTTCTGATGCGTTGGATAAGATCAG

ATATGAGAGCTTGAGAGACAAGAGCAAGCTTGAGAGTCAACCCGAGCTCTTCATTAGGATTGTACCAGACAAAGTCAACA

AGGCCCTCTCAATCATAGACAGCGGTATAGGCATGACTAAAGCAGATTTGGTGAACAACTTGGGAACAATAGCAAGGTCA

GGAACCAAGGAGTTCATGGAGGCGTTGCAGGCTGGGGGTGATGTGAGCATGATTGGGCAGTTTGGTGTTGGGTTCTACTC

GGCTTACCTTGTTGCTGACAAGGTTATTGTGACTAGTAAGCACAATGATGACGAGCAGTACATCTGGGAGTCTCAGGCTG

GTGGCTCTTTCACTATCACCAGGGATGTCAATGGCGAGCCCTTGGGTAGAGGAACAAAGGTCACTCTTTTCCTCAAGGAA

GACCAGTTGGAGTACTTGGAGGAGAGGAAATTAAAGGACCTTGTGAAGAAGCACTCTGAGTTCATCAGCTACCCCATATA

CCTCTGGACGGAGAAGACCGCTGAGAAAGAGGTCAGTGATGATGAAGATGATGAAACCAAGAAGGAAGAGGAGGGTGATG

TAGAGGATGTTGATGAGGAAAAGGAAAAGAAGTCCAAGAAGAAAAAGGTTAAGGAGGTGAGCCACGAGTGGCAGCTCGTC

AACAAGCAGAAACCAATTTGGCTGCGAAAGCCAGAGGAGATCACCAAGGAAGAGTATGCCTCCTTCTACAAGAGCCTCAC

CAATGACTGGGAGGATCACCTTGCTGTGAAGCACTTCTCTGTTGAAGGGCAACTTGAGTTCAAAGCTATCCTTTTCGTTC

CTAAGAGAGCCCCATTTGATCTATTTGACACAAGGAAAAAGTTGAATAACATTAAGCTCTATGTTAGGCGGGTGTTTATC

ATGGACAATTGTGAAGAACTTATTCCTGAGTATCTCAGCTTTGTGAAGGGTGTGGTGGATTCTGACGATCTTCCACTCAA

TATTTCCCGTGAAACGCTACAGCAAAACAAGATTCTCAAGGTGATTAGGAAGAACCTGGTGAAGAAGTGCAWTGAGATGT

TCAATGAGATTGCAGAGAACAAGGAGGACTATGCCAAATTCTATGAAGCTTTCTCTAAGAATATTAAGTTGGGAATCCAC

GAAGATAGCCAGAACAGGACTAAGCTGGCTGACCTGCTGAGGTACTACTCAACTAAAAGTGGTGATGAAATGACCAGCTT

GAAGGACTATGTAACAAGAATGAAGGAGGGTCAGAAAGATATCTATTACATAACTGGTGAGAGCAAAAAGGCTGTTGAGA

ACTCACCTTTCCTTGAGAAGCTGAAGAAGAAGGGCTATGAAGTCCTCTTCATGACTGATGCCATTGATGAGTATGTTGTT

GGGCAGTTGAAGGAATATGATGGGAAGAAGCTTGTTGCTGCCACAAAGGAAGGCCTTAAGCTAGAGGATGACTCTGAGGA

AGAGAAAAGGAAAAGGGAGGAAAAGAAGAAATCATTTGAGAACCTGTGCAAGACAATTAAAGACATTCTTGGGGACACGG

TGGAGAAAGTGGTGGTGTCTGATAGGATTGTAGATTCACCTTGCTGCTTGGTCACTGGGGAATATGGATGGACAGCTAAC

ATGGAAAGGATCATGAAAGCACAGGCTCTAAGGGGCAACAGCATGAGTTCTTACATGTCAAGCAAGAAGACAATGGAAAT

TAACCCAGATAATGGCATTATGGAAGAGTTAAGGAAAAGGGCGGAGGTTGACAAGAATGACAAGTCTGTGAAGGACCTAG

TGTTGCTGCTCTATGAGACTGCCCTCTTGACTTCTGGGTTCAGCCTCGACAAACCCAACACATTCGCCTCAAGAATTCAC

AGGATGCTGAAGCTGGGTCTGAGCATTGAGGAGGATGAGAGTACTGGTGAGGATGCTGAAATGCCTCCATTGGAGGAGGA

TGGCAATGAGGAGAGCAAGATGGAGGAAGTCGACTGA

>CG17 [organism=Castanea sativa], CDS

ATGGCTATGTTTCAGAAACAAGAGCTTGAAGAAGAATACAGAGGCGCCACGAGAACCGTCGATCTCAGAATCAGCGAAAT

CAGCGGTGCACAAGAAGCGGGGGCCACGTCGCCGCCTAAGCCGAAGGCAGAGCCGGACTCGGAGGAGGAGGACGAGGAGA

ACATGTCACGTGCGATGCAAGTCATGCCAGTAGCTATGCACGTGCCTTCGGGAATTCCCATGACCAAAGCGGTGGCTAAG

AGAGGCTCCACCAAGGACCGCCACACCAAGGTGGAGGGTCGCGGCCGGAGGATCCGAATGCCCGCCACTTGCGCCGCTCG

GATCTTTCAGCTGACCCGAGAACTCGGCCACAAGTCCGACGGAGAAACCATCCGTTGGCTACTCGAGCACGCCGAGCCYG

CCATCATCGCCGCCACCGGAACCGGTACCGTCCCCGCCATCGCCATGTCCGTTAATGGGACTCTCAAAATCCCAACCACG

CCCGCTCCAACGTCCGACCCGAAACCCGGCGACCCACCCGTTAAGAAGAAACGCAAACGACCCGCCAACAGCGAATACAT

AGACATAAACGACGGCGTTTCGGTCTCCTCTGGGCTCGCTCCAATATCAACAGCAACAGCAGCAGCAACAACAACAACAA

CAACCATGCCTCAAATACAAGCAGTGCCTCAAGCTTTGATTCCCATGTGGGCTATACCATCAAACGCCGTCGTTCCGGGC

GCGTTCATTATGGTCCCTCCGATGACGTCAATTCCCGGAGCTCCGAACCCGGCTCATATATTCACATTCCCAGCCACTGC

AACGCCTTTGATTAACATTTCGGCCCGACCCATATCGTCGTTTGTGTCCTCCATGGCTAATATAGCCCCCCCAGTTCAAA

TCCAAGCCAGCTCAGCAGCTTCGTGTTCAAGCTCTGCTACAGCTATTACAACAAGTACAACAACAATTGCTACAACTAGT

GCTCCCACTGCGACTATTTTAACCACTCAGAAGCTTAGAGACTTTTCGCTCGAGATTTACGATAAACAGGAGCTTCAGTT

CATGTCTCGGTCTTCGAAACACTGA

>CG18 [organism=Castanea sativa], CDS

ATGGAAGAAGTGAAGCTGCTTGGATTTTGGCCAAGCCCCTTTAGCTATAGAGTGATATGGGCTCTGAAGCTGAAGGGTGT

GAAATATGAGTACATAGAAGAGGACCTAAGCAACAAGAGTGATAGGCTACTGCAGTACAACCCAGTTCACAAGAAGATTC

CGGTTCTTGTTCATGGTGGCAAACCTATAGCTGAGTCTCTAGTTATCCTTGAATACATCGAAGAAACTTGGCCTCAGAAC

CCTTTGCTGCCAAAGGATRCCTATGAAAGGGCATTAGCTCGGTTCTGGATTAAATTTGGAGCAGATAAGGGTCCTATTTT

CTCTGCATTTTTCCGATCTACAAAAGAGGAGCTTGATCAGAATGCAATAAAAGAAATGGTGGAATATCTGAAAATCTTGG

AAGAGCAAGCTCTAGGGGACAAAAAGTTTTTTGGTGGCGACAATATAGGATTGGTAGACATAGCATATGGATGGCTGTGT

CACTGGTTCATAGGCATGGAAGAAATGGGAGGAGTTAAACTGCTAGGACCAAGTACTGTGCCTCGCTTGCATGCATGGGC

TGAGAATTTCAAGCAACTTCCTATAATCCAAGAGAACCTACCTGACTATACAAAAATGTTGGCACATTTTAAATCATTGA

GGGAGAAAAATACTGCCTCTGATGCCCGTTAG

>CG20 [organism=Castanea sativa], CDS

ATGGACAAGTACGAGGGAGTGAAGGATTTGGGGGCTGGGAATTTTGGTGTGGCAAGGCTCTTGAGGCACAAGGAGACCAA

GGAGCTTGTTGCCATGAAATACATCGAACGTGGCCTCAAGATTGATGAGAATGTGGCTAGAGAGATTATCAACCACAGAT

CACTTCGGCACCCCAACATAATCCGATTCAAGGAGGTGGTTTTGACCCCCACACATTTGGCTATTGTGATGGAATATGCA

GCCGGCGGAGAGCTTTTTGAACGAATCTGCAATGCCGGTAGATTCAGTGAAGATGAGGCTCGATACTTTTTTCAGCAGCT

GATCTCTGGTGTCAATTATTGTCATTCCATGCAAATATGCCATAGAGATTTGAAGCTGGAAAACACACTTTTGGATGGAA

GCCCTGCACCACGCTTGAAAATTTGTGATTTTGGTTATTCTAAGTCATCTCTGCTGCATTCAAGACCCAAATCTACAGTT

GGAACTCCGGCATATATTGCACCGGAGGTGCTTTCACGGCGAGAATATGACGGCAAGATGGCAGATGTATGGTCATGTGG

AGTGACTCTCTATGTTATGCTGGTGGGAGCATATCCTTTCGAAGACCAAGATGATCCCAGGAATTTTAGGAAAACAATTC

AGAAAATAATGGCTGTTCAGTACAAAATCCCTGACTATGTTCACATATCTCAAGATTGCAAACACCTGCTTTCTCTCATA

TTTGTTGCAACTCCATCCAGGAGAATTACACTTAAAGACATCAAGAACCACCCTTGGTTTTTAAAGAACTTGCCAAGAGA

ACTAACAGAGTCTAATCAAGCTATCTATTACCAGAGAGACAACCCAAGCTTCTCTCTTCAAAGCGCAGATGAGATCATGA

AAATTGTGGGGGAGGCAAGAAACCCACCTCCATCATCTAGGCCTGTCAGGGGCTTTGGCTGGGGAGCTGAAGAAAATGAG

GAAGGCAGTGAAGACATAGATGCAGAGGTGGAGGAAGAAGATGATGAAGAAGATGAGTATGATAAGAGGGTCAAAGAGGT

TCATGCAAGTGGAGAATTTCATATCAGTTAA

>CG21 [organism=Castanea sativa], CDS

ATGGGTATTAGGCTAGTAAATGAAATGGAAGAGGGGAGAGAGAGAGAAGGAGGGAAGGTGTTGAGTCTGATAGAGAAGGC

GACCAACTCCACAGCTGCTGAGGTGGACCCACGTCTCCTTAAGGCCATCAAATCCGTAGTCTGCTATTTGGATACGGAAC

TCCAACTTGCCGCCAATACCCTTTTGGATCTCATGAAGCGCGACCACTCTCAGGTAAGGTACCTGACACTCCTGATAATT

GATGAACTGTTCATGCGTTCGAAGCTTTTCAGAAGCATTCTTGTTGACAACTTGGATCAGGTGCTGAGTTTGAGTGTTGG

ATTCAGAAGAACTCTGCCTCTCCCTGCTCCTCCTGCTGTCGCTTCCATTTTGCGCTCTAAGGCAATTGAATTCTTGGAGA

AGTGGAACTCTTCCTTTGGGATTCATTACAGGCAGCTCAGATTAGGGTTTGATTACCTTAAAAACACCCTCAAGTTGCAG

TTTCCTAATCTACAGGCCAATGCAGCTCGGATTCAGCAGGAGAGAGCAGAACGGGAAAGGCGGTCAAGAGAGATTTTGCT

AAAGAAATTTGAAATGTTCAAGGACAATTTCTCATCTATTAAGGAAGAGATCATGTCTACCATTGACGAGATTGGGGAAT

GCTTAGACATAGTCCGTACAAATGAGGAGTTTACGCCTCTGCCTCCTGCAGATGATGAATATTTCGAAGAGTTTCGTTCT

TCTGAACTGCTGCAAATCCGTCTCAATACTTTAAAAGAAGGGGAAAAGGTTCACGAGAACAGTGACAATAAAGTGGTTTT

TGATGCATTAAGGGAGCTGTACAAGCTTCTAGAGACAAAGCATTTGGTTTCAGTTCAAGAATGGATCTCTKTTCTTCTAA

GGGTTGAAGTGGCAGACAACAGGTTCAGAGATTCCGCTTTAAAGGAGTTAATYGATATCCAAAATCGTCTCAAATCRGTG

AAGAAGAAGTGTGAAGAATCAGGTTGTGCYCTTCCAAACACTGCARATCGYGATGAAGAAGAAGATGATTTCTGGGAGGA

GGGAAAGATTGGWTCACTTGAGAAYGAGAGATCTACTGTTCCCAATAATCAMGATGAATATTTTTCCATGAAARTAACTT

CTAATAAGTCCAAAAATARAACTCCTGAAAGCAGTAAAAAAGATTGTAATGACAATGAGATTCTCWGTCCTGAAGGYGGT

GAAACCAATTTGGACCCTTTAAGAAGTAAGCTTCTGGCTGAAGCTCCTGTGATGAAGTGGGGCTCTTTCTTGGATAACTG

GGGTTCACACAGGAAGGTTTTGGCTAACCAGCGGGGATTGGAGCTTGAAAGTCACTGGGGTAGGGTGGATTATGACGCGG

TTATTCTAGCTGAGAAAATGCCCGAACTGAATGTACATGCAACTCTTTATGAAGAGCAGCAAACTGACATTCAACCCTGC

AGGGCTCCTTTGAGCAAAGGGGGGCTTTGTCAGAGAAGAGACCTGAGAGTTTGGCCATTTCATGGACCTATTATACCTCG

AGATGATGAAGGAAAGCCACTCAATCAGAACTCTTTAAAAGAAGAGATATCTCTTGATTTGGGGATTGATTCCATTGAGC

AGTTAGCAAAACAAGCTGTGAAGAATGTTCGTGAGAGAGATAAAGAAGTAGCAAATAAGAGAGAAATTGATAAAAAGTCA

CTGAAGCATGCAAAACTTGCAAGAATTCGGGAGCACAATGAAACAGCTCTAAGGGATGCTGCCTTGACATCAACTTCAAG

ATCTGCATCTGTTGGAGAAGATATGGGGGTGACTGATGGTGAGAAACCGTCAGCTAGAAACAAGAAGGAAACACTCTCAT

CCATGCTGCACAAGAAAGTGACACCAAAAGATAGGATAGCTCAGAGGCTTTTGAATTCACGGGCAAAGGATTCAACAACA

AGACAGCTCACATTGGGTGAAGATGCAAATTACCGAGAAGCCTTCCCAAATCAATGGCAATGA
